# Comparative genome analysis of mycobacteria focusing on tRNA and non-coding RNA

**DOI:** 10.1186/s12864-022-08927-5

**Published:** 2022-10-15

**Authors:** Phani Rama Krishna Behra, B. M. Fredrik Pettersson, Malavika Ramesh, Sarbashis Das, Santanu Dasgupta, Leif A. Kirsebom

**Affiliations:** Department of Cell and Molecular Biology, Uppsala University, Biomedical Centre, Box 596, SE-751 24 Uppsala, Sweden

**Keywords:** Mycobacterial genomes, Core gene phylogeny, tRNA and non-coding RNA

## Abstract

**Background:**

The *Mycobacterium* genus encompasses at least 192 named species, many of which cause severe diseases such as tuberculosis. Non-tuberculosis mycobacteria (NTM) can also infect humans and animals. Some are of emerging concern because they show high resistance to commonly used antibiotics while others are used and evaluated in bioremediation or included in anticancer vaccines.

**Results:**

We provide the genome sequences for 114 mycobacterial type strains and together with 130 available mycobacterial genomes we generated a phylogenetic tree based on 387 core genes and supported by average nucleotide identity (ANI) data. The 244 genome sequences cover most of the species constituting the *Mycobacterium* genus. The genome sizes ranged from 3.2 to 8.1 Mb with an average of 5.7 Mb, and we identified 14 new plasmids. Moreover, mycobacterial genomes consisted of phage-like sequences ranging between 0 and 4.64% dependent on mycobacteria while the number of IS elements varied between 1 and 290. Our data also revealed that, depending on the mycobacteria, the number of tRNA and non-coding (nc) RNA genes differ and that their positions on the chromosome varied. We identified a conserved core set of 12 ncRNAs, 43 tRNAs and 18 aminoacyl-tRNA synthetases among mycobacteria.

**Conclusions:**

Phages, IS elements, tRNA and ncRNAs appear to have contributed to the evolution of the *Mycobacterium* genus where several tRNA and ncRNA genes have been horizontally transferred. On the basis of our phylogenetic analysis, we identified several isolates of unnamed species as new mycobacterial species or strains of known mycobacteria. The predicted number of coding sequences correlates with genome size while the number of tRNA, rRNA and ncRNA genes does not. Together these findings expand our insight into the evolution of the *Mycobacterium* genus and as such they establish a platform to understand mycobacterial pathogenicity, their evolution, antibiotic resistance/tolerance as well as the function and evolution of ncRNA among mycobacteria.

**Supplementary Information:**

The online version contains supplementary material available at 10.1186/s12864-022-08927-5.

## Introduction

Mycobacteria are widespread in nature and inhabit diverse niches such as water, soil, and animals (including humans). They are hardy organisms that can resist many types of stress and thrive where many other species succumb, e.g., in chlorinated drinking water. The genus *Mycobacterium* encompasses strict pathogenic species such as *Mycobacterium tuberculosis* that causes tuberculosis as well as species considered to be purely environmental, e.g., *Mycobacterium chlorophenolicum* and *Mycobacterium chubuense*. These two are being evaluated as bioremediation agents owing to their capacity to degrade various organic pollutants. Moreover, several mycobacteria cause opportunistic infections e.g., in immunocompromised hosts, and are referred to as opportunistic pathogens [1–10] and Refs therein.

While most mycobacteria are mesophilic, certain species can tolerate and grow at low or high temperatures. *Mycobacterium psychrotolerans* grows at temperatures down to +4^o^C [11], whereas several species such as *Mycobacterium hassiacum*, can grow at temperatures up to +65^o^C [12]. Mycobacteria are divided into rapid growing mycobacteria (RGM) and slow growing mycobacteria (SGM). When grown on or in standard mycobacterial media at optimal temperatures, RGM show visible growth within a week and SGM take more than 1 week. The fastest RGM grow within 1–2 days and SGM may take 20 weeks before growth is observed while *Mycobacterium leprae* has not been demonstrated to grow on synthetic media so far. The phylogenetic relationship among mycobacteria roughly follows the grouping into SGM and RGM [3, 7, 8] and Refs therein.

Until recently, mycobacterial phylogeny was based on the 16S rDNA, *hspX*, *rpoB*, or *dprE*1, either separately or in combinations. Trees based on individual genes give a good overall resolution of mycobacterial phylogeny but these trees have a limited discriminatory power. Genome sequencing has changed this and phylogenetic trees have been reported based on: (a) a core set of protein coding genes present in (almost) all mycobacteria and (b) the average nucleotide identity (ANI [13]) of their genomes have recently been reported [14–18].

Herein we provide mycobacterial genome sizes and phylogenetic trees based on 56 and 387 core genes conserved among 244 mycobacterial genomes covering the majority of species constituting the *Mycobacterium* genus. This is complemented with analysis of the ANI values for these mycobacterial genomes. The phylogeny based on the “387 core genes” was used to analyze the presence of plasmids, phages, IS elements, tRNA and aminoacyl-tRNA synthetases, and non-coding RNA (ncRNA) among mycobacteria as they related to mycobacterial clades and to growth rate and pathogenicity, and possible impact on the evolution of the genus. Our results also revealed that a core set of 12 ncRNAs, 43 tRNAs and 18 aminoacyl-tRNA synthetases are conserved among mycobacteria. Our phylogenetic analysis also identified several isolates of unnamed species as new mycobacterial species or strains of known mycobacteria.

## Results

We obtained mycobacterial type strains from the Deutsche Sammlung von Mikroorganismen und Zellkulturen in Germany and the CCUG Laboratory, Göteborg, Sweden (Table S1a). The mycobacteria were cultivated and the DNA isolated and sequenced as outlined in Methods. Genomes of 114 different mycobacteria (RGM and SGM), distributed evenly throughout the genus *Mycobacterium*, together with 130 representative genomes available at the National Center for Biotechnology Information (NCBI) were included in a comparative genomic analysis of the *Mycobacterium* genus. Among these 244 genomes 192 represent known mycobacterial species. Some of the genomes appear in duplicates since sequencing were performed by different research groups and we also included more than one strain for some to ensure species affiliation (Table S1a, which also indicates type strains and accession numbers). Most of the genomes are near-complete multi-scaffold drafts, while 47 genomes are complete single scaffold genomes. The qualities of the 244 genomes were good, with estimated completeness of more than 90%; see Supplementary information and Table S1b. All 244 genomes were grouped and analyzed based on different criteria such as growth rate and pathogenicity. For simplicity, we name the different mycobacteria as, e.g., *M. marinum* throughout the text since we mainly discuss the *Mycobacterium* genus. We have followed the historical naming of mycobacteria and clades in order to avoid confusions [3, 19–21]. Below, we present phylogenetic data and factors influencing the evolution of the *Mycobacterium* genus. Secondly, we focus on tRNA and ncRNA.

### Phylogeny and factors influencing the evolution of mycobacteria

#### Overview of mycobacterial genomes

**Fig. 1:**
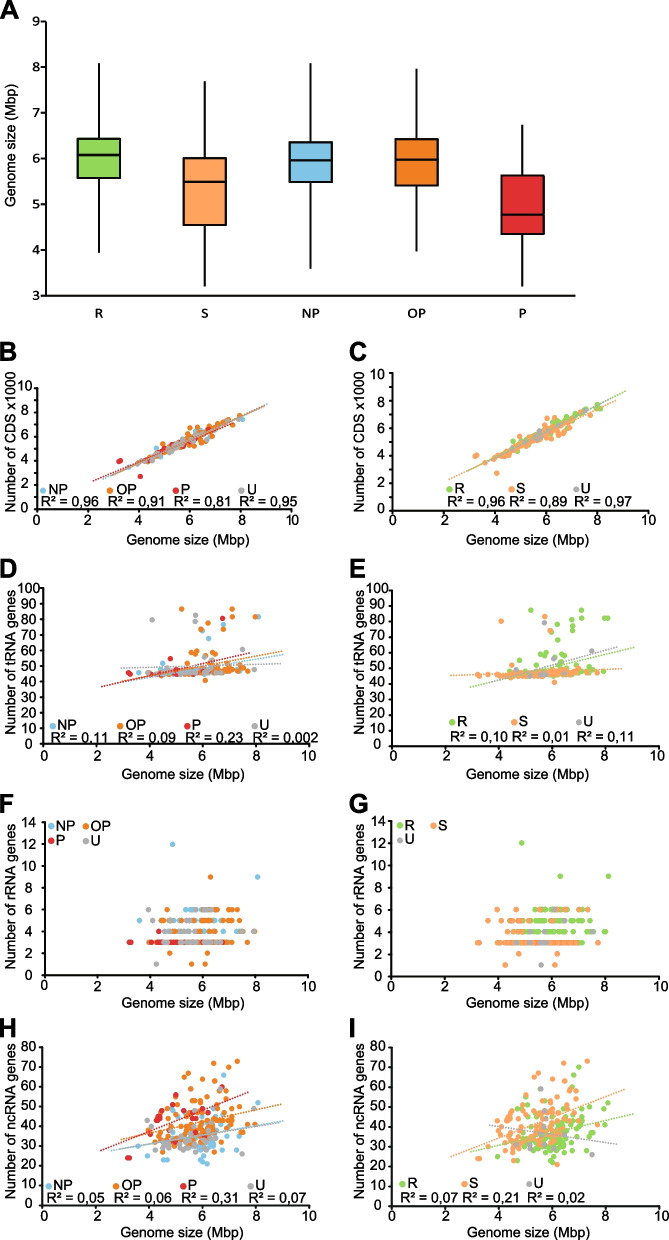
Analysis of genome features. **A** The genome size distributions were analysed based on growth rate and pathogenicity assignments and plotted as box-plots. The colored boxes indicate the extent of the second and third quartiles, while the central black line represents the median genome size. Whiskers indicate minimum and maximum genome sizes. The number of coding sequences (**B** and **C**), tRNA genes (**D** and **E**), rRNA genes (**F** and **G**), and ncRNA genes (**H** and **I**) were plotted against genome sizes and R^2^ correlations were calculated and are shown in each plot (except for rRNA). Labels are as follows: NP = non-pathogenic; OP = opportunistic pathogens; P = pathogens; U = unknown; R = RGM; S = SGM

The genome sizes of mycobacteria range from 3.2 Mb (*M. leprae*) to 8.1 Mb (*M. dioxanotrophicus*) with an average genome size of 5.7 Mb (Fig. 1a). Compared to other members of the *Corynebacteriales* order, to which mycobacteria belong [3], the average mycobacterial genome size is among the largest (Fig. S1a). On the basis of available literature [3–5, 7–10] and Refs therein we classified mycobacteria into three pathogenicity and two growth rate categories: (a) Pathogenic (P; *n* = 25, where n refers to the number of species); (b) Opportunistic pathogenic (OP; *n* = 116); (c) Non-pathogenic (NP; *n* = 66); (d) Rapid growing (RGM; *n* = 119); and (e) Slow growing (SGM; *n* = 112). For some mycobacteria we could not obtain enough information to classify them into any of the three pathogenicity categories (*n* = 37) or any of the two growth rate categories (*n* = 13).

First, we analysed the correlation between genome size and pathogenicity. As shown in Fig. 1a, the genome size of pathogenic mycobacteria is, on average, smaller (4.9 Mbp) than opportunistic/non-pathogenic mycobacteria (≈5.8 Mbp) with a *p* value of 0.0000005. Among those assigned to the pathogenic group, the smallest and largest genomes are *M. lepromatosis* (3,206,741 bp) and *M. senegalense* CK1 strain (6,738,555 bp), respectively. For the non-pathogenic and opportunistic pathogenic mycobacteria, the data suggested that the genome size distribution is similar. A comparison of SGM and RGM revealed that the average genome size for SGM to be roughly 0.5 Mbp smaller than in the case of RGM (Fig. 1a; *p* = 0.000000001). Our data further suggested that there is a linear correlation between the number of predicted coding sequences, CDS, and genome size with high R^2^-values [0.96 (NP), 0.91 (OP), 0.81 (P), 0.96 (RGM), and 0.89 (SGM); Fig. 1b, c]. In contrast, low R^2^-values [ranging from 0.01 (SGM) to 0.23 (P); Fig. 1d, e] indicated no correlation between the number of predicted tRNA genes and genome size (Fig. 1d, e). Similarly, the number of rRNA genes (Fig. 1f, g) did not correlate with the genome size nor did the number of ncRNA genes (Fig. 1h, i). Notably, while most mycobacteria carry one or two rRNA operons it appears that there are a few RGM with higher numbers of rRNA operons; *M. neworleansense* and *M. dioxanotrophicus* carry three each while *M. icosiumassiliensis* has four (Fig. 1f, g).

#### Mycobacterial phylogeny

**Fig. 2:**
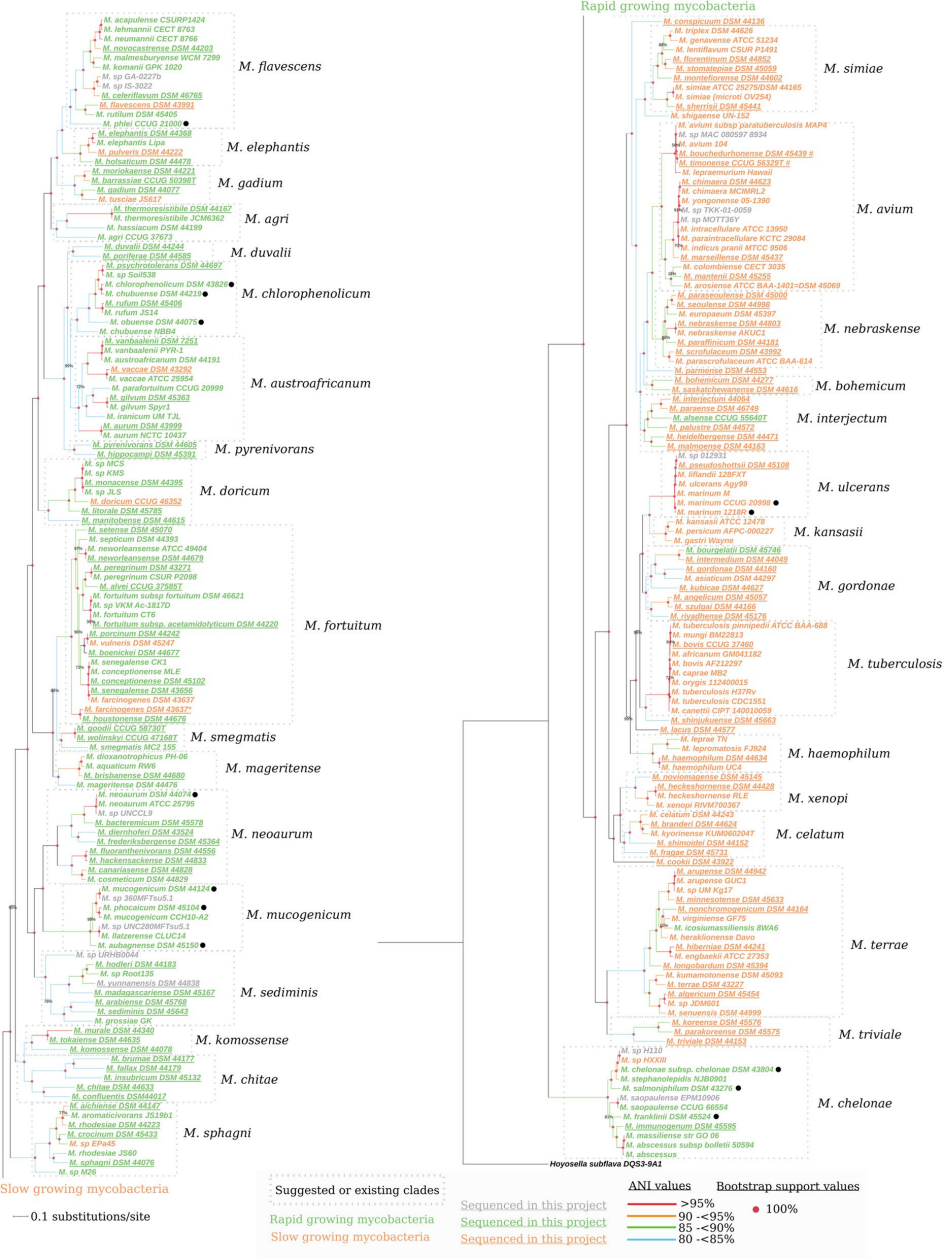
Core gene phylogeny of Mycobacteria. A phylogeny based on “387 core genes” present in all mycobacteria was calculated as described in Methods. The tree is divided into slow (SGM; orange) and rapid growing mycobacteria (RGM; green). Black indicates that no information was available to determine growth rate (“unknown”). Bootstrap support values from 1000 cycles are indicated as colored dots at the respective nodes (100%) or by their actual values (below 100%). Mycobacterial clades are indicated by boxes and vertical text to the right of the boxes refers to the clade names while species positioned outside the boxes represent single species clades. Pairwise ANI values were calculated for all the genomes; branches of the tree are colored according to these values (see legend to the left and Table S4). We emphasize to color the branches on both sides of a connecting node, all species on one side of the node must have ANI values within the range compared with all other species on the other side of the node and vice versa. Individual genomes may have ANI-values that are higher than the range of values indicated by the coloring compared with one or more genomes on the other side of the node. Underlined species were those sequenced in this study, while species marked with black dots were previously reported [6, 24–27]. *Marks the positioning of the *M. farcinogenes* DSM 43637 strain sequenced in this study while the other *M. farcinogenes* DSM 43637 strain corresponds to the available genome sequence at the NCBI database, see main text for details. ^##^Marks the isolate/genome sequence *M. microti* OV254, which based on our combined data should be considered as a *M. simiae* strain (see Discussion).

Core genes refer to those common to the mycobacterial genomes and the outgroup *Hoysella subflava* genome [22, 23]. These were identified using two methods, which are based on different homology approaches (see Methods). The PanOCT tool, which includes a bidirectional blastp approach and consideration of the gene synteny (minimum cut off of 45% identity and 60% query coverage), identified 56 hard-core protein genes, hereafter the “56 HC-genes” (see Table S2 and the Discussion). Using the SCARAP v0.3.1 tool (core pipeline setting parameters -e 245, −f 245 and -i 1, with default coverage cutoff 50%) we identified 387 orthogroups, hereafter referred to as the “387 core genes”. The “56 HC-genes” and “387 core genes” were subsequently used to construct high-resolution phylogenetic trees (Fig. 2 and Fig. S2a). The two trees are in good agreement with each other, but have some differences. Below, we focus on the “387 core gene” tree since it resulted in higher bootstrap values (Fig. 2). The “56 HC-gene” tree and the differences are discussed in the Supplementary information (Fig. S2a and b).

The “387 core gene” tree was sub-divided into 33 clades (each encompassing more than one member) and 6 single-species clades (see also Table S3a). The clade names, based on the definitions according to Goodfellow et al. [3], were retained as far as possible for historical reasons and to avoid confusion (see above). To examine the quality of the core phylogeny, all vs. all pairwise ANI values were calculated and the branches between genomes, or group of genomes in the core tree (Fig. 2; see also Tables S3b and S4, and Figs. S2 and S3), were colored according to the ANI values in five ranges: > 95% (species boundary [13, 17, 28]; in red), 90 to 95% (in orange), 85 to 90% (in green), 80 to 85% (in blue) and < 80% (in black). The ANI data supported the overall clade structures based on the “387 core gene” (as well as the “56 HC-gene”) phylogenies in which the values were highest at the “tips” of the tree and decreased with the distance from the tips towards the root (Figs. 2 and S2, see also Fig. S3 and Table S4).

#### Clade assignment of unnamed mycobacteria and identification of new species

The “387 core gene” phylogeny suggested that *M. palustre*, *M. phlei*, *M. peregrinum,* and *M. parafortuitum* clades are dissolved. The different species from these clades were relocated to other clades, forming new or single membered clades (Fig. 2; see also Table S3a for a detailed clade description). For example, *M. palustre* was relocated to the *M. interjectum* clade and *M. holsaticum* to the new (proposed) *M. elephantis* clade. Moreover, the *M. gordonae* clade was suggested to encompass *M. szulgai* as well as *M. angelicum* and *M. riyadhense*. In addition to the *M. elephantis* clade, the “387 core gene” phylogeny suggested the formation of the new clades *M. bohemicum*, *M. nebraskense*, *M. gadium*, *M. chlorophenolicum*, *M. pyrenivorans, M. mageritense*, *M. sediminis* and *M. duvalii* while *M. shigaense, M. conspicuum*, *M. parmense*, *M. shinjukuense*, *M. lacus* and *M. cookii* constitute single clades (see the Discussion).

The “387 core gene” phylogeny also allowed the identification of new mycobacterial species and subspecies which we assigned to existing or new clades. Calculation of the ANI values between all genomes in the phylogenetic tree supported the suggestion to consider these mycobacteria as new species using ANI = 95% as “species threshold value” [17, 28] (Figs. 2, S2 and S3; Tables S3a, b and S4; and Fig. S3b-k compare the ANI values for these unnamed species with the respective clade members). Two unnamed species were assigned to the *M. sphagni* clade (Fig. S3b): *M.* sp. M26, which is deeply rooted in the clade, and *M.* sp. Epa45, which is close to but different from, *M. crocinum*, and *M. rhodesiae* JS60. The *M. rhodesiae* JS60 strain is separated from the *M. rhodesiae* type strain (DSM 44223) and phylogenetically closer to *M. sphagni* and therefore suggested not to be a *M. rhodesiae* species in keeping with a recent report [15]. Moreover, two unnamed mycobacteria belong to the *M. flavescens* clade (Fig. S3c), *M.* sp. GA-0227b and *M.* sp. IS-3022, and are clearly separated from each other as well as from the closely related species, *M. celeriflavum*. Finally, *M.* sp. URHB004 see also [24], which is deeply rooted in the *M. sediminis* clade, and *M.* sp. Root135 within the same clade are well separated from any other species (Fig. S3d). Taken together, we suggest that these unnamed mycobacteria should be considered as new species. This is also supported by the “56 HC-gene” tree (Fig. S2b).

For the other unnamed mycobacteria, the branch depth was smaller. On the basis of species and subspecies thresholds 95 and 98% [17, 28], respectively, our “387 core gene” (and “56 HC-gene”) based phylogeny (Fig. 2) and ANI analysis suggested the following. The isolates *M*. sp. TKK-01-0059 and *M*. sp. MOTT36Y assigned to the *M. avium* clade should probably be considered to be *M. yongonense* strains, whereas *M*. sp. MAC 080597 8934 is closely related to *M. bouchedurhonense*, *M. timonense* and *M. avium* 104 (Fig. S3e).

Two isolates were assigned to the *M. terrae* clade where *M*. sp. UM Kg17 belongs to the *M. arupense* group, and *M*. sp. JDM601 should be considered a *M. algericum* subspecies (Fig. S3f). The lone isolate, *M*. sp. 012931, belonging to the *M. ulcerans* clade, is closest to *M. pseudoshottsii* or *M. liflandii*; however, we emphasize that all mycobacteria in this clade show ANI values above the subspecies threshold (Fig. S3g; see also Ref [25]). The two unnamed mycobacteria assigned to the *M. chelonae* clade likely represent a single *M. chelonae* subspecies (Fig. S3h, ANI values for members of the *M. chelonae* clade; see also Ref [26]). Three isolates, *M.* sp. KMS, *M*. sp. MCS and *M*. sp. JLS, were assigned to the *M. doricum* clade and our data suggested that they should be considered as *M. monacense* strains (Fig. S3i). Finally, *M*. sp. UNNCL9 belonging to the *M. neoaurum* clade is suggested to be a *M. neoaurum* strain (Fig. S3j) and the *M. fortuitum* clade member *M*. sp. VKM Ac-1817D strain a *M. fortuitum* strain (Fig. S3k).

To conclude, the “387 core gene” (and “56 HC-gene”) phylogenetic trees, together with the ANI data, provided insight into i) the organization of the clades constituting the *Mycobacterium* genus, ii) clade allocation, and iii) proximity of the phylogenetic relationships for unnamed mycobacterial isolates. For the complete list of newly assigned or re-assigned species and subspecies see Table S3a, b.

#### Presence of plasmids

To identify plasmid sequences, we assembled raw reads (Illumina and Ion Torrent data) using “plasmidSPAdes” (see Methods). Following this we identified plasmids in 20 mycobacteria, 6 SGM and 14 RGM (Table S5a, b). Six of the 20 plasmids have previously been detected in other mycobacteria and 14 were new plasmids previously unreported. One of these latter, pJCM15653 was present in *M. boenickei* and partial hits were detected in *M. peregrinum* and *M. septicum* (not shown); all three belong to the *M. fortuitum* clade. Of the four *M. gadium* clade members, three harbor different plasmids where *M. gadium* carries pMM23, a plasmid which previously was reported to be present in the *M. marinum* M strain [29].

Prediction of the plasmid genes revealed many hypothetical genes and a number of interesting homologs (for annotation, see Table S5c). These homologs include di-guanylate cyclase *dosC* (*M. crocinum*; di-guanylate cyclase participates in the synthesis of the signal molecule c-di-GMP [30]); transcriptional factor *whiB*6; anti-sigma factor F antagonist *rsfA* (*M. gordonae*); *dnaA* (*M. chimaera*); and house-keeping sigma factor *sigA* (*M. komossense* and *M. moriokaense*). Plasmids in the *M. ulcerans* and *M. chlorophenolicum* clade members are found in Refs [6, 25].

Searching for plasmids in 197 draft genomes (excluding the complete genomes) using the PLSDB database resulted in 30 known plasmid sequences, corresponding to 29 circular plasmids and one linear plasmid, the latter present in *M. branderi* (Table S5b). For *M. chimaera*, *M. nebraskense* and *M. parascrofulacuem* we detected the presence of multiple known plasmid sequences; regarding *M. chimaera* DSM 44623, see also Ref [31].

Taken together, our analysis revealed the presence of 14 new plasmid sequences: 10 in RGM and 4 in SGM. Moreover, it appears that certain mycobacterial strains, such as *M. chimaera* and *M. nebraskense*, have been exposed to different plasmids that might have affected their evolution.

#### Presence of phages

**Fig. 3:**
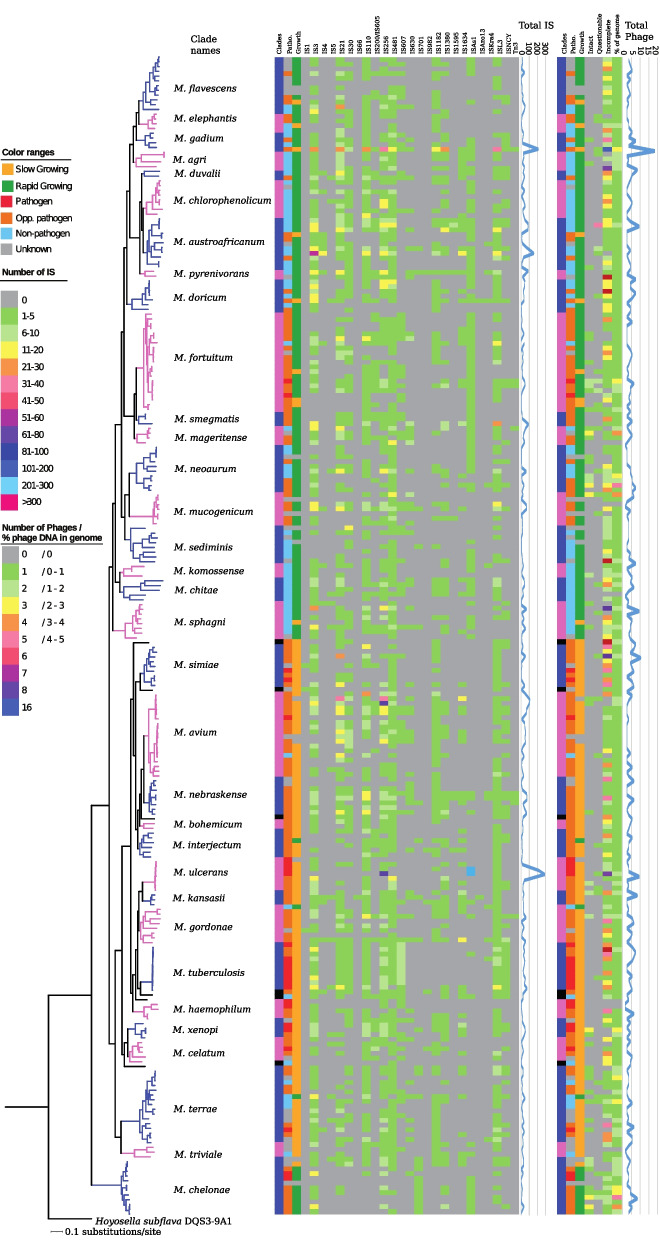
Distribution of IS elements and Phages in mycobacteria. Heat maps for 244 mycobacteria showing the presence of insertion sequence (IS) elements and bacteriophage derived sequences predicted using ISsaga [32] and Phaster [33], respectively. The “387 core gene” phylogenetic tree (see Fig. 2) and clade names are shown to the left (the branches and clades column are marked in pink and blue, alternating, while black mark single clades to facilitate guidance). The second and third columns indicate pathogenicity and growth rate, respectively, according to the colour key. The types of IS elements, and classification of predicted phage derived sequences are as indicated on the top. The different colors represent the numbers of IS elements and percentage of phage DNA per genome (see color key to the left in figure). Plots of the total number of predicted IS elements and percentage of phage DNA per genome are shown to the right of the respective heat maps

Phages contribute to the diversity of genomes and play a role in horizontal gene transfer, HGT. We therefore predicted the presence and impact of phage genomes/sequences in mycobacteria as the percentage of the genome sequences with similarity to phage-derived genes. We predicted intact phage genomes to be present for 46 mycobacteria (Fig. 3; Table S6). Most (30 mycobacteria) carried one phage each, but up to three phages were detected in six species (*M. aquaticum* strain RW6, *M. canariasense* DSM 44828, *M. mucogenicum* DSM 44124, *M. heckeshornense* RLE, *M. immunogenum* DSM 45595, and *M. abscessus* subsp. *bolletii* 50,594). Including the questionable and incomplete phages, 238 genomes carried phage-derived genes. For 6 mycobacteria, we did not detect any phage-derived sequences, e.g., *M. leprae* and *M. lepromatosis* in the *M. haemophilum* clade. On average, 0.7% (range 0 to 4.64%) of the mycobacterial genomes consisted of phage-like genes (Table S6). For the majority of the mycobacteria (231 genomes) the phage content deviated less than two standard deviations (±1.38%; calculated for the whole *Mycobacterium* genus, i.e., 2 × 0.69%; see Table S6) from the average number 0.7%. Among the 13 mycobacteria with higher phage content than average, 9 belonged to the RGM with *M. immunogenum* having the highest fraction (4.64% of the total number of genes). There was no statistically significant difference between SGM and RGM, nor between the NP, OP, and P categories. At the clade level, *M. mageritense* and *M. chelonae* clade members were predicted to have significantly higher phage content (1.49 and 1.75%, respectively) than the average mycobacteria (Table S6). Thus, it appears that phages have had a larger impact on the evolution of these species compared to the majority of mycobacteria. With respect to the possible link between phage and tRNA see below and the Discussion.

#### Identification of IS elements

IS-elements also represent an important driving force for the evolution of bacterial genomes; they can disrupt genes and influence transcription/expression of genes close to the integrated IS element [34]. Hence, we predicted different types and numbers of IS elements in mycobacteria (Fig. 3). At least one IS element was detected in all mycobacteria but none was universally present in mycobacteria. The average number of IS elements in the genus was 28.3 copies with a standard deviation of 32.7 (Table S7a) indicating a high variability. (Due to draft genome status for many of the genomes, we emphasize the difficulties in accurately determining the number of IS elements.) The most common family of IS elements, found in > 65% of the genomes, included IS*3*, IS*110*, IS*256*, IS*481*, and ISL*3* (Fig. 3). Of these, IS*3* and IS*256* are present with the highest average copy number per genome (5.5 and 5.8, respectively; Fig. 3 and not shown) albeit the variation in copy numbers among mycobacteria is high (ranging between 1 and 53 and 1 and 80, respectively). The IS*As1* type is present with the highest number (212 copies) in any individual genome (*M. liflandii*). *M. ulcerans* and *M. liflandii* have the highest total number of IS elements, 290 and 218, respectively (Table S7b). In contrast, only one IS element was found in *M. cookii* (IS*481*) while two were predicted in *M. salmoniphilum, M. saopaulense* EPM10906 (one IS*As1* and one IS*701*), and *M. chelonae* (one IS*481* and one IS*701*); all of these belong to the earliest diverging mycobacterial lineage.

With respect to growth rate and pathogenicity classifications (RGM, SGM, NP, OP and P), the mycobacteria pathogens had significantly more IS-elements than the opportunistic ones (45.1 vs 28.0; *p* < 0.04; Table S7a). Variation within groups was high for all categories. Among species that we could classify with respect to growth rate and pathogenicity, IS*66* and IS*Azo13* were only found in slow-growing opportunistic pathogens (in one and two species, respectively) while IS*1595* was only detected in four opportunistic and four non-pathogens (Fig. 3; Table S7b). IS*1* was only found in SGM of unknown pathogenicity (three species). At the clade level, the *M. ulcerans*, and *M. gadium* clades harbor a significantly higher than average number of IS elements (88.0 and 69.8, respectively). This indicates that IS elements have played a significant role in the diversification of the species in these clades.

Our comparison of IS element types in mycobacteria detected no clear correlation with respect to clades, pathogenicity, or growth rate, with a few notable exceptions. Within the *M. tuberculosis* and the *M. chelonae* clades, there was a high degree of correlation, with species in each clade having similar sets of IS element types (Figs. 3 and S4, and Supplementary information). This suggests a low rate of gain of new IS elements and a low rate of loss of existing ones within these clades compared the to other mycobacteria.

Taken together, the data emphasizes the possible impact of IS elements on the evolution of the *Mycobacterium* genus (see also the Discussion).

### Comparison of the presence of tRNA and ncRNA among mycobacteria

tRNA and non-coding RNA have key roles in the expression of genes and their regulation. tRNA genes also act as targets in integrating foreign DNA, leading to the establishment of e.g., pathogenicity, and metabolic and resistance islands [35, 36] and Refs therein. Among bacteria, including some mycobacteria, tRNA genes have been horizontally transferred by phages [24]. Together this indicates that tRNA and ncRNA have indeed contributed to bacterial evolution. Hence, we mapped the presence of tRNA and ncRNA genes among mycobacteria (see Methods). Since aminoacyl-tRNA synthetases (AARS) are closely linked to tRNA we also surveyed for the presence of AARS genes. Below we will discuss tRNA and AARS, then identified ncRNAs.

#### Variation of the number of tRNA genes among mycobacteria

**Fig. 4:**
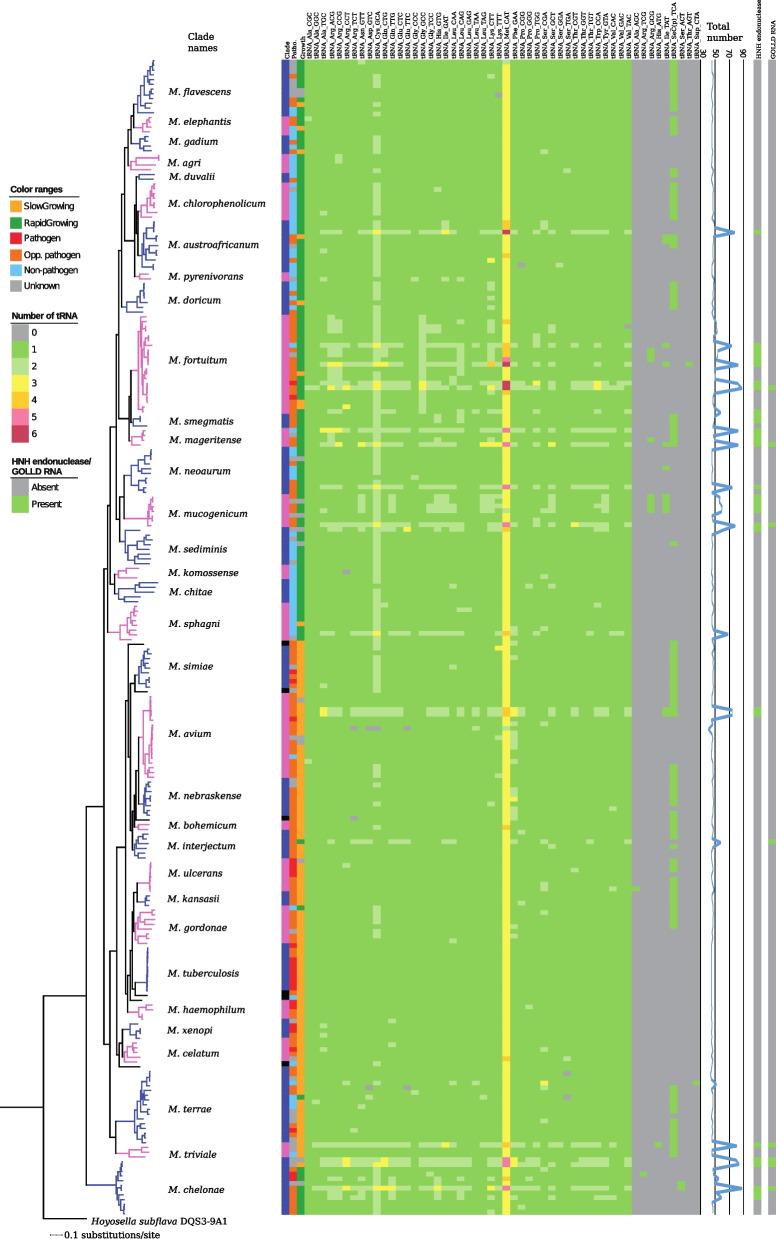
Distribution of tRNAs in mycobacteria. Heat map showing the presence of tRNAs for 244 mycobacteria. The “387 core gene” phylogenetic tree (see Fig. 2) and clade names are shown to the left and the colouring scheme is the same as in Fig. 3. Core tRNAs are present in most of the mycobacteria, while auxiliary tRNAs are present in a minority. The presence or absence of tRNA isoacceptors are marked in green and gray, respectively. The total number of predicted tRNAs is shown as indicated. To the right we show the presence (green) and absence (gray) of the HNH endonuclease and the GOLLD ncRNA genes; for details see main text and Supplementary information

Except for tRNA^Met^(CAT) and tRNA^Cys^(GCA), which are present in multiple copies in most mycobacteria, the remaining tRNA isoacceptors are generally present as single-gene copies in the mycobacterial genomes (Fig. 4). On average, mycobacteria are equipped with 49 tRNAs genes (Table S8a, b; range 41–87). Certain mycobacteria, however, have higher numbers and 16 mycobacteria carry more than 17 tRNA genes higher than the average number (including multiple gene copies of several tRNA isoacceptors). These mycobacteria belong to RGM and some harbor a large fraction of phage derived genes in their genomes (see above and the Discussion). Members of four clades encoded significantly more tRNA genes than average, with *M. mageritense* clade members carrying the highest numbers (65.8; *p* = 0.000183). Others belong to the *M. fortuitum*, *M. mucogenicum* and *M. chelonae* clades [24, 26]. The genomic locations of tRNA genes in 47 mycobacteria (for which complete genomes are available) were compared with *M. chelonae* as reference, which belongs to the earliest mycobacterial lineage (Figs. 2 and S2a). Our comparison revealed differences in the chromosomal locations of the tRNA genes. However, we also noted similarities when comparing *M. chelonae* and *M. avium* clade members (Fig. S5a). Interestingly, the positioning of tRNA genes [tRNA^Ile^(GAT), tRNA^Ala^(TGC) and tRNA^Leu^(CAG)] close to *dnaA* and origin of replication, *oriC* see also [24, 27], appears to be conserved among these mycobacteria. The tRNA^Ile^(GAT) and tRNA^Ala^(TGC) are likely to be transcribed together and they are among the tRNAs identified to be necessary for optimal growth of *M. tuberculosis* H37Rv [37].

**Fig. 5:**
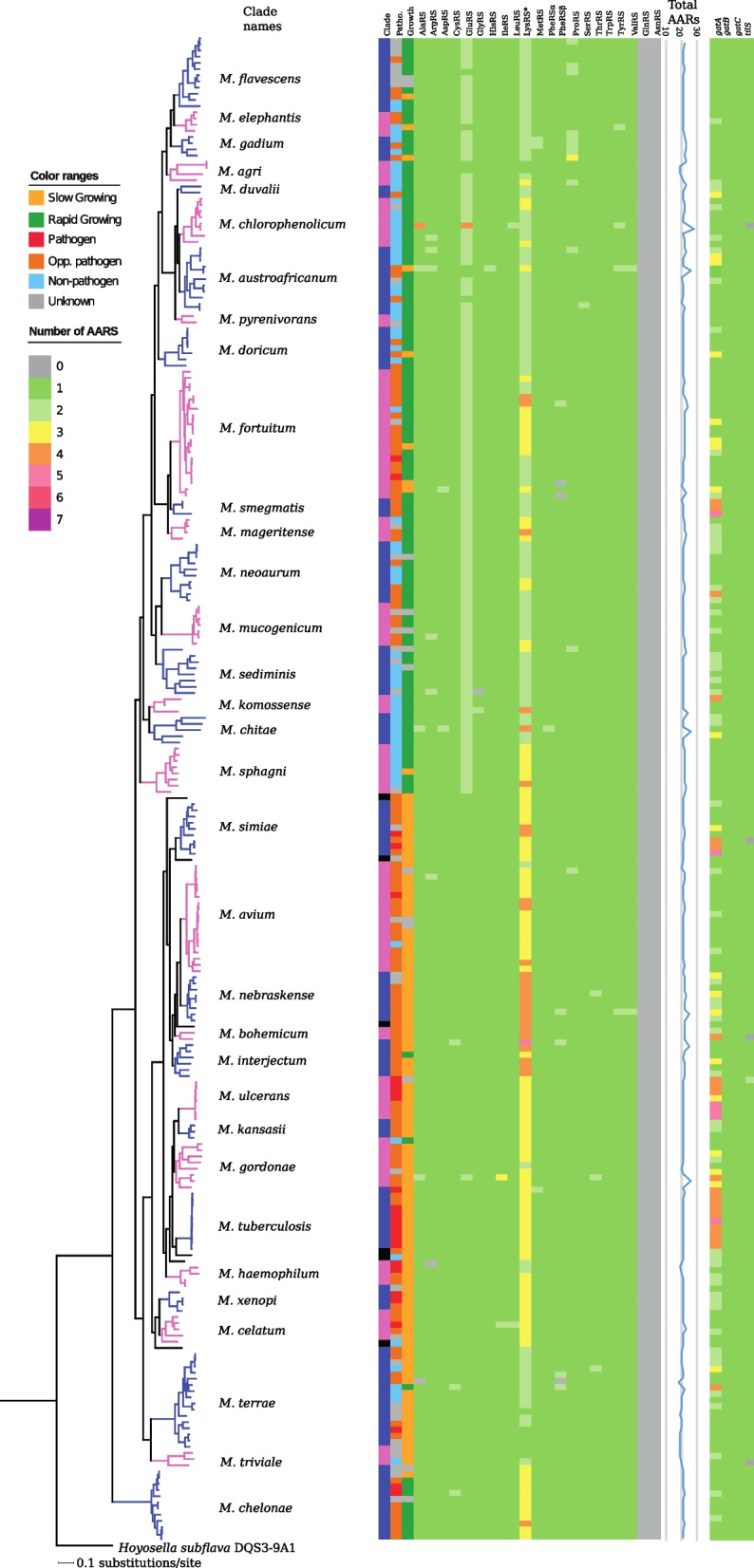
Distribution of aminoacyl-tRNA synthetases in mycobacteria. Heat map showing the presence of aminoacyl-tRNA synthetases (AARS), *gatABC* and *tilS* in 244 mycobacteria. The “387 core gene” phylogenetic tree (see Fig. 2) and clade names are shown to the left and the coloring scheme is the same as in Fig. 3. The total number of predicted AARS is shown to the right of the heat map. * indicates that LysRS includes both the regular LysRS and the lysyl-phosphatidyl-glycerol biosynthesis bifunctional protein LysX (see Supplementary information)

Among mycobacteria, the tRNA isoacceptor genes, tRNA^Ala^(AGC), tRNA^Arg^(TCG), tRNA^Arg^(GCG), tRNA^His^(ATG), tRNA^Ile^(TAT), tRNA^Ser^(ACT) and tRNA^Thr^(AGT) are rare (Fig. 5; Table S8a). The tRNA^Ala^(AGC), tRNA^Arg^(GCG), tRNA^His^(ATG) and tRNA^Thr^(AGT) genes were only detected as single copies in *M. kansasii*^atcc12478^, *M. salmoniphilum*^dsm43276^, *M. parakoreense*^DSM45576^ and *M. fortuitum*^dsm44220^, respectively. All mycobacteria carried tRNA^Ile^(CAT), which allows reading of AUA as a result of modifying the C in the anticodon to 2-lysyl-cytidine. This modification is catalyzed by TilS [38], and *tilS* homologs were predicted in almost all mycobacteria. (When no homolog could be identified, was likely due to draft genome status; Fig. 5 and Table S8b). For 12 mycobacteria, the rare tRNA^Ile^(TAT) was predicted to be present, in addition to tRNA^Ile^(CAT). Hence, there are two ways to read AUA in these mycobacteria see also [24]. We also predicted one UAG tRNA suppressor gene in *M. minnesotense*^DSM45633^, as well as the presence of a selenocysteine tRNA^SeC^(TCA) gene in 103 mycobacteria (Fig. 4; Table S8b).

Taken together, our data suggest a high variability in the number of tRNA genes among mycobacteria (see the Discussion).

#### Mycobacteria lack AsnRS and GlnRS genes

Aminoacyl-tRNA synthetases (AARS) are responsible for attaching the correct amino acid to the respective tRNA. As previous studies indicate [24], all AARS were predicted to be present in all mycobacteria with the exception of AsnRS and GlnRS (Fig. 5; Table S9). In the absence of AsnRS and GlnRS charging is accommodated through the tRNA-dependent amidotransferase pathway (Adt) where the GatCAB enzymes are essential [39]. Indeed, *gatCAB* homologs were universally present among mycobacteria (Fig. 5; Table S9a) suggesting that the Adt pathway operates and is a common characteristic for the *Mycobacterium* genus. For prediction of aminoacyl-tRNA-synthetase paralogs see Supplementary information.

#### Variation of non-coding RNA genes among mycobacteria

**Fig. 6:**
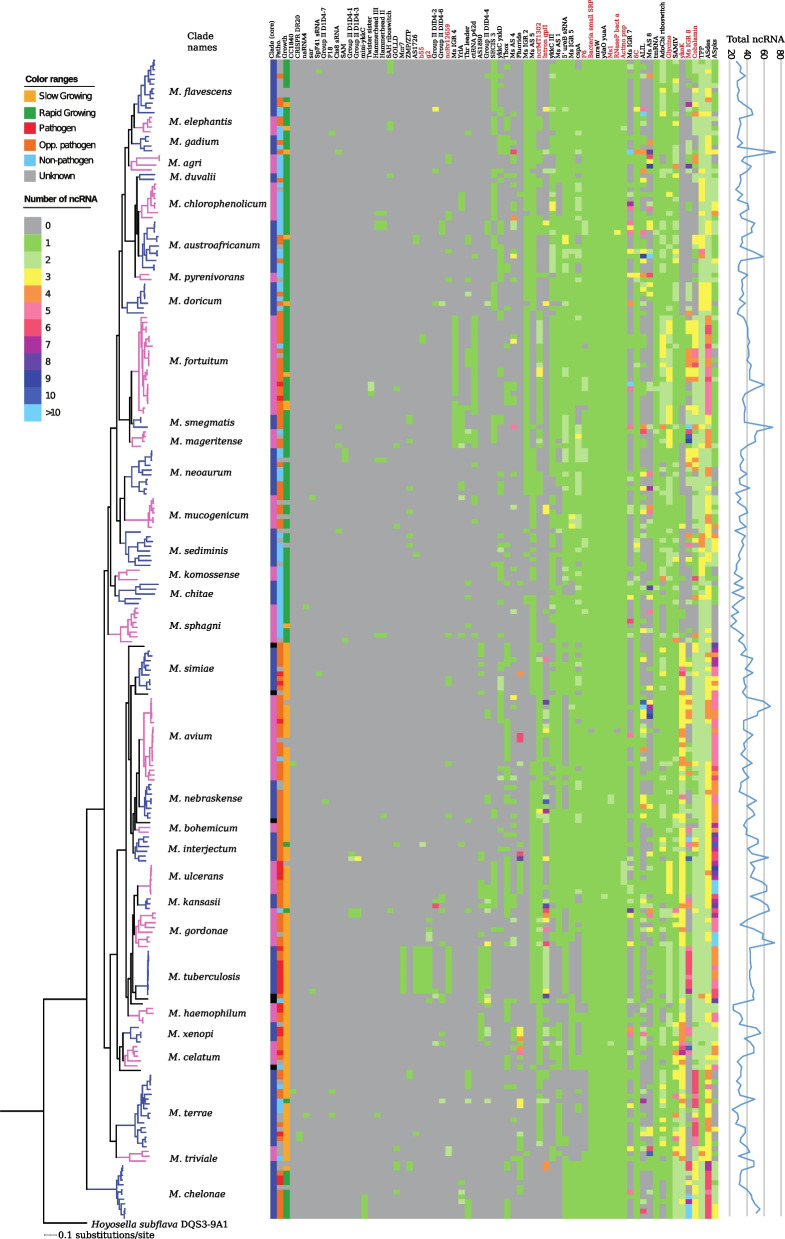
Distribution of non-coding RNAs in mycobacteria. Heat map showing the presence of non-coding RNAs in 244 mycobacteria predicted using the RFAM v13.0 database [41–43] and INFERNAL v1.1.2 [44]. The “387 core gene” phylogenetic tree (see Fig. 2) and clade names are shown to the left and the coloring scheme is the same as in Fig. 3. The presence and number of non-coding RNAs is indicated according to the color legend. The ncRNAs marked in red correspond to ncRNAs also predicted using the “*M. tuberculosis* H37Rv ncRNA data set” [40] (see Supplementary Fig. S6). A plot of the total number of predicted non-coding RNAs is shown to the right

To provide insight into the presence and role of non-coding RNAs (ncRNAs) among mycobacteria, we used the Rfam database (see Methods). In addition, we searched for ncRNA homologous to those of *M. tuberculosis* ncRNAs [40] (Figs. 6 and S6). The average mycobacterial genome encodes 39.2 ncRNAs of which 12 ncRNAs were predicted to be universally present among mycobacteria. The *M. gordonae* (53.6; *p* = 0.000053), *M. kansasii* (54.3; *p* = 0.0074), *M. ulcerans* (52.6; *p* = 0.00035) and *M. interjectum* (49.5; *p* = 0.0102) clades were predicted to have significantly more ncRNA genes than the genus average number and other clade members to have fewer (Fig. 6; Table S10a); the *M. sphagni* (27.3; *p* = 0.00061) and *M. haemophilum* (28.8; *p* = 0.033) clades both have < 30 detected ncRNA genes (with *M. leprae* having 24). Pathogens and opportunistic pathogens have significantly more (42.4; *p* = 0.00090; and 42.2, *p* = 5.79 × 10^− 6^, respectively) ncRNAs than non-pathogens (35.4), and SGM have significantly higher number of ncRNA genes (41.5; *p* = 0.00062) than RGM (37.2). The highest number of ncRNAs predicted was 73 in the SGM *M. tusciae* (Table S10).

Among core ncRNAs, ribonuclease P RNA (RPR; *rnpB* [45]), transfer-messenger RNA (tmRNA; *ssrA* [46]), signal recognition particle RNA (4.5S RNA), Ms1 RNA and 6C RNA were identified. (Notably, 6C RNA was not detected in *M. leprae* or *M. lepromatosis*). Moreover, we noted the presence of an anti-sense RNA targeting the fatty acid desaturase, *des*A2 (ASdes) [47] among core ncRNAs, as well as several cis-regulatory riboswitches [48] including *mraW* (putative regulator of peptidoglycan synthesis), *ydaO-yuaA* (which binds the signal molecule cyclic di-AMP; see below) and Actino-*pnp* (located in the 5′ UTR, untranslated region, of *pnp*, polynucleotide phosphorylase). These core ncRNA genes were present in single copies in all mycobacteria with few exceptions as discussed below. For some mycobacteria we detected multiple copies for other universally conserved riboswitches: the Glycine riboswitch (regulates glycine degradation in response to glycine) and SAMIV (responsive to the concentration of S-adenosyl methionine). Most have more than one copy of the TPP riboswitch (responsive to the level of thiamine pyrophosphate, i.e., the active form of vitamin B1 [49]). Together, this indicates the importance of regulating these targets/processes in mycobacteria.

Several other identified ncRNAs were confined to a (usually small) subset of mycobacteria, e.g., the horizontally transferred GOLLD RNA [24, 50] (see the Discussion). The *M. tuberculosis* ncRNAs predicted by Wang et al. [40] were also detected in some mycobacteria outside of the *M. tuberculosis* complex (Fig. S6; note that some of these ncRNAs overlaps with the Rfam annotated ncRNAs).

To summarize, a core set of 12 ncRNAs are present in almost all (> 99%) mycobacteria. Another set of five ncRNAs are present in many (> 83%), while eight additional ncRNAs are present in 54–75% of the mycobacterial genomes. Together, this provides information about conserved ncRNA that are involved in the growth, survival and stress tolerance of mycobacteria. We also noted that mapping the core ncRNA genes on the 47 mycobacterial (complete) genomes - using *M. chelonae* as reference - suggested differences in their locations, which was in keeping with what we observed for tRNA genes (Fig. S5b; see above).

Interestingly, for some mycobacteria more than one gene copy of RPR, tmRNA, Ms1 RNA, 6C RNA, *ydaO-yuaA* riboswitch and the ALIL RNA pseudoknot was annotated. Three RPR homologs were annotated in *M. austroafricanum* and *M.* sp. YC-RL4 [51] (Fig. 6; Table S10b). One of the identified RPR homologs folds into a regular bacterial RPR structure. For the two additional copies, the overall similarity was low for MAUSTDSM44191_20 while deeper analysis of the second copy, MAUSTDSM44191_22, revealed structural differences, in particular in the specificity domain [45] and Refs therein. This raises questions about the function of the two extra RPRs in these two mycobacteria albeit conserved residues important for RPR function are present [45] (Fig. S7). In this context, the gene positioned between the extra annotated RPR genes is annotated to encode an endonuclease, which might indicate that the sequences encompassing these two additional copies have been acquired through HGT.

Four mycobacteria, *M. austroafricanum*, *M. canariasense*, *M. conceptionense* and *M. dioxanotrophicus*, harbor extra homologs of tmRNA. Analysis of the extra tmRNA genes revealed that these possibly have been acquired through horizontal gene transfer. For *M. austroafricanum* and *M. canariasense* our data suggested that their extra tmRNA gene originated from a *Mycobacterium* phage, Nappy, while in the case of *M. conceptionense* and *M. dioxanotrophicus* the origin appeared to be *Tsukamurella tyrosinosolvens* strain PH-06 (Fig. S8)*.* In keeping with that these have been horizontally transferred is that translation of these extra tmRNAs would result in proteolysis tags different from the tag generated by the regular mycobacterial tmRNA (Fig. S8). It remains to be seen whether these extra tmRNAs are functional and, if so, how they affect the distribution of polypeptides carrying different tags and the subsequent degradation of these polypeptides (see the Discussion).

Three mycobacteria, *M. nebraskense* (two strains; see also Ref [24]) and *M. celatum*, carry an additional copy of Ms1 RNA, which is suggested to function as a mycobacterial 6S RNA variant [24, 52, 53] (Fig. S9; 6S RNA in e.g. *Escherichia coli* is involved in the regulation of stationary phase genes [54]).

For the *Actinobacteria* specific 6C RNA, we identified more than one copy in several mycobacteria belonging to the *M. flavescens*, *M. gadium* and *M. sediminis* clades where *M. tusciae* strain JS617 and *M. yunnanensis* strain DSM 44838 carry three extra 6C RNA genes (Figs. 6 and S10; Table S10). The secondary structures (not shown) of these extra 6C RNAs are similar to the regular 6C RNA [24] and the genes were annotated to be positioned on the chromosomes as well as on plasmids (Fig. S10). Interestingly, in mycobacteria the regular 6C RNA gene is closely linked to the Ms1 RNA gene (generally one gene in between; Fig. S10) but this is not the case for the extra 6C RNA genes.

We also predicted the presence of an extra *ydaO-yuaA* riboswitch upstream of the hypothetical gene MKAN_24085 in *M. kansasii*. This riboswitch binds the signal molecule c-di-AMP and thereby influences regulation of *rpfA* (resuscitation-promoting factor A [48]). No extra copy is present in neighboring species such as *M. persicum* and *M. gastri*. Hence, the extra *ydaO-yuaA* has been acquired after *M. kansasii* diverged from *M. persicum* and *M. gastri* (Figs. 2 and S11).

In bacteria, translational frameshifting is induced by the ALIL RNA pseudoknot motif and it was originally identified in association with transposable elements belonging to the IS*3* family. The frameshifting event results in a polypeptide having both transposase and integrase core domains, which eventually leads to transposition [46, 55]. For 113 of the mycobacterial genomes, we predicted the presence of ALIL motifs with the gene synteny “Transposase – ALIL – Integrase Core Domain” in 75 of these mycobacteria while a different gene synteny was observed in 17 genomes (with ALIL not “in between” but at one end or with completely different gene synteny; Fig. 6 and not shown). We did not detect any ALIL motif in 21 of the selected 113 genomes, which is likely due to their draft genome status. Moreover, the number of ALIL RNA motifs varies ranging between zero and twenty: *M. avium* strain 104 has twenty and the *M. phlei* strain CCUG21000 has twelve. A comparison of the complete and draft (Illumina generated; unpublished) *M. phlei*^ccug21000^ genomes predicted only one ALIL motif was predicted for the draft genome. This supports the notion that we did not detect any ALIL motifs in 21 draft genomes and might indicate that ALIL motifs in mycobacteria are underestimated when only draft genomes are available (see above). Nevertheless, taken together this indicates the importance of ALIL RNA elements among mycobacteria and the possible impact on the diversification of some mycobacteria (with respect to IS elements see above).

## Discussion

The *Mycobacterium* genus belongs to the family *Corynebacteriales* [3, 56] and the sizes of the genomes for mycobacteria range between 3.2 to 8.1 Mbp with an average size of 5.7 Mbp. Genome phylogeny presented here and by others [14–17] suggest that *M. chelonae* clade is the earliest mycobacterial lineage, and members of this clade are phylogenetically close to *H. subflava* and *Segniliparus* spp. (see also Supplementary information and Fig. S12). Here we used *H. subflava* as an outgroup and generated phylogenetic trees based on 56 and 387 core genes present among 244 mycobacterial genomes. These phylogeny-based trees were analyzed for factors that might have an impact on the evolution of the genus.

### Factors contributing to the evolution of mycobacteria

The numbers and types of IS elements, and the presence of phage sequences and plasmids vary among mycobacteria. These and other factors all possibly contribute to the evolution of the genus and bacteria in general (see also Refs [6, 24, 26, 27, 57]), e.g., the presence of HNH endonucleases, transposases, RNA elements such as ALIL pseudoknots (required to induce transposition [46, 55]), presence/absence of ncRNAs, numbers of tRNA genes, and horizontally transferred genes all possibly contribute to the evolution of the genus and bacteria in general.

It has been suggested that among mycobacteria, members of the *M. ulcerans* clade constitute a group to use for identifying and understand bacterial evolution [25, 58]. Interestingly, the mycobacteria belonging to this clade carry high numbers of IS elements and phage sequences suggesting their impact on the evolution of *M. ulcerans* clade members. On the basis of our current phylogeny, it would be of interest to include other clusters of mycobacteria for addressing questions related to bacterial evolution, e.g., the members of the *M. avium*, *M. gadium*, *M. austroafricanum* and *M. chelonae* clades (the three latter being RGM). Moreover, *M. chelonae* clade (the earliest mycobacterial lineage) members contain fewer numbers of IS elements than most mycobacteria. Low numbers are also observed for *H. subflava* and *S. rotundus* (Table S11; see also Ref [26]). Thus, the expansion of the number of IS elements and types occurred after the split of the *M. chelonae* clade members from the other mycobacteria.

It is again worth noting that insertion of IS elements could disrupt genes and also influence the expression of genes close to the insertion site. As such, this could be one factor that would drive the evolution of mycobacteria. For example, in other bacteria, IS elements are reported to affect virulence, antibiotic resistance and metabolism [34]. This warrants further studies to understand factors influencing the expansion of the number and class of IS elements. In this context, we also emphasize the variations in the genomic positions of both tRNA and ncRNA genes among selected mycobacteria (Fig. S5) and as such they are suggested to be indicators of chromosomal rearrangements during the evolution of the *Mycobacterium* genus.

### Genome based mycobacteria phylogenies - a comparison

We constructed two phylogenetic trees based on 56 and 387 core genes present in 244 mycobacterial genomes [6, 24–27]. Our ANI analysis supported both these phylogenetic trees and the differences between the two trees are discussed in Supplementary information. Here we focus on the “387 core gene” tree. Trees based on i) ANI (referred to as the “Tortoli ANI” tree; note that Fedrizzi et al. [14] also identified 243 genes present in 99 mycobacteria, see Supplementary information) [14, 15, 17], ii) 1941 soft-core genes (the “Gupta core gene” tree) [16], and iii) 288 hard-core genes (and 1306 “soft-core” genes) present in 175 mycobacteria (the “Matsumoto hard- and soft-core gene” trees) [18] have recently been reported (see also Ref [21] and Supplementary information where we compare how we and others identified core genes).

Overall these and our genome-based phylogenies are in good agreement with each other while differing from the traditional 16S rDNA phylogenetic tree [3]. Interestingly, our trees and the previously reported genome-based trees show a high level of agreement for SGM with few differences in clade “membership”, in particular with respect to the “Tortoli ANI” and “Gupta core gene” trees (Fig. S13, see also Fig. S2b). The “Gupta core gene” tree [16] (based on 1941 core genes) grouped *M. malmoense* in the *M. nebraskense* clade, whereas in our trees and the “Tortoli ANI” tree *M. malmoense* is positioned in the *M. interjectum* clade (Fig. S13). We and the Tortoli laboratory used the type strain *M. malmoense* DSM 44163, while Gupta et al. used *M. malmoense* E614 [16]. Hence, this discrepancy can be attributed to the use of sequences from different strains in these studies. Moreover, the “Tortoli ANI” tree [17] group *M. szulgai* and *M. angelicum* close to *M. riyadhense* and distant from the *M. gordonae* clade members. In contrast, the “387 core gene” tree positioned these three mycobacteria in the *M. gordonae* clade (see also Fig. S3l showing ANI values for *M. gordonae* clade members). The “387 core gene” tree further suggested that the SGM *M. vulneris* DSM 45247 belongs to the *M. fortuitum* clade, which mainly encompasses RGM. The ANI value = 99.97% (Table S4 and Fig. S3k) comparing *M. vulneris* DSM 45247 and *M. porcinum* the ANI value = 99.97%. The “Tortoli ANI” [17] and the “Matsumoto soft-core gene” trees [18] group *M. vulneris* together with the SGM *M. avium* clade members. We therefore conclude the *M. vulneris* DSM 45247 isolate, sequenced in this study, should be considered as a *M. porcinum* strain (see also Refs [15, 59]). We also noted that in the “Matsumoto soft-core gene” tree the SGM *M. koreense* and *M. triviale* are located among RGM in the *M. chitae* clade. This is in contrast to our trees, as well as the “Tortoli ANI” and the “Gupta core gene” trees. Again, this may be because different genome sequences were used. With respect to grouping of other SGM in the “Matsumoto soft-core gene” tree, see Fig. S13.

For RGM, the “Tortoli ANI” based tree [15, 17] suggest that a higher number of species should belong to another clade than what the “387 core gene” phylogenetic tree suggest (Fig. S13; this study and Ref [16]; for closely related mycobacteria, SGM and RGM, the core gene and ANI based trees were reported to be in agreement [24–26], see also below). In addition to our suggested formation of new clades (see above), a comparison of our “387 core gene” tree and the “Tortoli ANI” tree [15, 17] revealed some notable differences. Our “387 core gene” tree and ANI data suggested that *M. litorale* belongs to the *M. doricum* clade while the “Tortoli ANI” tree [15] positions it close to *M. sphagni* and suggests that *M. doricum* represents a single clade. The “Gupta core gene” tree [16] also positions *M. litorale* in the *M. sphagni* clade. The difference in positioning of *M. litorale* might be related to that we used the DSM 45785 type strain (Table S3a) while Tortoli et al. [15] and Gupta et al. [16] used different isolates. We further noted that the “Tortoli ANI” tree assigns *M. monacense*, *M. gilvum* and *M. pyrenivorans* to the *M. flavescens* clade, while the “387 core gene” tree positioned these DSM type strains in the *M. doricum*, *M. austroafricanum* and *M. pyrenivorans* clades, respectively. The “Tortoli ANI” tree also groups *M. phlei* and *M. hassiacum* together, whereas our two trees assign these mycobacteria to be members of the *M. flavescens* and *M. agri* clades, respectively. Our phylogeny further suggested that *M. thermoresistibile* belongs to the *M. agri* clade in contrast to the “Tortoli ANI” tree [17]. The three mycobacteria, *M. agri*, *M. hassiacum* and *M. thermoresistibile*, constitute the *M. agri* clade in the “387 core gene” (and “56 HC-gene”) phylogeny. The “Tortoli ANI” tree further suggests that *M. wolinskyi* represents a single clade, in contrast to our “387 core gene” phylogeny, which suggested that it is a member of the *M. smegmatis* clade.

The “Gupta core gene” [16] and “Matsumoto soft-core gene” [18] trees grouped *M. rhodesiae* in the *M. gadium* clade while our and the “Tortoli” phylogenies positioned *M. rhodesiae* in the *M. sphagni* clade. Gupta et al. [16] used the NBB3 strain whereas we and Tortoli et al. [15, 17] used the type strain DSM 44223. This might explain the difference in clade/group assignment (Fig. S13); further, Tortoli et al. noted sequence differences between the two *M. rhodesiae* strains and that one was mislabelled [15].

With respect to the two trees reported by Matsumoto et al. [18], we note that the authors emphasize that the soft-core gene reflected the SGM and RGM classifications, with a similar tendency for the hard-core tree. The “Matsumoto soft-core gene” [18] tree shows more differences in the grouping of species than our trees, and the “Gupta core gene”, and “Tortoli ANI” phylogenetic trees (Fig. S13). We further noted that our and the “Tortoli ANI” [17] trees suggest that the *M. chelonae* clade members are the earliest mycobacterial lineage, in contrast to the “Matsumoto core gene” trees. Moreover, in both the “Matsumoto core gene” trees, *M. avium* subsp. *avium* is close to *M. kumamotonense*, which is contrary to our and the “Tortoli ANI” [17] trees. For further differences comparing the “387 core gene” and “Matsumoto soft-core gene” trees, see Fig. S13.

The importance of naming mycobacterial species and strains is illustrated by the recent re-assignments of *M. chubuense* NBB4 as *M. ethylenense* NBB4 [6] and *M. mucogenicum* LZSF01 as a *M. phocaicum* strain, as well as by the differentiation of *M. neoaurum* VKMAc-1815D strain from the *M. neoaurum* type strain [24, 57]. The separation of *M. marinum* into two subspecies, the M- and Aronson-type [25], is also noteworthy. In this context, our core gene trees and ANI data also positioned the type strain *M. farcinogenes* DSM 43637 sequenced in this study close to *M. houstonense* DSM 44676 and not close to *M. senegalense* and *M. conceptionense* (Figs. 2 and S3k). Analysis of the 16S–23S intergenic region according to Hamid et al. [60] suggested 100% sequence identity between our *M. farcinogenes* and *M. farcinogenes* Y10384. We therefore suggest that the NCBI *M. farcinogenes* DSM 43637 likely correspond to a *M. senegalense* strain. The genome sequences for *M. farcinogenes*, *M. senegalense* and *M. conceptionense* type strains also clarify their phylogenetic relationship; see Turenne [10]. We also note that the *M. microti* strain OV254 (NCBI acc. Number AYX01000000) is phylogenetically close to *M. simiae* in both our trees (cf. Figs. 2 and S2). Our ANI value (99.99%; Fig. S3m), together with the fact that *M. microti* is known to be a member of the TB complex, strongly suggests that this isolate/genome sequence represents an *M. simiae* strain.

Our and the reported genome phylogenies [15–18] grouped *M. acapulense* CSURP1424, *M. lehmanni* CECT8763, and *M. neumannii* CECT8766 within, or close to, the *M. flavescens* clade. The ANI values suggest that *M. acapulense* CSURP1424 and *M. lehmanni* CECT8763 should be considered strains of the same species (ANI = 98.12%; Fig. S3c). A comparison of the ANI values for these two isolates with *M. neumannii* CECT8766 suggests that the latter represents a subspecies of these two strains. In this context, we note the ANI values for several of the named mycobacteria (apart from members of the e.g., *M. tuberculosis* and *M. ulcerans* clades) suggest that they should perhaps be considered to be either separate strains or subspecies. For example, the ANI values for several species of the *M. avium* clade are close (*M. yongonense* 05–1390 and *M. chimaera* MCIMRL2/DSM 44623 with ANI > 98%, *M. bouchedurhonense* DSM 45439, *M. timonense* CCUG56329T and *M. avium* 104 with ANI > 99%; Fig. S3e). This also applies to *M. murale* DSM 44340 and *M. tokaiense* DSM 44635 (ANI = 98.22%; Fig. S3j) in the *M. neoaurum* clade. Moreover, including different isolates can also reveal whether these represent the same species, e.g., *M. arupense* GUC1 and DSM 44942, and *M.* sp. UM Kg17 (ANI > 98%; Fig. S3f). With respect to other unnamed mycobacteria, our core gene phylogeny also suggested that (and ANI analysis; Fig. S3) suggested that *M.* sp. M26; *M.* sp. Epa45; *M.* sp. GA-0227b; *M.* sp. IS-3022; *M.* sp. URHB004; and *M.* sp. Root135 should be considered as new mycobacterial species (see also above). As discussed above, in agreement with Tortoli et al. [15], *M. rhodesiae* JS60 should be re-assigned and considered as a new species.

To conclude, together with the recent genome-based mycobacterial phylogeny, our study provides expanded insight into the phylogeny of the *Mycobacterium* genus.

### tRNA and mycobacteria

On the basis of 628 bacteria representing all phyla (see http://lowelab.ucsc.edu/GtRNAdb/, last accessed on May 15th 2020), the average number of tRNA genes is ≈58 (Fig. S14). Among these, bacteria with small genome sizes, such as those belonging to *Chlamydia*, *Spirochetes*, *Tenericutes*, and *Rickettsia* and *Wolbachia* spp., have fewer tRNA genes. For mycobacteria, the average number is 49, which is within the same range as for bacteria in general. Among these we identified 43 core-tRNA genes present in all mycobacteria. We did not detect any correlation between number of tRNA genes and genome size. This is consistent with the situation for several members of *Corynebacteriales* where the average numbers range between 47 and 54 tRNA genes (genome sizes between ≈1.8 and 10.5 Mbp; Figs. 5 and S1a, b; Table S8). Mycobacteria with more than the average number of tRNA genes (or tRNA genes normally not present in mycobacteria or bacteria) have, as we reported recently [24], likely been acquired through HGT. For example, large tRNA clusters present in some mycobacteria were suggested to be associated with HGT events mediated by phages [24] and Refs therein. In this context, an HNH endonuclease homolog and the GOLLD ncRNA gene (Giant, Ornate, Lake- and *Lactobacillales*-Derived [50]) frequently co-localized with large tRNA clusters and hence might be a good marker for the presence of tRNA gene clusters in mycobacteria (Figs. 5 and S5a; for details see Supplementary information, see also Ref [61]). Moreover, since roughly the same average numbers of tRNA genes are present in other *Corynebacteriales* members (Fig. S1b), it is probable that those with higher numbers are also the result of HGT (alternatively, the result of duplications). Consequently, expanding the number of tRNA genes would likely have played a role in the evolution of mycobacteria and possibly also for other *Corynebacteriales* members. We again emphasize that it has been suggested that tRNA genes act as targets to integrate foreign DNA and formation of, e.g., pathogenicity, and metabolic and resistance islands [35, 36] and Refs therein.

### ncRNAs and mycobacteria

As with tRNA, there was no correlation between number of ncRNA genes and genome size. Our data suggested that on average mycobacteria encode 40 ncRNA on average, with the highest numbers, 72 and 73, detected in two SGM, *M. riyadhense* and *M. tusciae*, respectively. We identified 12 core ncRNA, which are present in all mycobacteria. For *M. abscessus*, *M. chelonae* and *M. salmoniphilum* we predicted 52, 34 and 32 ncRNA genes while their close *Corynebacteriales* relatives, *H. subflava* and *S. rotundus* code for 21 and 15 ncRNA, respectively (Table S12). Hence, since all mycobacteria harbor higher numbers of ncRNA genes than *H. subflava* and *S. rotundas* suggests that this might be the result of horizontal gene transfer (HGT) and/or duplications. However, we cannot exclude that the lower numbers in *H. subflava* and *S. rotundus* are the result of a reduction of ncRNA genes. Nevertheless, for the *Mycobacterium* genus we argue that ncRNA genes have been acquired through HGT as well as duplications. In this context, *sar* ncRNA was predicted to be present in the *M. mucogenicum* and *M. canettii* strains, and GOLLD ncRNA in some SGM and RGM, again, probably due to HGT (see above and [24] and Refs therein; see also Ref [61]). More HGT ncRNA candidates were also identified in some other mycobacteria (Fig. 6), e.g., SpF41 (*Streptococcus* sRNA SpF41), cc1840 (*Caulobacter* sRNA cc1840), *yrlA* (Y RNA-like [62]) and ctRNA p42d (*Rhizobium etli* CFN 42 ctRNA_p42d) [41, 42]. Whether these acquired ncRNAs give a selective advantage remains to be studied.

We predicted multiple genes for 6C RNA (in the chromosomes as well as on plasmids) in both SGM and RGM. Although, alignment revealed some differences between regular and extra 6C RNA sequences, they form similar secondary structures with C-rich loops (Fig. S10 and not shown). This suggests that the extra copies have been acquired through HGT but duplications remain a possibility. The 6C RNA is widespread among *Actinobacteria* and data suggested that that it is essential in *M. tuberculosis* [63]. It has been suggested to act as a regulator of multiple genes in *M. smegmatis* and influence cell morphology [64]. For *M. mucogenicum*, we reported that the level of 6C RNA is higher in stationary than in exponential cells [24] and this is also the case for several other mycobacteria (to be published elsewhere). This might indicate that 6C RNA has a role in adaptation to stationary phase. However, since 6C RNA appears to form a stable structure, we cannot exclude the possibility that the higher levels might be due to its high stability. 6C RNA has been implicated to have a role in various cellular processes in other *Actinobacteria*, including sporulation in *S. coelicolor*, SOS response in *C. glutamicum*, and DNA replication and protein secretion in *M. smegmatis* [63–68]. Important questions will be to understand the function of 6C RNA, and how (and if) the extra 6C RNA genes influence the level of 6C RNA, its function and/or growth under various conditions and impact on mycobacterial evolution. An intriguing question is whether the absence of 6C RNA in *M. leprae* affects its growth rate.

We also note that the Ms1 RNA gene, which is positioned close to the 6C RNA gene, is not duplicated in those mycobacteria having more than one copy of the 6C RNA gene. However, the Ms1 RNA gene is present in two copies in *M. nebraskense* and *M. celatum*, although they carry only one 6C RNA gene (see also Ref [24]). Sequence alignment of these two copies suggested structural differences. In addition, a phylogenetic tree based on the Ms1 RNA gene for closely related *Actinobacteria* (not shown) suggested that the second Ms1 RNA gene is also present in the *Rhodococcus jostii* RHA1 and *Smaragdicoccus niligatensis* DSM 44881 genomes as well as on a plasmid associated with the *Rhodococcus* species (Figs. S9 and S15). Thus, the second Ms1 RNA gene may be the result of HGT. The functional consequences, and whether the level for the second Ms1 RNA too is higher in stationary phase as detected for the common Ms1 RNA, which is suggested to influence gene expression when it binds to core RNA polymerase, remains to be investigated [24, 52, 53]. In particular, since both Ms1 RNA and 6C RNA are suggested to act as regulators of gene expression, their role in the evolution of the *Mycobacterium* genus warrants future studies.

We also predicted additional copies for the tmRNA (*ssrA*) and RPR (*rnpB*) genes. For the additional *ssrA* copies, our data suggested that they were homologous to the *ssrA* present in *T. tyrosinosolvens* and on the *Mycobacterium* phage Nappy and plasmids. The function of tmRNA is to remove all components of stalled ribosomal complexes. In these stalled ribosomes, the nascent polypeptide is transferred to tmRNA, which is translated, resulting in a tagged polypeptide that is released and degraded [46]. The extra *ssrA* encodes tmRNAs that when translated result in a peptide, which when translated, has a different sequence than the peptide-tag generated from the “regular” tmRNA. This raises the question whether polypeptides with different tags affect their degradation, and as a consequence, affect growth.

## Conclusions

The mycobacterial genome sizes range from 3.2 to 8.1 Mb with an average of 5.7 Mb. Phages, IS elements, tRNAs, and ncRNAs appear to have contributed to the evolution of the *Mycobacterium* genus where several tRNA and ncRNA genes have been horizontally transferred. Our phylogenetic analysis based of 244 mycobacterial genomes identified several isolates of unnamed species as new mycobacterial species or strains of known mycobacteria. Together with recent publications e.g. [10, 14–18] our findings expand our insight into the evolution of the *Mycobacterium* genus and as such establish a platform for understanding mycobacterial pathogenicity and antibiotic resistance/tolerance among mycobacteria (see *e.g*., Ref [57]). The predicted number of coding sequences correlates with genome size, but the number of tRNA, ncRNA and rRNA genes does not. The number of tRNA and ncRNA genes differ, depending on the mycobacteria. Their positions on the chromosome vary and thereby provide indicators of chromosomal rearrangements during the evolution of mycobacteria. A core set of 12 ncRNAs, 43 tRNAs, and 18 aminoacyl-tRNA synthetases are conserved among the mycobacteria. For ncRNAs, our understanding of their evolution among bacteria is limited [69, 70] and our understanding of their impact on mycobacterial and bacterial evolution is even more so. Access to a large number of mycobacterial genomes is key for addressing these questions.

## Methods

### Cultivation and DNA isolation

Aliquots from cultures of different strains were taken from -80 °C stocks, plated on Middlebrook 7H10 media and incubated at optimal growth temperature (30 °C or 37 °C as recommended) under aerobic conditions. Genomic DNA was isolated as previously described [71].

### Genome sequencing and assembly

A total of 106 genomes were sequenced at the SNP@SEQ Technology Platform (HiSeq2000 – Illumina – platform) and 8 genomes were done at the NGI-Uppsala Genome Center (4 with PacBio Technology and 4 with Ion-Torrent Technology) at Uppsala University.

The PacBio-generated reads were assembled using the SMRT-analysis HGAP3 assembly pipeline [72] and polished using Quiver (Pacific Biosciences, Menlo Park, CA, USA). Assembly of the Illumina generated reads was done using the A5-Assembly pipeline (versions A5-miseq 20,140,604 or 20,160,825; Table S1a) while Ion-Torrent data were assembled using SPAdes (v3.7.0) with minimum contig sizes of 200 bases [73–75].

For genome quality and estimation of genome completeness for the genomes we use the CheckM tool ver 1.1.3 tool. In this tool, the estimation of completeness is based on the presence or absence of lineage-specific marker genes using the checkM lineage_wf [76]. On the basis of the CheckM analysis, species where the contamination level was > 5% were crosschecked for contigs contamination using the tool GUNC ver1.0.5 [77], along with the progenomes v2.1 database [78], and run with the GUNC default parameters and selected “contig_taxonomy_output”. This information along with progenomes type strain was used to decontaminate the contigs (see also Supplementary information).

### Annotation, identification of core genes and ANI analysis

Identification and annotation of coding sequences (CDS) was done using both the Prokka software [version 1.11] [79] and the Rapid Annotation using Subsystem Technology (RAST) server (http://rast.nmpdr.org/) [80]. Functional classification was done using the RAST subsystem classification that uses data both from “The Project to Annotate 1000 genomes” and a collection of protein families referred to as FIGfams. Finally, the listed CDS are those that were predicted by both the Prokka and the RAST server. The annotation program also predicted genes encoding transposases and IS elements.

Core genes were identified as previously reported [6, 24–26, 57]. Briefly, all protein coding sequences (CDS) were extracted from all mycobacterial genomes and used for an “all-vs-all” BLASTp search [81]. Orthologous hard-core genes were identified by using the PanOCT tool (v3.2.3) [82] with the settings, identity ≥45% and query coverage 60% (see also Supplementary information), and the SCARAP v0.3.1 tool [83] with default core-pipeline setting parameters -e, −f and − 1 to 245, 245 and 1, respectively. The 56 hard-core genes were functionally classified into different subsystems (functional roles) using Rapid Annotation using Subsystem Technology (RAST) [6, 80].

The predictions of rRNA and tRNA were done using the RNAmmer [84] and tRNAScan-SE [85, 86] programs, respectively.

Average nucleotide identity (ANI) values were calculated using the pyani tool ver 0.2.11 [87] by comparing the 245 genomes pairwise. The resulting ANI matrix data were clustered using unsupervised hierarchical clustering in R, and plotted using the heatmap.2 package. The ANI values for members of selected clades were also clustered using unsupervised hierarchical clustering and plotted together with the corresponding dendograms [88].

### Plasmid sequences, phage DNA and IS elements

Plasmid sequences were identified from the Illumina sequencing datasets, by using the assembler SPAdes v3.13.1 (plasmidSPAdes, http://cab.spbu.ru/software/plasmid-spades/). The parameter “careful and plasmid” was set along with the defaults setting [73, 89]. The graphical fragment assembly (gfa) format file (from the SPAdes output file) was used as input to the bandage v0.8.1 tool [90] and manually inspected, and circular FASTA sequences were selected. These circular FASTA sequences were validated using cBAR v1.2 [91]. To identify plasmids, we also searched the PLSDB database ver 2021_06_23 [92] and the Mash search tool ver 2.0 with the strategy “mash screen”, default parameters (−v 0.1, −i 0.99), and “winner-takes-all” strategy [93, 94]. All identified plasmids were confirmed by blastN searching against the NCBI database [81, 95]. Blast hits greater than 90% identity and 90% query coverage were considered as known plasmid sequences. Those with lower identity and query coverage were considered to be new plasmid sequences.

The presence of phage sequences was predicted using the PHAGE database at the PHASTER server [33].

The presence of IS elements was predicted using the ISsaga server [32]. Final results were filtered by excluding false positive IS elements and IS elements size less than 600 bps.

### Phylogenetic analysis based on core genes

The 56 and 387 core gene protein sequences from the respective genome sequences were extracted, concatenated and aligned using the tool MAFFT (v7.407) [96]. Phylogenetic trees based on the MAFFT multiple sequence alignment were computed using the FastTree (v2.1.10) [97] with 1000 cycles of bootstrapping and the default settings where FastTree infers approximately-maximum-likelihood phylogenetic trees from the alignment of protein sequences (Jones-Taylor-Thorton +CAT models of amino acid sequences). The figures were generated with the ITOL [98].

### Non-coding (nc) RNAs

Small non-coding RNAs were predicted using the RFAM v13.0 database [41–43] and INFERNAL v1.1.2 [44] with the cmsearch (threshold, −T, cut off value ≥34 and manually filtered eukaryotic type ncRNAs).

Homologs of *M. tuberculosis* H37Rv exponential-phase-related ncRNAs [40] were identified using BLASTn search.

### Statistical calculations

The statistical calculations were performed in R, using a t-test (package stats version 3.6.2) according to Student’s two-sided t-test with no difference as the null hypothesis [88].

## Supplementary Information

**Additional file 1.**

**Additional file 2.**

## Data Availability

All data and materials are available and adheres to BMC Genomics policies on sharing data and materials. Genome sequences have been deposited at NCBI, (nucleotide sequence accession numbers as indicated in Table S1a). Table S1a also includes comments with respect to currently available genomes in relation to the 114 genomes reported in the present study.
