# Supplementary material for: Comparative genome analysis of mycobacteria focusing on tRNA and non-coding RNA
Source: BMC Genomics. 2022 Oct 15;23:704. doi: 10.1186/s12864-022-08927-5 (PMC9569102; doi:10.1186/s12864-022-08927-5)
Supplement: Supplementary file 1 — Additional file 1. [file 12864_2022_8927_MOESM1_ESM.pdf]

Supplementary information

Comparative genome analysis of Mycobacteria focusing on tRNA and non-coding RNA

Phani Rama Krishna Behra[1], B. M. Fredrik Pettersson[1], Malavika Ramesh, Sarbashis Das, Santanu Dasgupta and Leif A. Kirsebom*

Department of Cell and Molecular Biology
Box 596, Biomedical Centre
SE-751 24 Uppsala, Sweden

*Corresponding author
Tel no +46 18 471 4068
Fax no +46 18 53 03 96
Email Leif.Kirsebom@icm.uu.se

[1]Equal contributions to this study

Running title: Comparative genomic analysis of the *Mycobacterium* genus

Key words: Mycobacterial genomes, core gene phylogeny, tRNA and non-coding RNA

Supplementary information

Supplementary Methods and Results

Supplementary Tables: Table of contents and Tables S1-S12.

Supplementary Figures: Figures S1-S15.

Supplementary Methods and Results

Genome quality

The estimated genome quality of the majority of the 245 (including *H. subflava* DQS3-9A1) genomes showed that the majority were near complete, >95%, except for four: *M. interjectum* DSM 44064 (94.08%), *M. insubricum* DSM 45132 (92.08%), *M. lepromatosis* strain FJ924 (81.06%), and *M. leprae* TN (80.17%); the two latter correspond to the NCBI complete genomes. According to CheckM contamination levels and classification[76] 33 genomes were not contaminated (*i.e*., 0%), 207 had low levels (<5%), and five genomes showed >5% contamination. Among these five genomes, *M. saskatchewanense* DSM 44616 (41.66%) was very highly contaminated, *M. gadium* DSM 44077 (10.68%) highly contaminated, and three genomes - *M. vaccae* DSM 43292 (9.24%), *M. nonchromogenicum* DSM 44164 (8.90%) and *M. setense* DSM 45070 (5.80%) - were moderately contaminated. These five genomes were decontaminated based on "contig_taxonomy" classification using the tool GUNC ver 1.0.5 (and progenomes v2.1 database)[77,78]. The decontaminated contigs were re-checked for genome quality using CheckM[76] (see Table S1b) and the updated contaminated levels showed <2% for four species and 5.76% for *M. nonchromogenicum* DSM 44164. The strain *M. nonchromogenicum* genome contained many shorter contigs. Due to this CheckM marker genes were shown multiple times and thus resulted in higher contaminated level (5.76%). Finally, we compared the ANI values for these five genomes against reference genomes in the progenomes v2.1 database (see Fig S3n).

Identification of hard-core genes – a comparison

As described in the Methods, we used two methods to identify homologous genes/orthogroups in the 244 mycobacterial genomes and in the *H. subflava* genome. Using the PanOCT tool, we identified 56 hard-core protein genes, referred to as "56 HC-genes" (>45% identity and >60% query coverage). With the SCARAP v0.3.1 pipeline we identified 387 orthogroups using the core-pipeline, hereafter referred to as the "387 core genes". Others used different approaches to identify core genes as briefly outlined below.

Fedrizzi *et al*. found 243 genes, referred to as core genes, present in 99 mycobacterial genomes using a different approach. They clustered all genes present in the genomes (80% nucleotide identity), then filtered the clusters based on the completeness of the different mycobacteria. The sequences of the selected clusters were subsequently used as query sequences in a BLAST search discarding matches less than 50% identity and shorter than 50% in length to the closet reference sequence. They also used the Roary method and identified 179 core genes (for details see Ref 14).

Gupta *et al*. identified 1941 core proteins in 150 mycobacterial genomes based on >50% sequence identity and sequence length and found in at least 80% of the input genomes (for details see Ref 16).

Matsumoto *et al*. identified 288 core genes in 175 mycobacteria by clustering orthologs based on ≥80% amino acid sequence identity and considering a length ratio ≤0.8, and 1306 core genes present in 80% of the analyzed mycobacterial genomes (for details see Ref 18).

Phylogenies based on "56 HC-genes" and "387 core genes" - a comparison

In the main text we focused on the phylogeny based on "387 core genes". This phylogenetic tree is in overall agreement with the tree based on "56 HC-genes", which is also supported by our ANI analysis. However, there were some differences between the two trees (cf. Fig 2 and

Fig S2a, and see Fig S2b for a comparison). See also the discussion in the main text where we compare the "387 core gene" tree with ANI based trees ("Tortoli" trees[14,15,17]), the "Gupta" tree[16] based on 1941 soft-core genes and "Matsumoto" tree[18] based on 288 hard core genes (and 1306 "soft-core" genes) in 175 mycobacteria.

In contrast to the "56 HC-gene" tree, the "387 core gene" tree suggested that *M. duvalii* and *M. poriferae* are not members of the *M. chlorophenolicum* clade. Positioning of *M. duvalii* outside the *M. chlorophenolicum* clade is in keeping with the ANI based "Tortoli" tree[17] and the "Matsumoto" soft-core tree[18] (see the discussion in the main text). Hence, *M. duvalii* and *M. poriferae* constitute the *M. duvalii* clade. Moreover, while *M. murale* and *M. tokaiense* are grouped in the *M. neoaurum* clade in the "56 HC-gene" tree, these two mycobacteria are positioned in the *M. chitae* clade in the "387 core gene" tree, similar to the positioning in the "Matsumoto" tree[18]. The "387 core gene" tree suggested 6 single clades, all located among SGM, while the "56 HC-gene" tree suggested 8 single clades. In the "56 HC-gene" tree, *M. smegmatis* is represented as a single clade but is grouped together in the "387 core gene" tree with *M. goodii* and *M. wolinskyi* constituting the *M. smegmatis* clade. This agrees with *M. smegmatis* being close to *M. goodii* and *M. wolinskyi* in the "Gupta"[16], "Tortoli"[15] and "Matusumoto 1306 core gene"[18] trees. The "387 core gene" tree positioned *M. riyadhense* among the *M. gordonae* clade members (in keeping with its closeness to other members of this clade[15-18]) while it constitutes a single clade member in the "56 HC-gene" tree (this is questionable given the bootstrap values; Fig S2a). In the "387 core gene" tree, *M. noviomagense* is a member of the *M. xenopi* clade, matching previous data[15-18], while it is considered as a single member clade in the "56 HC-gene" tree.

Our two trees also differed with respect to positioning of whole clades where the *M. doricum* clade represents an earlier "lineage" relative to the *M. flavescens*, *M. elephantis*, *M. gadium*, *M. agri*, *M. chlorophenolicum*, *M. austroafricanum* and *M. pyrenivorans* clades in the "387 core

gene" tree. The locations of *M. chitae*, *M. sphagni* and *M. simiae* also differ in the two trees and the position changes for some species as well (cf. Fig 2 and Fig S2a). Finally, considering the earliest lineage, the *M. chelonae* clade, we note that *M. franklinii* is closer to *M. abscessus* in the "387 core gene" tree, in agreement with our earlier findings[26] and with the previously reported genome based phylogenies[15-18].

Analysis of similarities of IS-element types among mycobacteria

To compare the similarity of IS-element types in each species and strains we calculated an "IS-similarity score". First, we determined if a specific IS-element type was present (disregarding the number of copies of each type), or absent. Then a pairwise "IS-similarity score" was calculated by adding the number of IS-element types either present or absent in both species and dividing the obtained sum with the total number of IS-element types present in all the mycobacterial genomes. The pairwise "IS-similarity scores" were multiplied by 100 to generate the "IS-similarity score" as a percentage identity. The IS-similarity scores were plotted in a heat map (Fig S4) sorted according to the "387 core gene" tree (Figs 2).

Prediction of aminoacyl-tRNA-synthetase paralogs

As we recently reported in a study analyzing members of the *M. mucogenicum* and *M. neoarum* clades mycobacteria in general encode for AARS paralogs such as the GluRS and LysRS paralogs[24]. Except for the deeply rooted *M. chelonae* clade, the RGM have two predicted GluRS genes (1.87 on average, including the *M. chelonae* clade; Table S9a). This is significantly more than the SGM (1.07; $p = 1.98 \times 10^{-45}$). For LysRS, two to three genes were predicted among RGM (2.38 on average), whereas SGM encoded for significantly higher numbers (average 2.9; $p = 8.1 \times 10^{-8}$). However, among the slow growers, the *M. terrae* and *M. triviale* clade members only carry one or two predicted LysRS genes.

For the non-pathogenic mycobacteria, the average numbers of GluRS genes (1.84) were predicted to be higher than for both pathogens (1.08; $p = 2.35x10^{-11}$) and opportunistic pathogens (1.37; $p = 9.98x10^{-10}$). The difference between the pathogens and opportunistic pathogens was significant ($p = 0.00445$) for GluRS, but not for LysRS. However, both pathogens (2.80) and opportunistic pathogens (2.85) carry higher numbers of LysRS genes than the non-pathogens (2.30; $p = 0.000654$ and $p = 6.75\ x10^{-7}$, respectively). Thus, there appears to be no correlation between a high number of tRNA genes and total number of AARS. For a discussion of possible functions for these AARS paralogs see Ref[24].

GOLLD ncRNA and HNH endonuclease as a predictor of presence of tRNA genes

HNH endonucleases are DNA endonucleases. These DNA endonucleases are associated with mobile genetic elements like transposons and phages, but also with bacterial restriction enzymes and transcription factors (Stoddard *et al.*, 2005; 2011; see also Kala *et al.*, 2014). Genomes having the HNH endonuclease gene closely linked to tRNA clusters have significantly higher numbers of tRNA genes (67.1) on average compared to mycobacteria lacking this gene (46.8; $p = 1.91\ x10^{-54}$). Mycobacteria encoding for both the "tRNA linked" HNH endonuclease and GOLLD ncRNA have significantly higher numbers of tRNA genes (82.4) on average compared to those encoding only for the HNH endonuclease (62.4; $p = 0.000154$) and those lacking both genes (46.8; $p = 4.36\ x10^{-108}$). We conclude that the presence of this specific "tRNA linked" HNH endonuclease homolog alone is a strong predictor of mycobacteria having a high number of tRNA genes, and that the presence of both genes is a predictor of even higher numbers of tRNA genes (we identified no mycobacteria encoding GOLLD and lacking the HNH endonuclease, see also Ref[61]).

Mycobacteria a Corynebacteriales *family member*

The *Mycobacterium* genus belongs to the family *Corynebacteriales*. The size of the genomes for mycobacteria range between 3.2 to 8.1 Mbp with an average size of 5.7 Mbp. For other members of the family[3,56], *e.g. Corynebacterium* spp., *Nocardia* spp. and *Rhodococcus* spp., the average genome size varies between 2.6 to 7.8 Mbp (Fig S1a). Among these, the mycobacterial average genome size is smaller than for *Nocardia* spp. (7.8 Mbp, n = 81) and *Rhodococcus* spp. (6.1 Mbp, n = 32), but larger than *Corynebacterium* spp. (2.6 Mbp, n = 114) and *Segniliparus* spp. (3.4 Mbp, n = 2). Thus, the range in genome size varies within the family *Corynebacteriales*. Because *Segniliparus* spp. and mycobacteria share a common ancestor[56] this might also indicate size reduction for *Segniliparus* spp. genomes after these two phylogenetically close lineages diverged. According to Coimbra *et al*., however, *H. subflava* (4.7 Mbp; originally isolated from crude oil-polluted soil[22]), is closer to mycobacteria than *Segniliparus* species and it belongs to the *Mycobacteriaceae* family (NCBI taxonomy). To gain further insight into the evolution of species in these lineages we aligned the complete genomes of *M. abscessus*, *M. chelonae* and *M. salmoniphilum*, which all belong to the earliest mycobacterial lineage. Their genome sizes are ≈5 Mbp (Fig 2)[14-17,26], similar to the genome size of *H. subflava* but different compared to *S. rotundus* (Refs 22 and Sikorski et al., 2010). This alignment revealed significant differences with respect to their genomic organizations. The mycobacteria and *H. subflava* showed the highest similarity (Fig S12a) in keeping with the NCBI taxonomy (Coimbra *et al*., 2020). Moreover, 1705 core CDS were predicted to be present in both *M. chelonae* and *H. subflava*; for *H. subflava* this represents 39% of identified CDS. Thus, despite phylogenetic proximity and presence of gene homologs, we detected clear differences in genome organization and in the occurrence of high numbers of unique genes in both *M. chelonae* and *H. subflava* (3189 and 2714 CDS, respectively; Fig S12b). When we compared *M. abscessus* and *S. rotundus* 1444 core CDS were common whereas there were 1716 in *H. subflava* (Fig S12c, d; see also Fig S12e-g).

Together this suggest that chromosomal rearrangement occurred prior to the formation of the earliest mycobacterial lineage (for alignment of *M. chelonae* clade members see Fig S1 in Ref 26). Interestingly, *S. rotundus* was originally isolated from human sputum indicating that it is present in the same ecological niche as mycobacteria that cause respiratory infections, *e.g. M. abscessus* (Butler et al., 2005; Koh et al., 2011; Keika, 2018). This raises questions about evolutionary forces acting on these two species.

Supplementary References

Stoddard, B. L. Homing endonuclease structure and function. *Q. Rev. Biophys.* **38**, 49–95 (2005).

Kala, S., Cumby, N., Sadowski, P.D., Hyder, B.Z., Kanelis, V., Davidson, A.R. & Maxwell, K.L. HNH proteins are a widespread component of phage DNA packaging machines. *Proc Natl Acad Sci USA* **111**, 6022–6027 (2014).

Stoddard, B. L. Homing endonucleases: From microbial genetic invaders to reagents for targeted DNA modification. *Structure* **19**, 7–15 (2011).

Coimbra, N.D.R., Goes-Neto, A., Azevedo, V. & Ouangraoua, A. Reconstructing the phylogeny of Corynebacteriales while accounting for horizontal gene transfer. *Genome Biol Evol* **12**, 381-395 (2020).

Sikorski, J. et al. Complete genome sequence of Segniliparus rotundus type strain (CDC 1076^T). *Stand Genomic Sci* **2**, 203-211 (2010).

Butler, W.R., Floyd, M.M., Brown, J.M., Toney, S.R., Daneshvar, M.I., Cooksey, R.C., Carr, J., Steigerwalt, A.G. & Charles, N. Novel mycolic acid-containing bacteria in the family Segniliparaceae fam. nov., including the genus Segniliparus gen. nov., with descriptions of Segniliparus rotundus sp. nov. and Segniliparus rugosus sp. nov. *Int J Syst Evol Microbiol* **55**, 1615-1624 (2005).

Koh, W-J., Choi, G-E., Lee, S-H., Park, Y.K., Lee, N.Y. & Shin, S.J. First case of Segniliparus rotundus pneumonia in a patient with bronchiectasis. *J Clin Microbiol* **49**, 3403-3405 (2011).

Keikha, M. Importance of the identification of Segniliparus species from pulmonary infection. New Microbes New Infect 25, 1-2 (2018).

Supplementary figure legends

Figure S1 Genome size and tRNA content distribution in Actinobacteria

(A) The genome sizes of different genuses within the Actinobacteria family were obtained from the NCBI database (https://www.ncbi.gov/genome/browse#!/prokaryotes/actinobacteria), last access on May 15th 2020, filtered for representative genomes and plotted as box plots where the thick horizontal black line represents the median genome size. The upper and lower bounds of the boxes represent the second and third quartiles, and the whiskers represent the first and fourth quartiles. Outliers are represented as circles. The n-values represent the number of species in each genus.

(B) The numbers of tRNA genes of the same genuses as in (A) are shown as boxplots where the different values are represented with the same meaning as in (A).

Fig S1

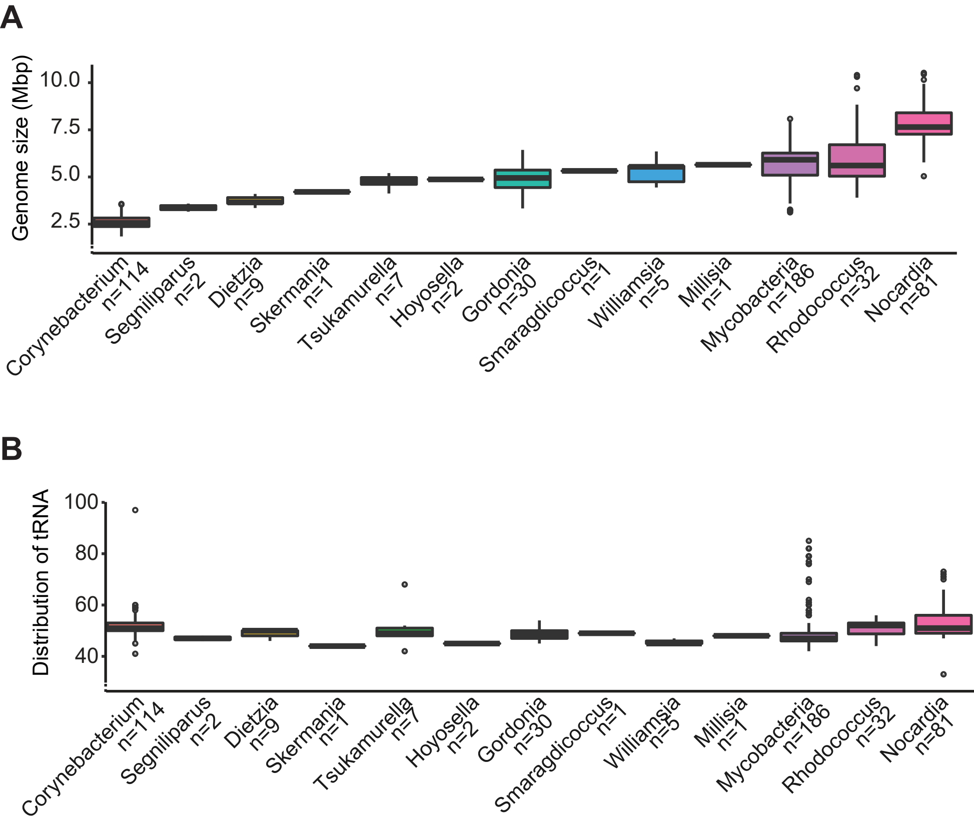
A
Genome size (Mbp)
10.0
7.5
5.0
2.5
Corynebacterium n=114
Segniliparus n=2
Dietzia n=9
Skermania n=1
Tsukamurella n=7
Hoyosella n=2
Gordonia n=30
Smaragdicoccus n=1
Williamsia n=5
Millisia n=1
Mycobacteria n=186
Rhodococcus n=32
Nocardia n=81
B
Distribution of tRNA
100
80
60
40
Corynebacterium n=114
Segniliparus n=2
Dietzia n=9
Skermania n=1
Tsukamurella n=7
Hoyosella n=2
Gordonia n=30
Smaragdicoccus n=1
Williamsia n=5
Millisia n=1
Mycobacteria n=186
Rhodococcus n=32
Nocardia n=81

Figure S2 "56 HC-gene" gene phylogeny of Mycobacteria and comparison of "387 core gene" and "56 HC-gene" phylogenies

(A) phylogeny based on 56 hard-core protein genes present in all mycobacteria ("hard-core proteins") was calculated as described in Methods. The tree is divided into slow (SGM, orange) and rapid growing mycobacteria (RGM, green); black indicates no information was available to determine growth rate ("unknown"). Bootstrap support values from 1000 cycles are indicated as colored dots at the respective nodes (if 90% or above), or by their actual values (below 90%). Mycobacterial clades are indicated by boxes; vertical text to the right of the boxes refers to the clade names while species positioned outside the boxes represent single-species clades. Pairwise ANI values were calculated for all the genomes and the branches of the tree are colored according to these values (see legend to the left and Table S4). We emphasize that to color the branches on both sides of a connecting node, all species on one side of the node must have ANI values within the range compared with all other species on the other side of the node and *vice versa*. Individual genomes may have ANI values that are higher than the range of values indicated by the coloring compared with one or more genomes on the other side of the node.

Underlined species were sequenced in this study, while species marked with black dots are previously reported[6,24-27]. *Marks the positioning of *M. farcinogenes* DSM 43637 strain sequenced in this study; the other *M. farcinogenes* DSM 43637 strain corresponds to the available genome sequence at the NCBI database, see main text for details.

##Marks the isolate *M. microti* OV254, which based on our combined data should be considered as a *M. simiae* strain (see main text and also Fig 2).

(B) Comparison of the "56 HC-gene" and "387 core gene" trees

The "387 core gene" phylogenetic tree (Fig 2) collapsed to the clade level is shown to the left. Arrows indicate differences compared to the "387 core gene" tree (Fig 2). "From" indicates to

which clade the indicated species belongs in the "387 core gene" tree. "To" indicates the assigned clades for the marked species using the other phylogenetic trees as indicated. The thickness of the dashed arrows indicates number of mycobacteria that changed their position in the two trees; see the legend to the left of the "387 core gene" tree. Mycobacteria marked with colored dots (• and •) in front of the name represent single-species clades in the respective tree.

Fig S2

A

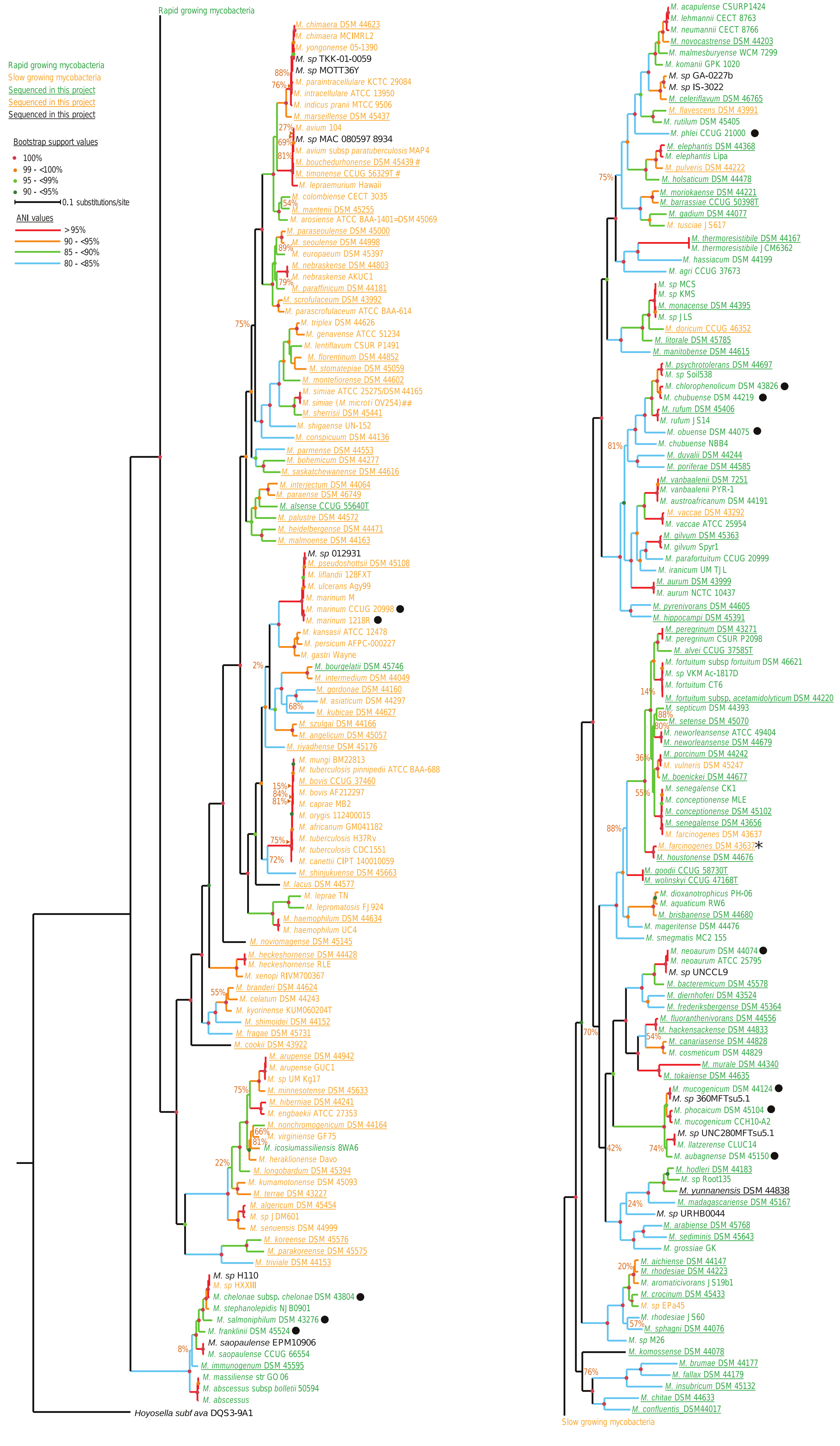

Rapid growing mycobacteria
Slow growing mycobacteria
Sequenced in this project
Sequenced in this project
Sequenced in this project
Bootstrap support values
100%
99 - <100%
95 - <99%
90 - <95%
0.1 substitutions/site
ANI values
>95%
90 - <95%
85 - <90%
80 - <85%
Rapid growing mycobacteria
M. chimaera DSM 44623
M. chimaera MCIMRL2
M. yongonense 05-1390
M. sp TKK-01-0059
M. sp MOTT36Y
M. paraintracellulare KCTC 29084
M. intracellulare ATCC 13950
M. indicus pranii MTCC 9506
M. marseillense DSM 45437
M. avium 104
M. sp MAC 080597 8934
M. avium subsp paratuberculosis MAP4
M. bouchedurhonense DSM 45439 #
M. timonense CCUG 56329T #
M. lepraemurium Hawaii
M. colombiense CECT 3035
M. mantenii DSM 45255
M. arosiense ATCC BAA-1401=DSM 45069
M. paraseoulense DSM 45000
M. seoulense DSM 44998
M. europaeum DSM 45397
M. nebraskense DSM 44803
M. nebraskense AKUC1
M. paraffinicum DSM 44181
M. scrofulaceum DSM 43992
M. parascrofulaceum ATCC BAA-614
M. triplex DSM 44626
M. genavense ATCC 51234
M. lentiflavum CSUR P1491
M. florentinum DSM 44852
M. stomatepiae DSM 45059
M. montefiorense DSM 44602
M. simiae ATCC 25275/DSM 44165
M. simiae (M. microti OV254)##
M. sherrisii DSM 45441
M. shigaense UN-152
M. conspicuum DSM 44136
M. parmense DSM 44553
M. bohemicum DSM 44277
M. saskatchewanense DSM 44616
M. interjectum DSM 44064
M. paraense DSM 46749
M. alsense CCUG 55640T
M. palustre DSM 44572
M. heidelbergense DSM 44471
M. malmoense DSM 44163
M. sp 012931
M. pseudoshottsii DSM 45108
M. liflandii 128FXT
M. ulcerans Agy99
M. marinum M
M. marinum CCUG 20998
M. marinum 1218R
M. kansasii ATCC 12478
M. persicum AFPC-000227
M. gastri Wayne
M. bourgelatii DSM 45746
M. intermedium DSM 44049
M. gordonae DSM 44160
M. asiaticum DSM 44297
M. kubicae DSM 44627
M. szulgai DSM 44166
M. angelicum DSM 45057
M. riyadhense DSM 45176
M. mungi BM22813
M. tuberculosis pinnipedii ATCC BAA-688
M. bovis CCUG 37460
M. bovis AF212297
M. caprae MB2
M. orygis 112400015
M. africanum GM041182
M. tuberculosis H37Rv
M. tuberculosis CDC1551
M. canettii CIPT 140010059
M. shinjukuense DSM 45663
M. lacus DSM 44577
M. leprae TN
M. lepromatosis FJ924
M. haemophilum DSM 44634
M. haemophilum UC4
M. noviomagense DSM 45145
M. heckeshornense DSM 44428
M. heckeshornense RLE
M. xenopi RIVM700367
M. branderi DSM 44624
M. celatum DSM 44243
M. kyorinense KUM060204T
M. shimoidei DSM 44152
M. fragae DSM 45731
M. cookii DSM 43922
M. arupense DSM 44942
M. arupense GUC1
M. sp UM Kg17
M. minnesotense DSM 45633
M. hiberniae DSM 44241
M. engbaekii ATCC 27353
M. nonchromogenicum DSM 44164
M. virginiense GF75
M. icosiumassiliensis 8WA6
M. heraklionense Davo
M. longobardum DSM 45394
M. kumamotonense DSM 45093
M. terrae DSM 43227
M. algericum DSM 45454
M. sp JDM601
M. senuense DSM 44999
M. koreense DSM 45576
M. parakoreense DSM 45575
M. triviale DSM 44153
M. sp H110
M. sp HXXIII
M. chelonae subsp. chelonae DSM 43804
M. stephanolepidis NJB0901
M. salmoniphilum DSM 43276
M. franklinii DSM 45524
M. saopaulense EPM10906
M. saopaulense CCUG 66554
M. immunogenum DSM 45595
M. massiliense str GO 06
M. abscessus subsp bolletii 50594
M. abscessus
Hoyosella subflava DQS3-9A1
88%
76%
27%
69%
81%
54%
89%
79%
75%
2%
68%
15%
84%
81%
75%
72%
55%
75%
66%
81%
22%
8%
M. acapulense CSURP1424
M. lehmannii CECT 8763
M. neumannii CECT 8766
M. novocastrense DSM 44203
M. malmesburyense WCM 7299
M. komanii GPK 1020
M. sp GA-0227b
M. sp IS-3022
M. celeriflavum DSM 46765
M. flavescens DSM 43991
M. rutilum DSM 45405
M. phlei CCUG 21000
M. elephantis DSM 44368
M. elephantis Lipa
M. pulveris DSM 44222
M. holsaticum DSM 44478
M. moriokaense DSM 44221
M. barrassiae CCUG 50398T
M. gadium DSM 44077
M. tusciae JS617
M. thermoresistibile DSM 44167
M. thermoresistibile JCM6362
M. hassiacum DSM 44199
M. agri CCUG 37673
M. sp MCS
M. sp KMS
M. monacense DSM 44395
M. sp JLS
M. doricum CCUG 46352
M. litorale DSM 45785
M. manitobense DSM 44615
M. psychrotolerans DSM 44697
M. sp Soil538
M. chlorophenolicum DSM 43826
M. chubuense DSM 44219
M. rufum DSM 45406
M. rufum JS14
M. obuense DSM 44075
M. chubuense NBB4
M. duvalii DSM 44244
M. poriferae DSM 44585
M. vanbaalenii DSM 7251
M. vanbaalenii PYR-1
M. austroafricanum DSM 44191
M. vaccae DSM 43292
M. vaccae ATCC 25954
M. gilvum DSM 45363
M. gilvum Spyr1
M. parafortuitum CCUG 20999
M. iranicum UM TJL
M. aurum DSM 43999
M. aurum NCTC 10437
M. pyrenivorans DSM 44605
M. hippocampi DSM 45391
M. peregrinum DSM 43271
M. peregrinum CSUR P2098
M. alvei CCUG 37585T
M. fortuitum subsp fortuitum DSM 46621
M. sp VKM Ac-1817D
M. fortuitum CT6
M. fortuitum subsp. acetamidolyticum DSM 44220
M. septicum DSM 44393
M. setense DSM 45070
M. neworleansense ATCC 49404
M. neworleansense DSM 44679
M. porcinum DSM 44242
M. vulneris DSM 45247
M. boenickei DSM 44677
M. senegalense CK1
M. conceptionense MLE
M. conceptionense DSM 45102
M. senegalense DSM 43656
M. farcinogenes DSM 43637
M. farcinogenes DSM 43637*
M. houstonense DSM 44676
M. goodii CCUG 58730T
M. wolinskyi CCUG 47168T
M. dioxanotrophicus PH-06
M. aquaticum RW6
M. brisbanense DSM 44680
M. mageritense DSM 44476
M. smegmatis MC2 155
M. neoaurum DSM 44074
M. neoaurum ATCC 25795
M. sp UNCCL9
M. bacteremicum DSM 45578
M. diernhoferi DSM 43524
M. frederiksbergense DSM 45364
M. fluoranthenivorans DSM 44556
M. hackensackense DSM 44833
M. canariasense DSM 44828
M. cosmeticum DSM 44829
M. murale DSM 44340
M. tokaiense DSM 44635
M. mucogenicum DSM 44124
M. sp 360MFTsu5.1
M. phocaicum DSM 45104
M. mucogenicum CCH10-A2
M. sp UNC280MFTsu5.1
M. llatzerense CLUC14
M. aubagnense DSM 45150
M. hodleri DSM 44183
M. sp Root135
M. yunnanensis DSM 44838
M. madagascariense DSM 45167
M. sp URHB0044
M. arabiense DSM 45768
M. sediminis DSM 45643
M. grossiae GK
M. aichiense DSM 44147
M. rhodesiae DSM 44223
M. aromaticivorans JS19b1
M. crocinum DSM 45433
M. sp EPa45
M. rhodesiae JS60
M. sphagni DSM 44076
M. sp M26
M. komossense DSM 44078
M. brumae DSM 44177
M. fallax DSM 44179
M. insubricum DSM 45132
M. chitae DSM 44633
M. confluentis DSM44017
Slow growing mycobacteria
75%
81%
14%
88%
80%
36%
55%
88%
70%
54%
42%
74%
24%
20%
57%
76%

Fig S2
B

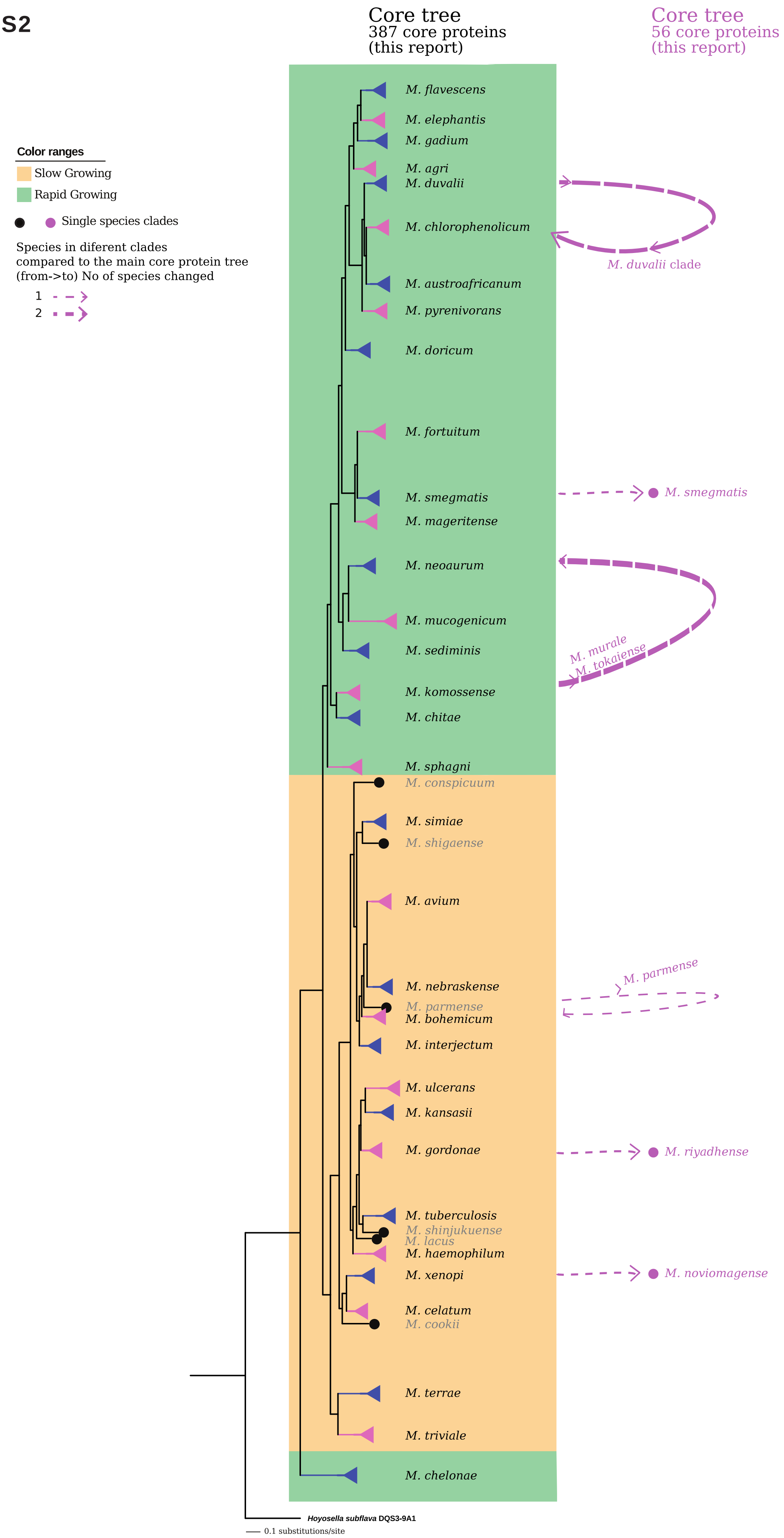

Core tree
387 core proteins
(this report)
Core tree
56 core proteins
(this report)
Color ranges
Slow Growing
Rapid Growing
Single species clades
Species in diferent clades
compared to the main core protein tree
(from->to) No of species changed
1
2
M. flavescens
M. elephantis
M. gadium
M. agri
M. duvalii
M. chlorophenolicum
M. austroafricanum
M. pyrenivorans
M. doricum
M. fortuitum
M. smegmatis
M. mageritense
M. neoaurum
M. mucogenicum
M. sediminis
M. komossense
M. chitae
M. sphagni
M. conspicuum
M. simiae
M. shigaense
M. avium
M. nebraskense
M. parmense
M. bohemicum
M. interjectum
M. ulcerans
M. kansasii
M. gordonae
M. tuberculosis
M. shinjukuense
M. lacus
M. haemophilum
M. xenopi
M. celatum
M. cookii
M. terrae
M. triviale
M. chelonae
Hoyosella subflava DQS3-9A1
0.1 substitutions/site
M. duvalii clade
M. smegmatis
M. murale
M. tokaiense
M. parmense
M. riyadhense
M. noviomagense

Figure S3 Heat map showing ANI values for "all-versus-all" 244 mycobacteria and for selected clades

(A) ANI values were plotted using the heatmap.2 package in R. On the basis of the "387 core gene" phylogenetic tree (Fig 2), the different clades are shown on the right-hand side; see main text for details.

(B-N) ANI values for mycobacteria assigned to selected clades as identified in Fig 2 were clustered based on unsupervised hierarchial clustering, then plotted together with the dendograms (see Methods). ANI values for members of;

(B) the *M. sphagni* clade,

(C) the *M. flavescens* clade,

(D) the *M. sediminis* clade,

(E) the *M. avium* clade,

(F) the *M. terrae* clade,

(G) the *M. ulcerans* clade,

(H) the *M. chelonae* clade,

(I) the *M. doricum* clade,

(J) the *M. neoaurum* clade,

(K) the *M. fortuitum* clade,

(L) the *M. gordonae* clade, and

(M) the *M. simiae* clade.

(N) ANI values for *M. saskatchewanense* DSM 44616, *M. gadium* DSM 44077, *M. vaccae* DSM 43292, *M. nonchromogenicum* DSM 44164 and *M. setense* DSM 45070 and reference genomes in the progenomes v2.1 database (see Supplementary Methods).

* Marks the genomes sequenced in this study.

Fig S3
A

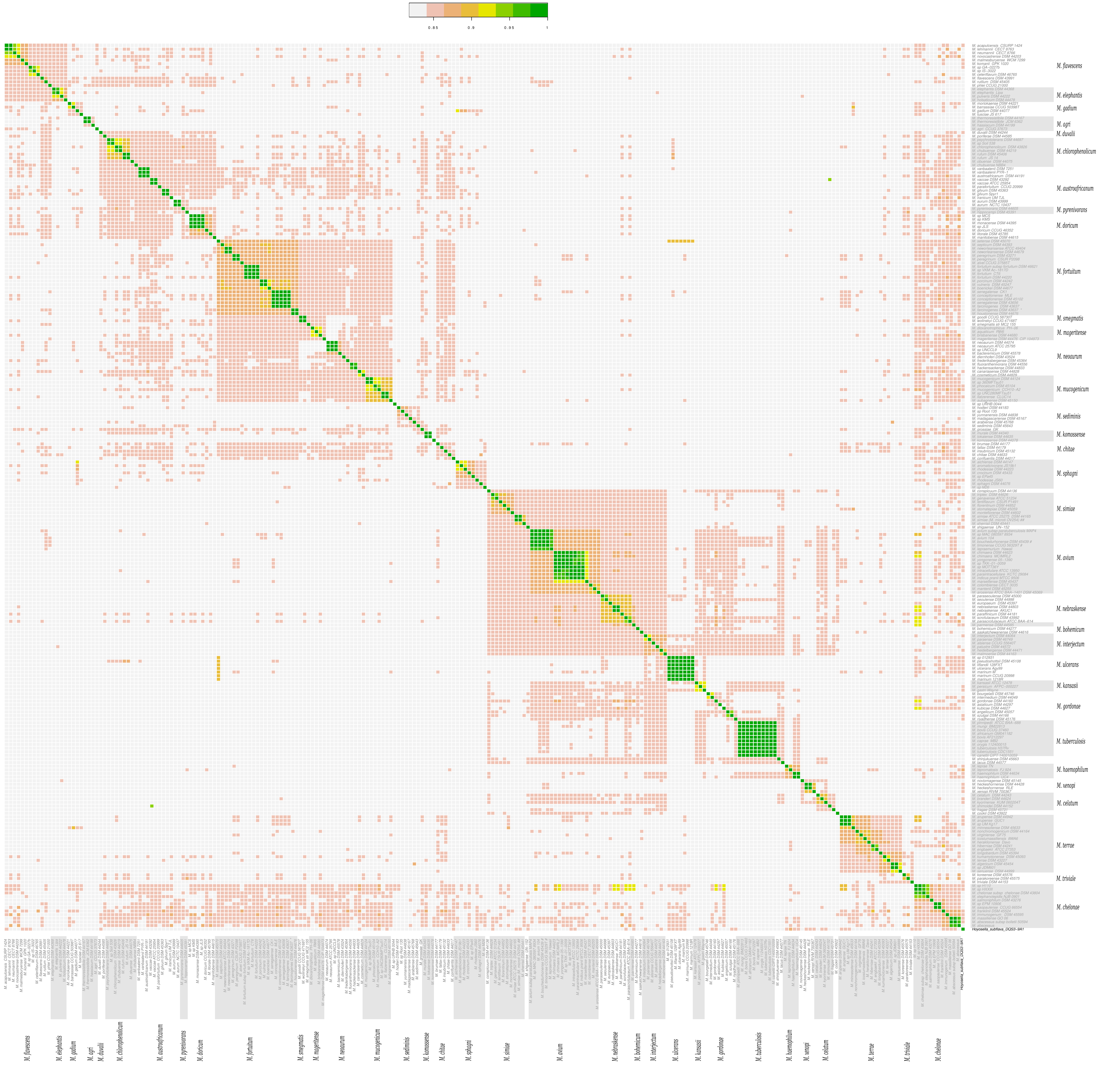
0.85
0.9
0.95
1
M. flavescens
M. elephantis
M. gadium
M. agri
M. duvalii
M. chlorophenolicum
M. austroafricanum
M. pyrenivorans
M. doricum
M. fortuitum
M. smegmatis
M. mageritense
M. neoaurum
M. mucogenicum
M. sediminis
M. komossense
M. chitae
M. sphagni
M. simiae
M. avium
M. nebraskense
M. bohemicum
M. interjectum
M. ulcerans
M. kansasii
M. gordonae
M. tuberculosis
M. haemophilum
M. xenopi
M. celatum
M. terrae
M. triviale
M. chelonae

Fig S3

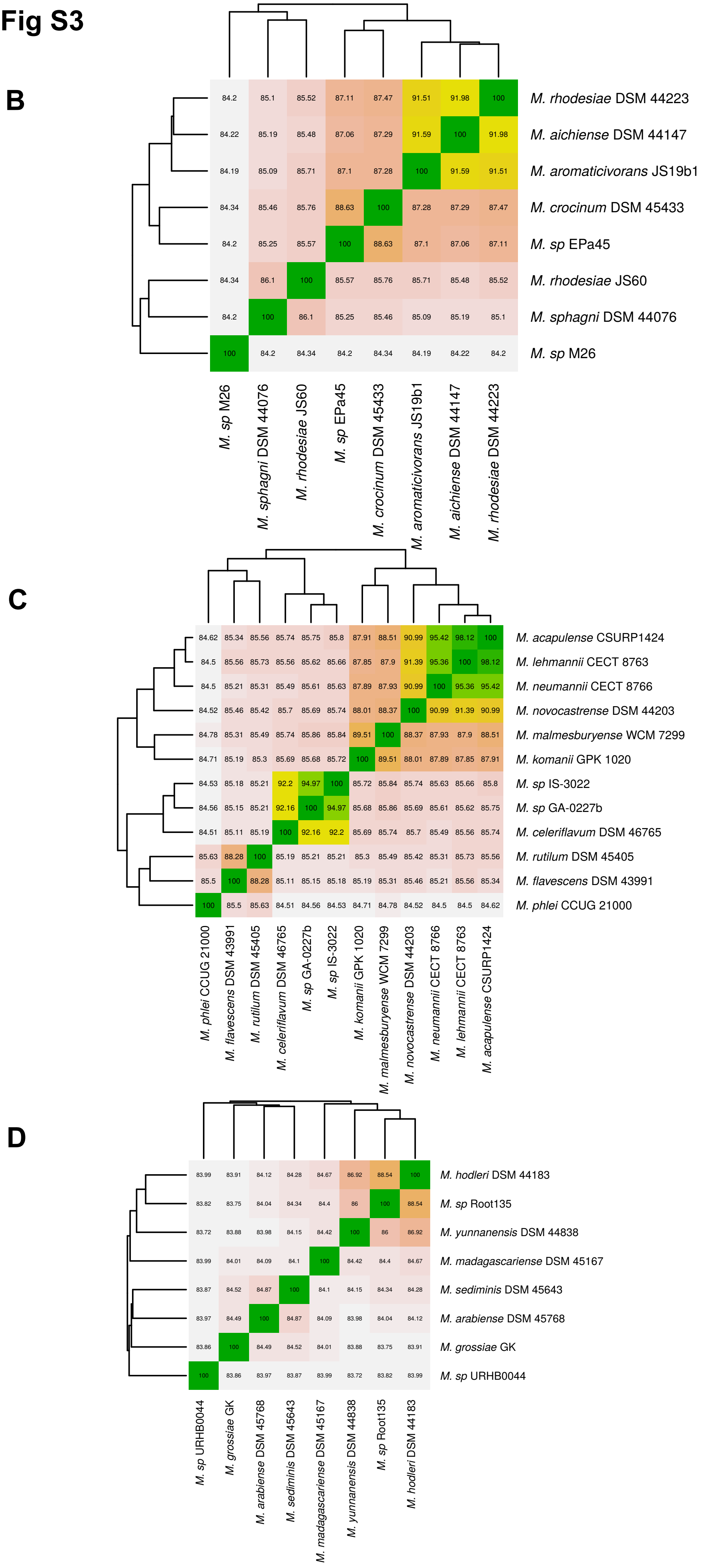
B
84.2 85.1 85.52 87.11 87.47 91.51 91.98 100 M. rhodesiae DSM 44223
84.22 85.19 85.48 87.06 87.29 91.59 100 91.98 M. aichiense DSM 44147
84.19 85.09 85.71 87.1 87.28 100 91.59 91.51 M. aromaticivorans JS19b1
84.34 85.46 85.76 88.63 100 87.28 87.29 87.47 M. crocinum DSM 45433
84.2 85.25 85.57 100 88.63 87.1 87.06 87.11 M. sp EPa45
84.34 86.1 100 85.57 85.76 85.71 85.48 85.52 M. rhodesiae JS60
84.2 100 86.1 85.25 85.46 85.09 85.19 85.1 M. sphagni DSM 44076
100 84.2 84.34 84.2 84.34 84.19 84.22 84.2 M. sp M26
M. sp M26
M. sphagni DSM 44076
M. rhodesiae JS60
M. sp EPa45
M. crocinum DSM 45433
M. aromaticivorans JS19b1
M. aichiense DSM 44147
M. rhodesiae DSM 44223
C
84.62 85.34 85.56 85.74 85.75 85.8 87.91 88.51 90.99 95.42 98.12 100 M. acapulense CSURP1424
84.5 85.56 85.73 85.56 85.62 85.66 87.85 87.9 91.39 95.36 100 98.12 M. lehmannii CECT 8763
84.5 85.21 85.31 85.49 85.61 85.63 87.89 87.93 90.99 100 95.36 95.42 M. neumannii CECT 8766
84.52 85.46 85.42 85.7 85.69 85.74 88.01 88.37 100 90.99 91.39 90.99 M. novocastrense DSM 44203
84.78 85.31 85.49 85.74 85.86 85.84 89.51 100 88.37 87.93 87.9 88.51 M. malmesburyense WCM 7299
84.71 85.19 85.3 85.69 85.68 85.72 100 89.51 88.01 87.89 87.85 87.91 M. komanii GPK 1020
84.53 85.18 85.21 92.2 94.97 100 85.72 85.84 85.74 85.63 85.66 85.8 M. sp IS-3022
84.56 85.15 85.21 92.16 100 94.97 85.68 85.86 85.69 85.61 85.62 85.75 M. sp GA-0227b
84.51 85.11 85.19 100 92.16 92.2 85.69 85.74 85.7 85.49 85.56 85.74 M. celeriflavum DSM 46765
85.63 88.28 100 85.19 85.21 85.21 85.3 85.49 85.42 85.31 85.73 85.56 M. rutilum DSM 45405
85.5 100 88.28 85.11 85.15 85.18 85.19 85.31 85.46 85.21 85.56 85.34 M. flavescens DSM 43991
100 85.5 85.63 84.51 84.56 84.53 84.71 84.78 84.52 84.5 84.5 84.62 M. phlei CCUG 21000
M. phlei CCUG 21000
M. flavescens DSM 43991
M. rutilum DSM 45405
M. celeriflavum DSM 46765
M. sp GA-0227b
M. sp IS-3022
M. komanii GPK 1020
M. malmesburyense WCM 7299
M. novocastrense DSM 44203
M. neumannii CECT 8766
M. lehmannii CECT 8763
M. acapulense CSURP1424
D
83.99 83.91 84.12 84.28 84.67 86.92 88.54 100 M. hodleri DSM 44183
83.82 83.75 84.04 84.34 84.4 86 100 88.54 M. sp Root135
83.72 83.88 83.98 84.15 84.42 100 86 86.92 M. yunnanensis DSM 44838
83.99 84.01 84.09 84.1 100 84.42 84.4 84.67 M. madagascariense DSM 45167
83.87 84.52 84.87 100 84.1 84.15 84.34 84.28 M. sediminis DSM 45643
83.97 84.49 100 84.87 84.09 83.98 84.04 84.12 M. arabiense DSM 45768
83.86 100 84.49 84.52 84.01 83.88 83.75 83.91 M. grossiae GK
100 83.86 83.97 83.87 83.99 83.72 83.82 83.99 M. sp URHB0044
M. sp URHB0044
M. grossiae GK
M. arabiense DSM 45768
M. sediminis DSM 45643
M. madagascariense DSM 45167
M. yunnanensis DSM 44838
M. sp Root135
M. hodleri DSM 44183

Fig S3

E

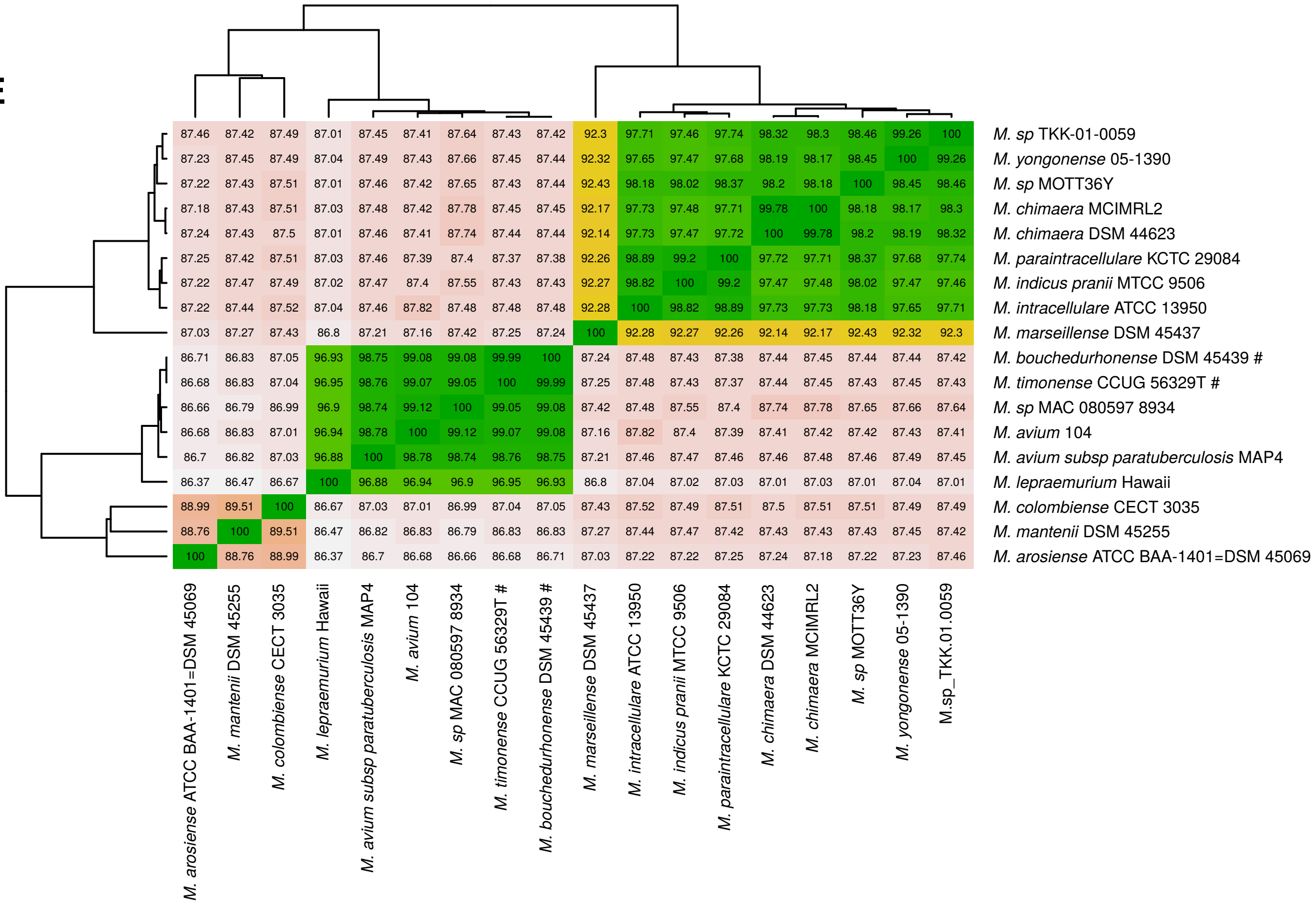
M. sp TKK-01-0059 | 87.46 87.42 87.49 87.01 87.45 87.41 87.64 87.43 87.42 92.3 97.71 97.46 97.74 98.32 98.3 98.46 99.26 100
M. yongonense 05-1390 | 87.23 87.45 87.49 87.04 87.49 87.43 87.66 87.45 87.44 92.32 97.65 97.47 97.68 98.19 98.17 98.45 100 99.26
M. sp MOTT36Y | 87.22 87.43 87.51 87.01 87.46 87.42 87.65 87.43 87.44 92.43 98.18 98.02 98.37 98.2 98.18 100 98.45 98.46
M. chimaera MCIMRL2 | 87.18 87.43 87.51 87.03 87.48 87.42 87.78 87.45 87.45 92.17 97.73 97.48 97.71 99.78 100 98.18 98.17 98.3
M. chimaera DSM 44623 | 87.24 87.43 87.5 87.01 87.46 87.41 87.74 87.44 87.44 92.14 97.73 97.47 97.72 100 99.78 98.2 98.19 98.32
M. paraintracellulare KCTC 29084 | 87.25 87.42 87.51 87.03 87.46 87.39 87.4 87.37 87.38 92.26 98.89 99.2 100 97.72 97.71 98.37 97.68 97.74
M. indicus pranii MTCC 9506 | 87.22 87.47 87.49 87.02 87.47 87.4 87.55 87.43 87.43 92.27 98.82 100 99.2 97.47 97.48 98.02 97.47 97.46
M. intracellulare ATCC 13950 | 87.22 87.44 87.52 87.04 87.46 87.82 87.48 87.48 87.48 92.28 100 98.82 98.89 97.73 97.73 98.18 97.65 97.71
M. marseillense DSM 45437 | 87.03 87.27 87.43 86.8 87.21 87.16 87.42 87.25 87.24 100 92.28 92.27 92.26 92.14 92.17 92.43 92.32 92.3
M. bouchedurhonense DSM 45439 # | 86.71 86.83 87.05 96.93 98.75 99.08 99.08 99.99 100 87.24 87.48 87.43 87.38 87.44 87.45 87.44 87.44 87.42
M. timonense CCUG 56329T # | 86.68 86.83 87.04 96.95 98.76 99.07 99.05 100 99.99 87.25 87.48 87.43 87.37 87.44 87.45 87.43 87.45 87.43
M. sp MAC 080597 8934 | 86.66 86.79 86.99 96.9 98.74 99.12 100 99.05 99.08 87.42 87.48 87.55 87.4 87.74 87.78 87.65 87.66 87.64
M. avium 104 | 86.68 86.83 87.01 96.94 98.78 100 99.12 99.07 99.08 87.16 87.82 87.4 87.39 87.41 87.42 87.42 87.43 87.41
M. avium subsp paratuberculosis MAP4 | 86.7 86.82 87.03 96.88 100 98.78 98.74 98.76 98.75 87.21 87.46 87.47 87.46 87.46 87.48 87.46 87.49 87.45
M. lepraemurium Hawaii | 86.37 86.47 86.67 100 96.88 96.94 96.9 96.95 96.93 86.8 87.04 87.02 87.03 87.01 87.03 87.01 87.04 87.01
M. colombiense CECT 3035 | 88.99 89.51 100 86.67 87.03 87.01 86.99 87.04 87.05 87.43 87.52 87.49 87.51 87.5 87.51 87.51 87.49 87.49
M. mantenii DSM 45255 | 88.76 100 89.51 86.47 86.82 86.83 86.79 86.83 86.83 87.27 87.44 87.47 87.42 87.43 87.43 87.43 87.45 87.42
M. arosiense ATCC BAA-1401=DSM 45069 | 100 88.76 88.99 86.37 86.7 86.68 86.66 86.68 86.71 87.03 87.22 87.22 87.25 87.24 87.18 87.22 87.23 87.46
M. arosiense ATCC BAA-1401=DSM 45069
M. mantenii DSM 45255
M. colombiense CECT 3035
M. lepraemurium Hawaii
M. avium subsp paratuberculosis MAP4
M. avium 104
M. sp MAC 080597 8934
M. timonense CCUG 56329T #
M. bouchedurhonense DSM 45439 #
M. marseillense DSM 45437
M. intracellulare ATCC 13950
M. indicus pranii MTCC 9506
M. paraintracellulare KCTC 29084
M. chimaera DSM 44623
M. chimaera MCIMRL2
M. sp MOTT36Y
M. yongonense 05-1390
M.sp_TKK.01.0059

F

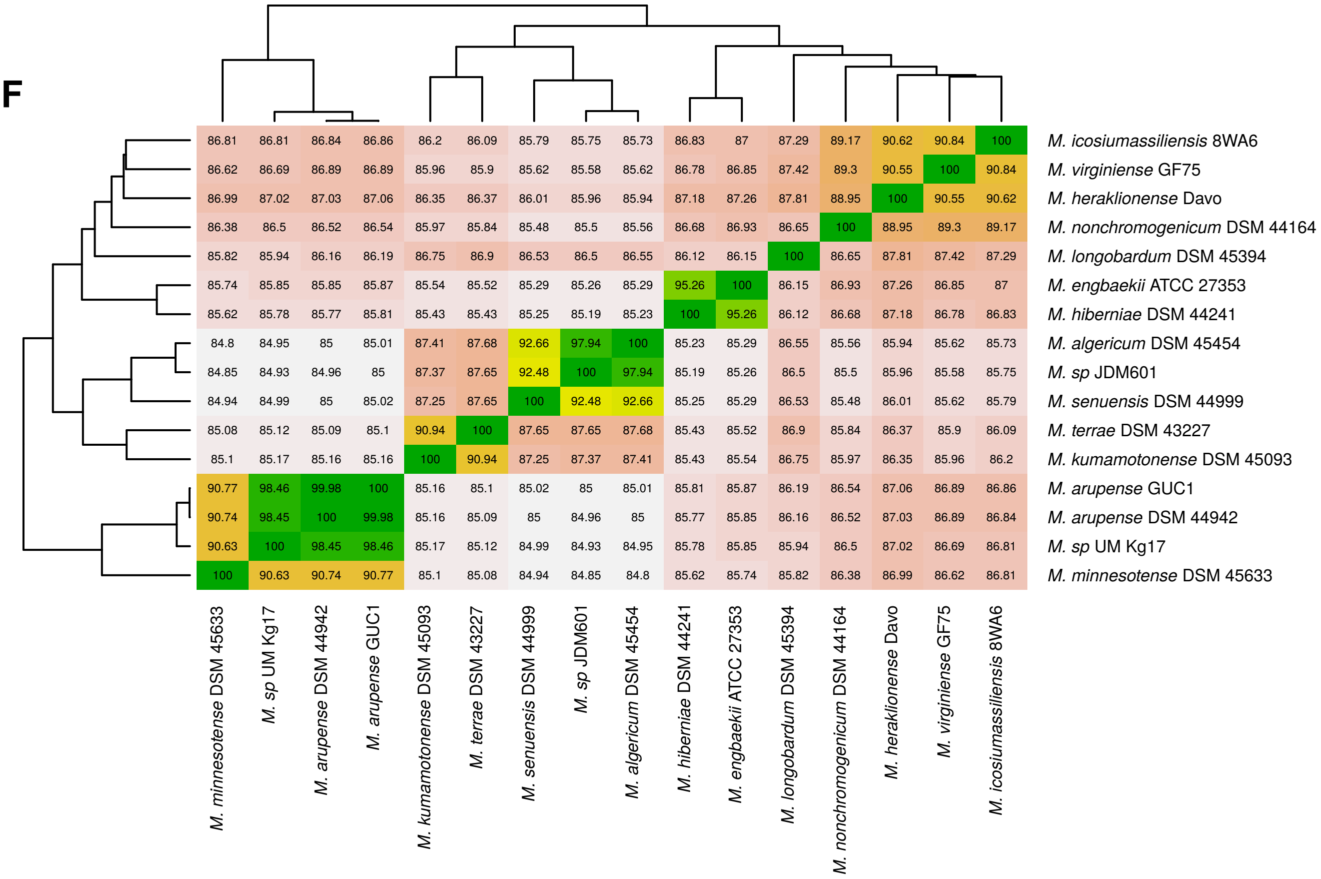
M. icosiumassiliensis 8WA6 | 86.81 86.81 86.84 86.86 86.2 86.09 85.79 85.75 85.73 86.83 87 87.29 89.17 90.62 90.84 100
M. virginiense GF75 | 86.62 86.69 86.89 86.89 85.96 85.9 85.62 85.58 85.62 86.78 86.85 87.42 89.3 90.55 100 90.84
M. heraklionense Davo | 86.99 87.02 87.03 87.06 86.35 86.37 86.01 85.96 85.94 87.18 87.26 87.81 88.95 100 90.55 90.62
M. nonchromogenicum DSM 44164 | 86.38 86.5 86.52 86.54 85.97 85.84 85.48 85.5 85.56 86.68 86.93 86.65 100 88.95 89.3 89.17
M. longobardum DSM 45394 | 85.82 85.94 86.16 86.19 86.75 86.9 86.53 86.5 86.55 86.12 86.15 100 86.65 87.81 87.42 87.29
M. engbaekii ATCC 27353 | 85.74 85.85 85.85 85.87 85.54 85.52 85.29 85.26 85.29 95.26 100 86.15 86.93 87.26 86.85 87
M. hiberniae DSM 44241 | 85.62 85.78 85.77 85.81 85.43 85.43 85.25 85.19 85.23 100 95.26 86.12 86.68 87.18 86.78 86.83
M. algericum DSM 45454 | 84.8 84.95 85 85.01 87.41 87.68 92.66 97.94 100 85.23 85.29 86.55 85.56 85.94 85.62 85.73
M. sp JDM601 | 84.85 84.93 84.96 85 87.37 87.65 92.48 100 97.94 85.19 85.26 86.5 85.5 85.96 85.58 85.75
M. senuensis DSM 44999 | 84.94 84.99 85 85.02 87.25 87.65 100 92.48 92.66 85.25 85.29 86.53 85.48 86.01 85.62 85.79
M. terrae DSM 43227 | 85.08 85.12 85.09 85.1 90.94 100 87.65 87.65 87.68 85.43 85.52 86.9 85.84 86.37 85.9 86.09
M. kumamotonense DSM 45093 | 85.1 85.17 85.16 85.16 100 90.94 87.25 87.37 87.41 85.43 85.54 86.75 85.97 86.35 85.96 86.2
M. arupense GUC1 | 90.77 98.46 99.98 100 85.16 85.1 85.02 85 85.01 85.81 85.87 86.19 86.54 87.06 86.89 86.86
M. arupense DSM 44942 | 90.74 98.45 100 99.98 85.16 85.09 85 84.96 85 85.77 85.85 86.16 86.52 87.03 86.89 86.84
M. sp UM Kg17 | 90.63 100 98.45 98.46 85.17 85.12 84.99 84.93 84.95 85.78 85.85 85.94 86.5 87.02 86.69 86.81
M. minnesotense DSM 45633 | 100 90.63 90.74 90.77 85.1 85.08 84.94 84.85 84.8 85.62 85.74 85.82 86.38 86.99 86.62 86.81
M. minnesotense DSM 45633
M. sp UM Kg17
M. arupense DSM 44942
M. arupense GUC1
M. kumamotonense DSM 45093
M. terrae DSM 43227
M. senuensis DSM 44999
M. sp JDM601
M. algericum DSM 45454
M. hiberniae DSM 44241
M. engbaekii ATCC 27353
M. longobardum DSM 45394
M. nonchromogenicum DSM 44164
M. heraklionense Davo
M. virginiense GF75
M. icosiumassiliensis 8WA6

Fig S3

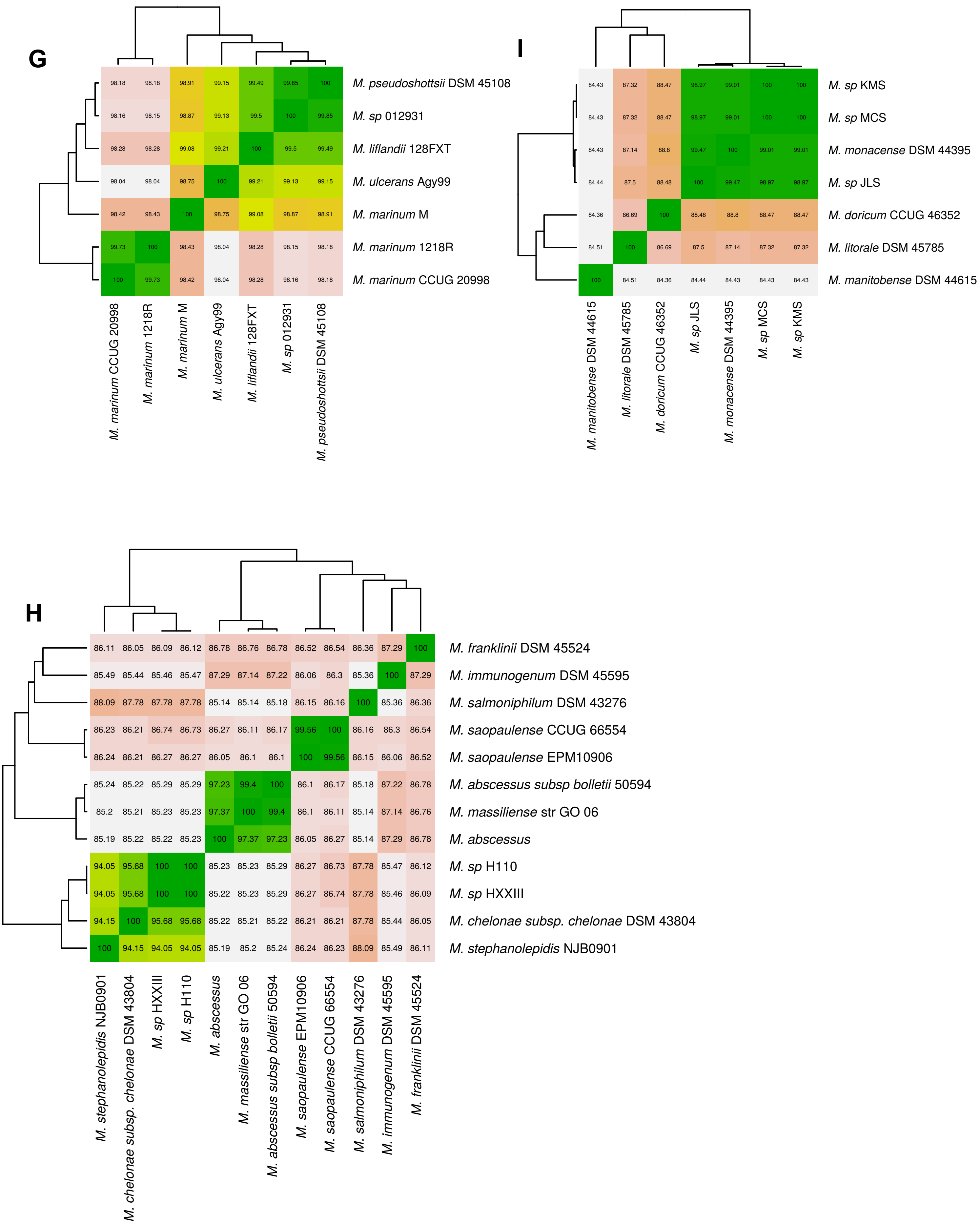
G
98.18 98.18 98.91 99.15 99.49 99.85 100 M. pseudoshottsii DSM 45108
98.16 98.15 98.87 99.13 99.5 100 99.85 M. sp 012931
98.28 98.28 99.08 99.21 100 99.5 99.49 M. liflandii 128FXT
98.04 98.04 98.75 100 99.21 99.13 99.15 M. ulcerans Agy99
98.42 98.43 100 98.75 99.08 98.87 98.91 M. marinum M
99.73 100 98.43 98.04 98.28 98.15 98.18 M. marinum 1218R
100 99.73 98.42 98.04 98.28 98.16 98.18 M. marinum CCUG 20998
M. marinum CCUG 20998
M. marinum 1218R
M. marinum M
M. ulcerans Agy99
M. liflandii 128FXT
M. sp 012931
M. pseudoshottsii DSM 45108
I
84.43 87.32 88.47 98.97 99.01 100 100 M. sp KMS
84.43 87.32 88.47 98.97 99.01 100 100 M. sp MCS
84.43 87.14 88.8 99.47 100 99.01 99.01 M. monacense DSM 44395
84.44 87.5 88.48 100 99.47 98.97 98.97 M. sp JLS
84.36 86.69 100 88.48 88.8 88.47 88.47 M. doricum CCUG 46352
84.51 100 86.69 87.5 87.14 87.32 87.32 M. litorale DSM 45785
100 84.51 84.36 84.44 84.43 84.43 84.43 M. manitobense DSM 44615
M. manitobense DSM 44615
M. litorale DSM 45785
M. doricum CCUG 46352
M. sp JLS
M. monacense DSM 44395
M. sp MCS
M. sp KMS
H
86.11 86.05 86.09 86.12 86.78 86.76 86.78 86.52 86.54 86.36 87.29 100 M. franklinii DSM 45524
85.49 85.44 85.46 85.47 87.29 87.14 87.22 86.06 86.3 85.36 100 87.29 M. immunogenum DSM 45595
88.09 87.78 87.78 87.78 85.14 85.14 85.18 86.15 86.16 100 85.36 86.36 M. salmoniphilum DSM 43276
86.23 86.21 86.74 86.73 86.27 86.11 86.17 99.56 100 86.16 86.3 86.54 M. saopaulense CCUG 66554
86.24 86.21 86.27 86.27 86.05 86.1 86.1 100 99.56 86.15 86.06 86.52 M. saopaulense EPM10906
85.24 85.22 85.29 85.29 97.23 99.4 100 86.1 86.17 85.18 87.22 86.78 M. abscessus subsp bolletii 50594
85.2 85.21 85.23 85.23 97.37 100 99.4 86.1 86.11 85.14 87.14 86.76 M. massiliense str GO 06
85.19 85.22 85.22 85.23 100 97.37 97.23 86.05 86.27 85.14 87.29 86.78 M. abscessus
94.05 95.68 100 100 85.23 85.23 85.29 86.27 86.73 87.78 85.47 86.12 M. sp H110
94.05 95.68 100 100 85.22 85.23 85.29 86.27 86.74 87.78 85.46 86.09 M. sp HXXIII
94.15 100 95.68 95.68 85.22 85.21 85.22 86.21 86.21 87.78 85.44 86.05 M. chelonae subsp. chelonae DSM 43804
100 94.15 94.05 94.05 85.19 85.2 85.24 86.24 86.23 88.09 85.49 86.11 M. stephanolepidis NJB0901
M. stephanolepidis NJB0901
M. chelonae subsp. chelonae DSM 43804
M. sp HXXIII
M. sp H110
M. abscessus
M. massiliense str GO 06
M. abscessus subsp bolletii 50594
M. saopaulense EPM10906
M. saopaulense CCUG 66554
M. salmoniphilum DSM 43276
M. immunogenum DSM 45595
M. franklinii DSM 45524

Fig S3

J

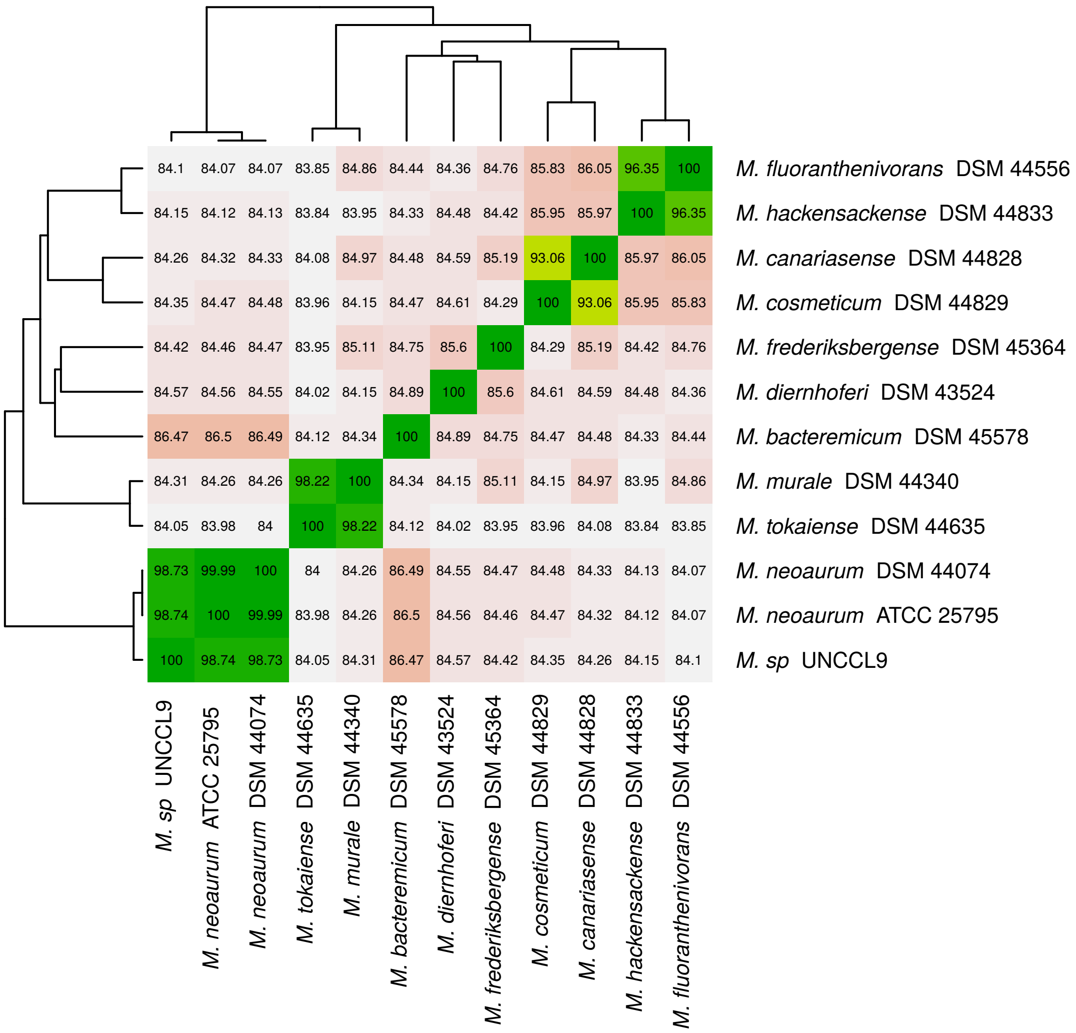
84.1 84.07 84.07 83.85 84.86 84.44 84.36 84.76 85.83 86.05 96.35 100 M. fluoranthenivorans DSM 44556
84.15 84.12 84.13 83.84 83.95 84.33 84.48 84.42 85.95 85.97 100 96.35 M. hackensackense DSM 44833
84.26 84.32 84.33 84.08 84.97 84.48 84.59 85.19 93.06 100 85.97 86.05 M. canariasense DSM 44828
84.35 84.47 84.48 83.96 84.15 84.47 84.61 84.29 100 93.06 85.95 85.83 M. cosmeticum DSM 44829
84.42 84.46 84.47 83.95 85.11 84.75 85.6 100 84.29 85.19 84.42 84.76 M. frederiksbergense DSM 45364
84.57 84.56 84.55 84.02 84.15 84.89 100 85.6 84.61 84.59 84.48 84.36 M. diernhoferi DSM 43524
86.47 86.5 86.49 84.12 84.34 100 84.89 84.75 84.47 84.48 84.33 84.44 M. bacteremicum DSM 45578
84.31 84.26 84.26 98.22 100 84.34 84.15 85.11 84.15 84.97 83.95 84.86 M. murale DSM 44340
84.05 83.98 84 100 98.22 84.12 84.02 83.95 83.96 84.08 83.84 83.85 M. tokaiense DSM 44635
98.73 99.99 100 84 84.26 86.49 84.55 84.47 84.48 84.33 84.13 84.07 M. neoaurum DSM 44074
98.74 100 99.99 83.98 84.26 86.5 84.56 84.46 84.47 84.32 84.12 84.07 M. neoaurum ATCC 25795
100 98.74 98.73 84.05 84.31 86.47 84.57 84.42 84.35 84.26 84.15 84.1 M. sp UNCCL9
M. sp UNCCL9
M. neoaurum ATCC 25795
M. neoaurum DSM 44074
M. tokaiense DSM 44635
M. murale DSM 44340
M. bacteremicum DSM 45578
M. diernhoferi DSM 43524
M. frederiksbergense DSM 45364
M. cosmeticum DSM 44829
M. canariasense DSM 44828
M. hackensackense DSM 44833
M. fluoranthenivorans DSM 44556

K

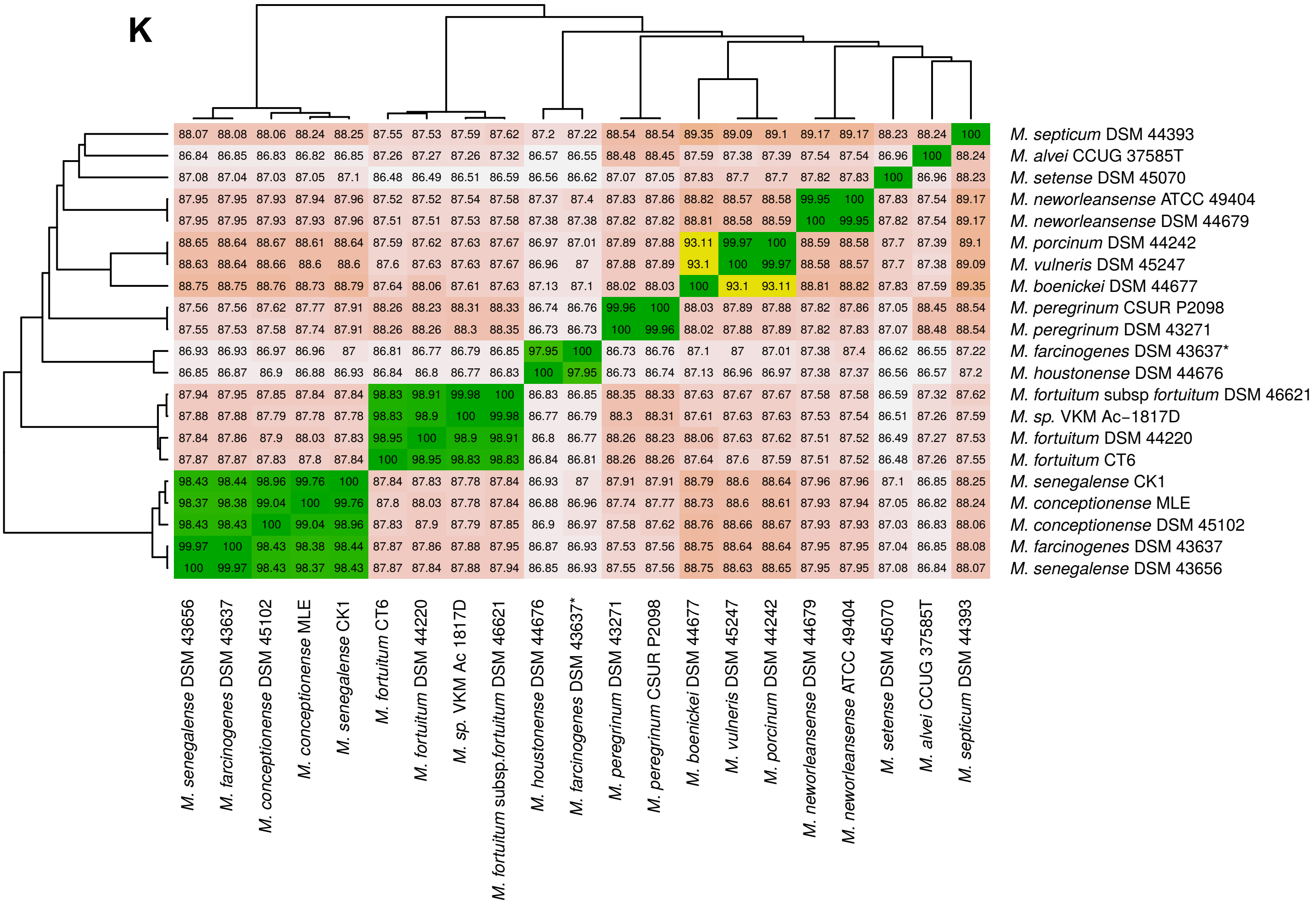
88.07 88.08 88.06 88.24 88.25 87.55 87.53 87.59 87.62 87.2 87.22 88.54 88.54 89.35 89.09 89.1 89.17 89.17 88.23 88.24 100 M. septicum DSM 44393
86.84 86.85 86.83 86.82 86.85 87.26 87.27 87.26 87.32 86.57 86.55 88.48 88.45 87.59 87.38 87.39 87.54 87.54 86.96 100 88.24 M. alvei CCUG 37585T
87.08 87.04 87.03 87.05 87.1 86.48 86.49 86.51 86.59 86.56 86.62 87.07 87.05 87.83 87.7 87.7 87.82 87.83 100 86.96 88.23 M. setense DSM 45070
87.95 87.95 87.93 87.94 87.96 87.52 87.52 87.54 87.58 87.37 87.4 87.83 87.86 88.82 88.57 88.58 99.95 100 87.83 87.54 89.17 M. neworleansense ATCC 49404
87.95 87.95 87.93 87.93 87.96 87.51 87.51 87.53 87.58 87.38 87.38 87.82 87.82 88.81 88.58 88.59 100 99.95 87.82 87.54 89.17 M. neworleansense DSM 44679
88.65 88.64 88.67 88.61 88.64 87.59 87.62 87.63 87.67 86.97 87.01 87.89 87.88 93.11 99.97 100 88.59 88.58 87.7 87.39 89.1 M. porcinum DSM 44242
88.63 88.64 88.66 88.6 88.6 87.6 87.63 87.63 87.67 86.96 87 87.88 87.89 93.1 100 99.97 88.58 88.57 87.7 87.38 89.09 M. vulneris DSM 45247
88.75 88.75 88.76 88.73 88.79 87.64 88.06 87.61 87.63 87.13 87.1 88.02 88.03 100 93.1 93.11 88.81 88.82 87.83 87.59 89.35 M. boenickei DSM 44677
87.56 87.56 87.62 87.77 87.91 88.26 88.23 88.31 88.33 86.74 86.76 99.96 100 88.03 87.89 87.88 87.82 87.86 87.05 88.45 88.54 M. peregrinum CSUR P2098
87.55 87.53 87.58 87.74 87.91 88.26 88.26 88.3 88.35 86.73 86.73 100 99.96 88.02 87.88 87.89 87.82 87.83 87.07 88.48 88.54 M. peregrinum DSM 43271
86.93 86.93 86.97 86.96 87 86.81 86.77 86.79 86.85 97.95 100 86.73 86.76 87.1 87 87.01 87.38 87.4 86.62 86.55 87.22 M. farcinogenes DSM 43637*
86.85 86.87 86.9 86.88 86.93 86.84 86.8 86.77 86.83 100 97.95 86.73 86.74 87.13 86.96 86.97 87.38 87.37 86.56 86.57 87.2 M. houstonense DSM 44676
87.94 87.95 87.85 87.84 87.84 98.83 98.91 99.98 100 86.83 86.85 88.35 88.33 87.63 87.67 87.67 87.58 87.58 86.59 87.32 87.62 M. fortuitum subsp fortuitum DSM 46621
87.88 87.88 87.79 87.78 87.78 98.83 98.9 100 99.98 86.77 86.79 88.3 88.31 87.61 87.63 87.63 87.53 87.54 86.51 87.26 87.59 M. sp. VKM Ac−1817D
87.84 87.86 87.9 88.03 87.83 98.95 100 98.9 98.91 86.8 86.77 88.26 88.23 88.06 87.63 87.62 87.51 87.52 86.49 87.27 87.53 M. fortuitum DSM 44220
87.87 87.87 87.83 87.8 87.84 100 98.95 98.83 98.83 86.84 86.81 88.26 88.26 87.64 87.6 87.59 87.51 87.52 86.48 87.26 87.55 M. fortuitum CT6
98.43 98.44 98.96 99.76 100 87.84 87.83 87.78 87.84 86.93 87 87.91 87.91 88.79 88.6 88.64 87.96 87.96 87.1 86.85 88.25 M. senegalense CK1
98.37 98.38 99.04 100 99.76 87.8 88.03 87.78 87.84 86.88 86.96 87.74 87.77 88.73 88.6 88.61 87.93 87.94 87.05 86.82 88.24 M. conceptionense MLE
98.43 98.43 100 99.04 98.96 87.83 87.9 87.79 87.85 86.9 86.97 87.58 87.62 88.76 88.66 88.67 87.93 87.93 87.03 86.83 88.06 M. conceptionense DSM 45102
99.97 100 98.43 98.38 98.44 87.87 87.86 87.88 87.95 86.87 86.93 87.53 87.56 88.75 88.64 88.64 87.95 87.95 87.04 86.85 88.08 M. farcinogenes DSM 43637
100 99.97 98.43 98.37 98.43 87.87 87.84 87.88 87.94 86.85 86.93 87.55 87.56 88.75 88.63 88.65 87.95 87.95 87.08 86.84 88.07 M. senegalense DSM 43656
M. senegalense DSM 43656
M. farcinogenes DSM 43637
M. conceptionense DSM 45102
M. conceptionense MLE
M. senegalense CK1
M. fortuitum CT6
M. fortuitum DSM 44220
M. sp. VKM Ac 1817D
M. fortuitum subsp.fortuitum DSM 46621
M. houstonense DSM 44676
M. farcinogenes DSM 43637*
M. peregrinum DSM 43271
M. peregrinum CSUR P2098
M. boenickei DSM 44677
M. vulneris DSM 45247
M. porcinum DSM 44242
M. neworleansense DSM 44679
M. neworleansense ATCC 49404
M. setense DSM 45070
M. alvei CCUG 37585T
M. septicum DSM 44393

L

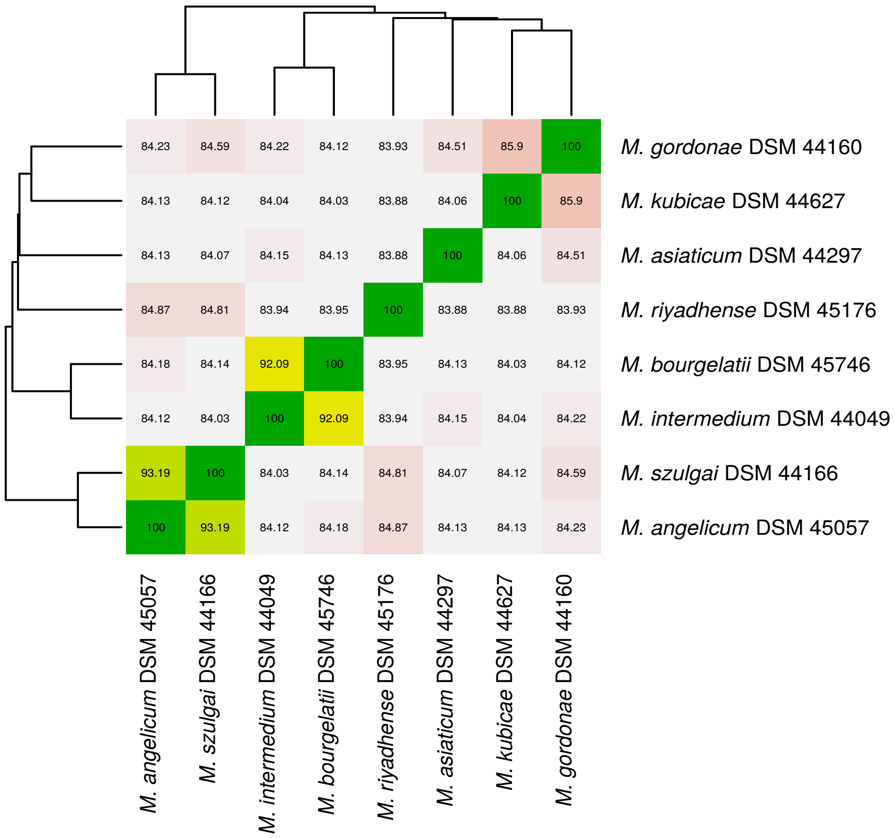
84.23 84.59 84.22 84.12 83.93 84.51 85.9 100 M. gordonae DSM 44160
84.13 84.12 84.04 84.03 83.88 84.06 100 85.9 M. kubicae DSM 44627
84.13 84.07 84.15 84.13 83.88 100 84.06 84.51 M. asiaticum DSM 44297
84.87 84.81 83.94 83.95 100 83.88 83.88 83.93 M. riyadhense DSM 45176
84.18 84.14 92.09 100 83.95 84.13 84.03 84.12 M. bourgelatii DSM 45746
84.12 84.03 100 92.09 83.94 84.15 84.04 84.22 M. intermedium DSM 44049
93.19 100 84.03 84.14 84.81 84.07 84.12 84.59 M. szulgai DSM 44166
100 93.19 84.12 84.18 84.87 84.13 84.13 84.23 M. angelicum DSM 45057
M. angelicum DSM 45057
M. szulgai DSM 44166
M. intermedium DSM 44049
M. bourgelatii DSM 45746
M. riyadhense DSM 45176
M. asiaticum DSM 44297
M. kubicae DSM 44627
M. gordonae DSM 44160

M

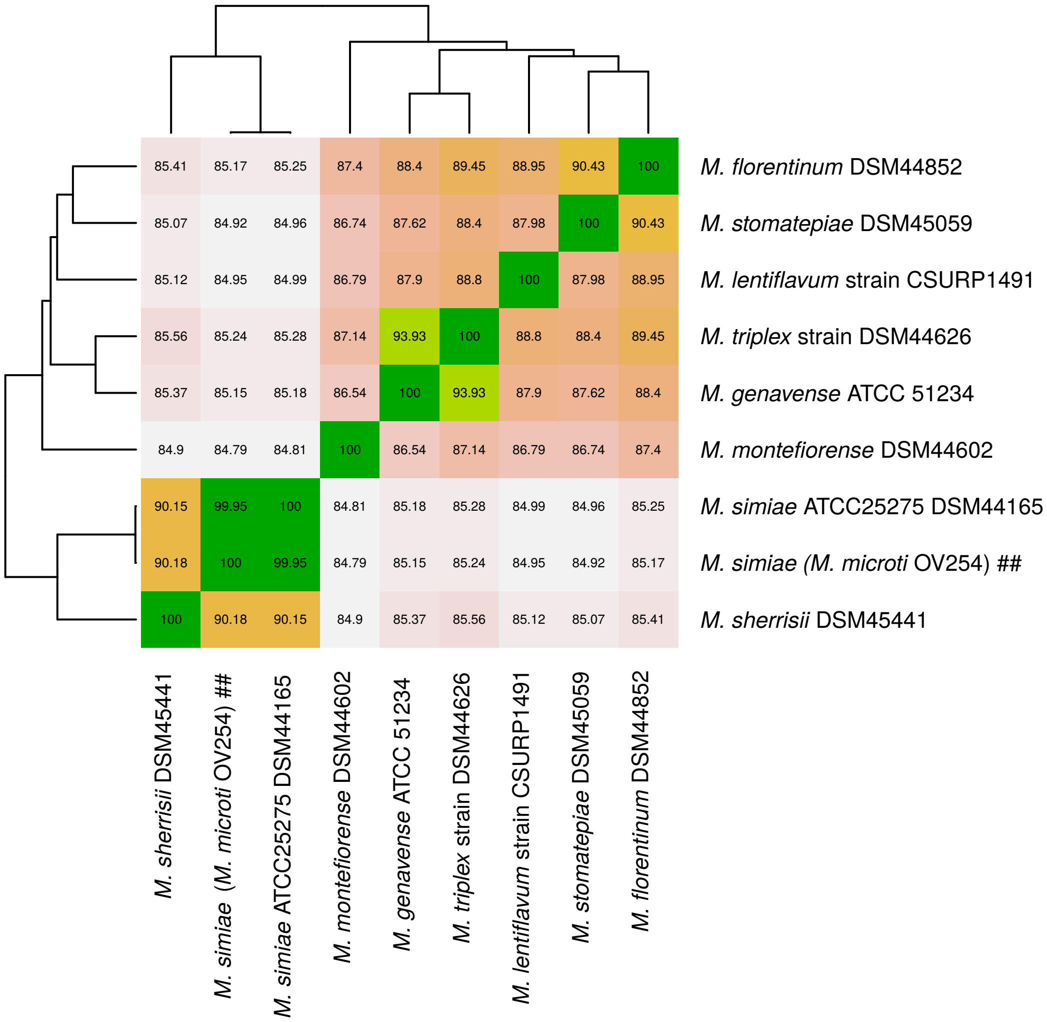
85.41 85.17 85.25 87.4 88.4 89.45 88.95 90.43 100 M. florentinum DSM44852
85.07 84.92 84.96 86.74 87.62 88.4 87.98 100 90.43 M. stomatepiae DSM45059
85.12 84.95 84.99 86.79 87.9 88.8 100 87.98 88.95 M. lentiflavum strain CSURP1491
85.56 85.24 85.28 87.14 93.93 100 88.8 88.4 89.45 M. triplex strain DSM44626
85.37 85.15 85.18 86.54 100 93.93 87.9 87.62 88.4 M. genavense ATCC 51234
84.9 84.79 84.81 100 86.54 87.14 86.79 86.74 87.4 M. montefiorense DSM44602
90.15 99.95 100 84.81 85.18 85.28 84.99 84.96 85.25 M. simiae ATCC25275 DSM44165
90.18 100 99.95 84.79 85.15 85.24 84.95 84.92 85.17 M. simiae (M. microti OV254) ##
100 90.18 90.15 84.9 85.37 85.56 85.12 85.07 85.41 M. sherrisii DSM45441
M. sherrisii DSM45441
M. simiae (M. microti OV254) ##
M. simiae ATCC25275 DSM44165
M. montefiorense DSM44602
M. genavense ATCC 51234
M. triplex strain DSM44626
M. lentiflavum strain CSURP1491
M. stomatepiae DSM45059
M. florentinum DSM44852

Fig S3

N

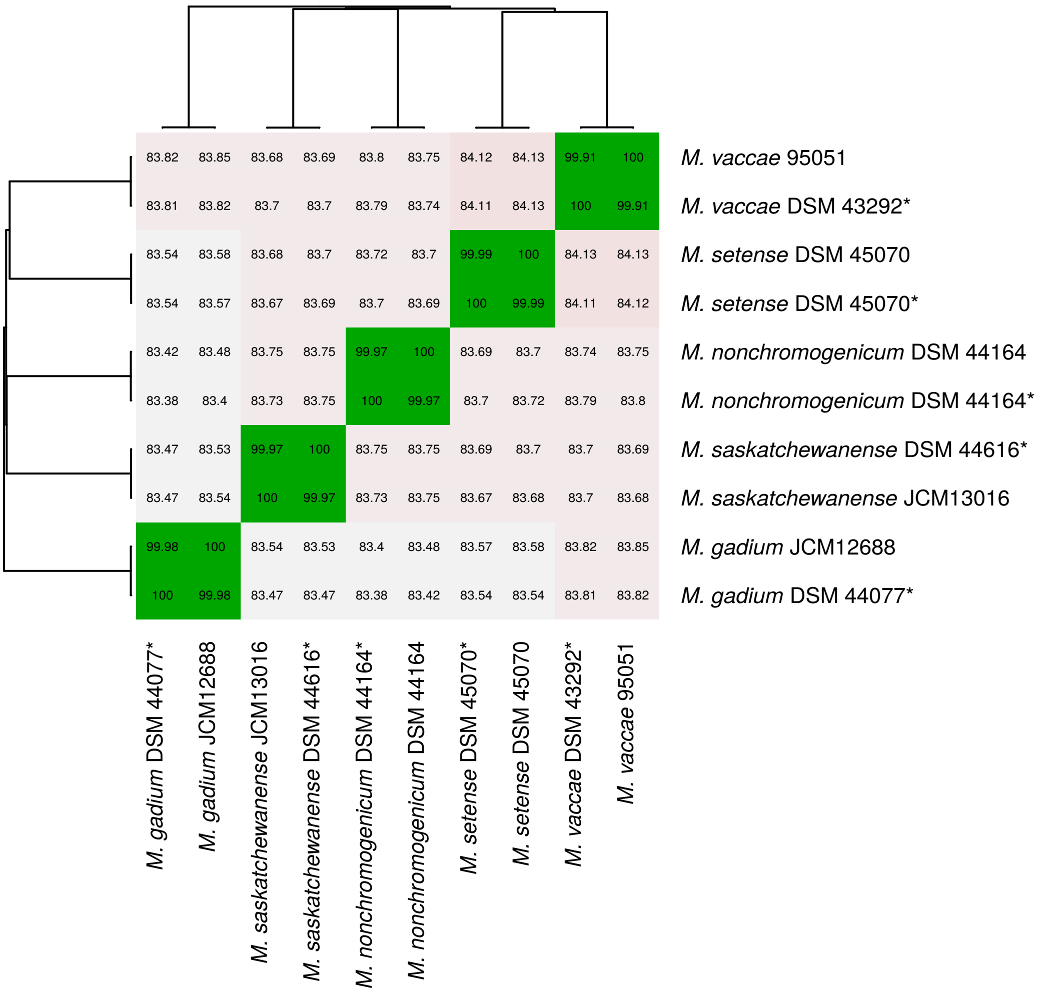
83.82 83.85 83.68 83.69 83.8 83.75 84.12 84.13 99.91 100 M. vaccae 95051
83.81 83.82 83.7 83.7 83.79 83.74 84.11 84.13 100 99.91 M. vaccae DSM 43292*
83.54 83.58 83.68 83.7 83.72 83.7 99.99 100 84.13 84.13 M. setense DSM 45070
83.54 83.57 83.67 83.69 83.7 83.69 100 99.99 84.11 84.12 M. setense DSM 45070*
83.42 83.48 83.75 83.75 99.97 100 83.69 83.7 83.74 83.75 M. nonchromogenicum DSM 44164
83.38 83.4 83.73 83.75 100 99.97 83.7 83.72 83.79 83.8 M. nonchromogenicum DSM 44164*
83.47 83.53 99.97 100 83.75 83.75 83.69 83.7 83.7 83.69 M. saskatchewanense DSM 44616*
83.47 83.54 100 99.97 83.73 83.75 83.67 83.68 83.7 83.68 M. saskatchewanense JCM13016
99.98 100 83.54 83.53 83.4 83.48 83.57 83.58 83.82 83.85 M. gadium JCM12688
100 99.98 83.47 83.47 83.38 83.42 83.54 83.54 83.81 83.82 M. gadium DSM 44077*
M. gadium DSM 44077*
M. gadium JCM12688
M. saskatchewanense JCM13016
M. saskatchewanense DSM 44616*
M. nonchromogenicum DSM 44164*
M. nonchromogenicum DSM 44164
M. setense DSM 45070*
M. setense DSM 45070
M. vaccae DSM 43292*
M. vaccae 95051

Figure S4 Heat map showing similarities of IS-element types

The pairwise "IS-similarity score" between the types of IS elements present or absent in each pair of species (strains) was calculated as described in Supplementary methods and plotted as a heat map sorted according to the "387 core gene" phylogenetic tree (see Fig 2). The similarity score is presented as a color range (indicated at the top of the heat map) from red (low), black (intermediate) to green (high). A cluster/square of green color indicates a group of species with a highly conserved degree of IS-element.

Fig S4

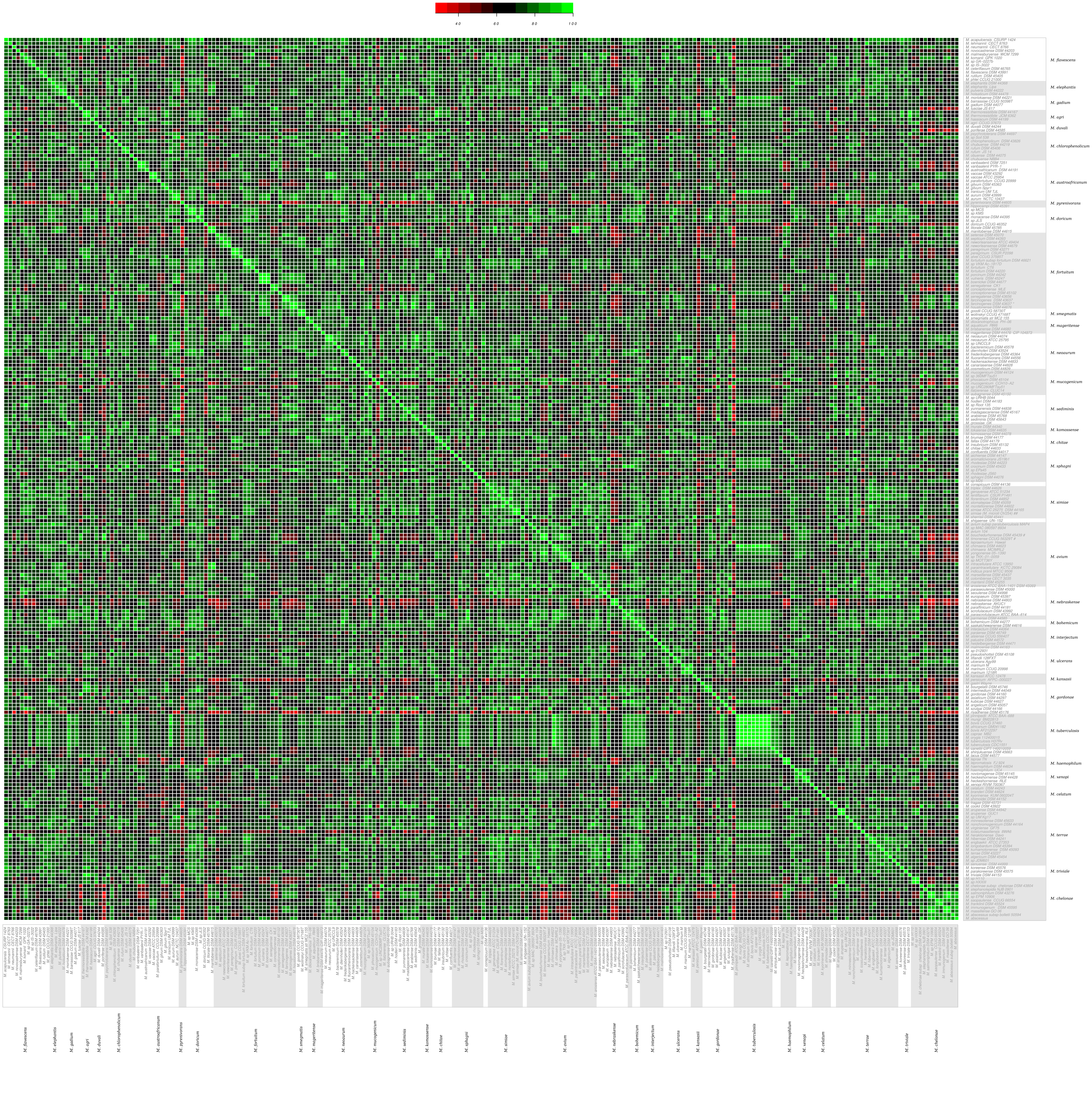
40
60
80
100
M. flavescens
M. elephantis
M. gadium
M. agri
M. duvali
M. chlorophenolicum
M. austroafricanum
M. pyrenivorans
M. doricum
M. fortuitum
M. smegmatis
M. mageritense
M. neoaurum
M. mucogenicum
M. sediminis
M. komossense
M. chitae
M. sphagni
M. simiae
M. avium
M. nebraskense
M. bohemicum
M. interjectum
M. ulcerans
M. kansasii
M. gordonae
M. tuberculosis
M. haemophilum
M. xenopi
M. celatum
M. terrae
M. triviale
M. chelonae

Figure S5 Genomic locations of tRNA genes and core ncRNA present in 47 mycobacteria

(A) The heat map plot represents the genome-wide distribution comparison of 47 tRNA genes in *M. chelonae* subsp. *chelonae* DSM 43804 (*Mchel* DSM 43804; used as reference) and their genomic location in relation to the other mycobacteria (47 genomes). The left rectangular box represents a brief color-coded figure legend explanation. The start position was *dnaA* and ended with *rpmH*, as indicated on the left-hand side. *Mchel* DSM 43804 tRNA operons/genes are represented with alternative colors (blue and pink) on the right. Boxes lacking a number correspond to single tRNA genes; those with numbers indicate the number of tRNA genes in the respective operon. The X-axis shows the mycobacterial species and SGM (orange), RGM (green; see also main Fig 2), no extractable information about growth rate (black). The core phylogenetic tree for the 47 selected mycobacteria (n = 387 core genes; see main text) is shown on the top (for details see methods section). Green boxes represent tRNA genes located at the same relative position as in the reference, *Mchel* DSM 43804. Gray boxes indicated tRNA genes that have undergone re-arrangements relative to *Mchel* DSM 43804. The numbers in the green and gray boxes correspond to the relative position of the tRNA gene in that particular species/genome. Dark green boxes indicate that the corresponding tRNA gene orthologs were not detected in these mycobacteria. The tRNA isoacceptors (y-axis) are shown on the right and red indicates tRNA genes located in regions in *M. tuberculosis* H37Rv identified as necessary for optimal growth[37]. At the bottom, the clades to which the selected mycobacteria belong are shown.

(B) The heat map plot represents the genome-wide distribution comparison of 12 core ncRNA genes in *M. chelonae* subsp. *chelonae* DSM 43804 (*Mchel* DSM 43804; used as reference) and their genomic location in relation to the other mycobacteria (47 genomes). The data are presented and color coded as in Fig S5a.

Fig S5

A

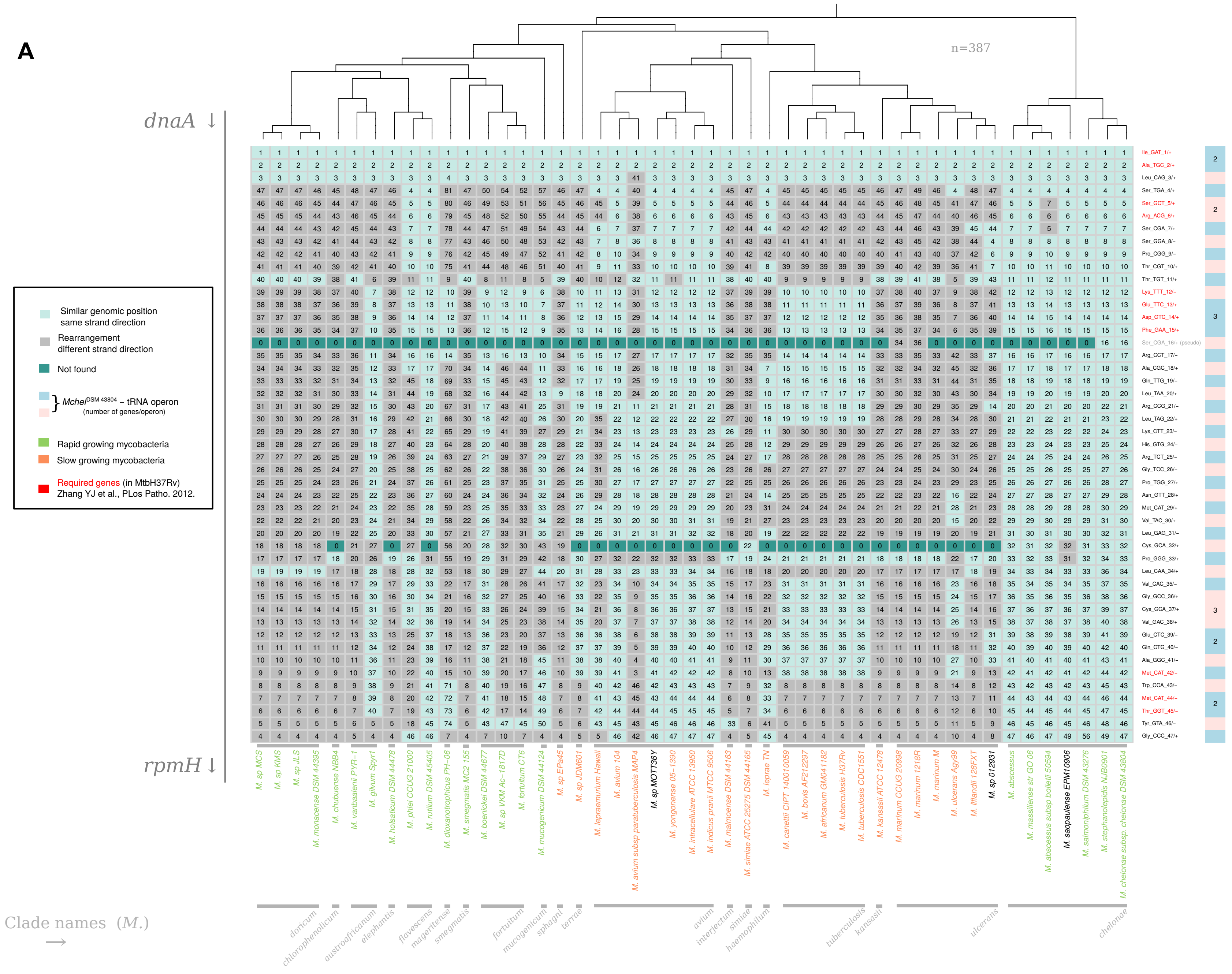
n=387
dnaA ↓
rpmH ↓
Similar genomic position same strand direction
Rearrangement different strand direction
Not found
Mchel DSM 43804 – tRNA operon (number of genes/operon)
Rapid growing mycobacteria
Slow growing mycobacteria
Required genes (in MtbH37Rv) Zhang YJ et al., PLos Patho. 2012.
Clade names (M.)
M. sp MCS
M. sp KMS
M. sp JLS
M. moriokaense DSM 44395
M. chubuense NBB4
M. vanbaalenii PYR-1
M. gilvum Spyr1
M. holsaticum DSM 44478
M. phlei CCUG 21000
M. rufum DSM 45405
M. dioxanotrophicus PH-06
M. smegmatis MC2 155
M. boenickei DSM 44677
M. sp VKM Ac-1817D
M. fortuitum CT6
M. mucogenicum DSM 44124
M. sp EPa45
M. sp JDM601
M. lepraemurium Hawaii
M. avium 104
M. avium subsp paratuberculosis MAP4
M. sp MOTT36Y
M. yongonense 05-1390
M. intracellulare ATCC 13950
M. indicus pranii MTCC 9506
M. malmoense DSM 44163
M. simiae ATCC 25275 DSM 44165
M. leprae TN
M. canettii CIPT 140010059
M. bovis AF2122/97
M. africanum GM041182
M. tuberculosis H37Rv
M. tuberculosis CDC1551
M. kansasii ATCC 12478
M. marinum CCUG 20998
M. marinum 1218R
M. marinum M
M. ulcerans Agy99
M. liflandii 128FXT
M. sp 012931
M. abscessus
M. massiliense str GO 06
M. abscessus subsp bolletii 50594
M. saopaulense EPM10906
M. salmoniphilum DSM 43276
M. stephanolepidis NJB0901
M. chelonae subsp. chelonae DSM 43804
doricum
chlorophenolicum
austroafricanum
elephantis
flavescens
mageritense
smegmatis
fortuitum
mucogenicum
sphagni
terrae
avium
interjectum
simiae
haemophilum
tuberculosis
kansasii
ulcerans
chelonae

Fig S5

B

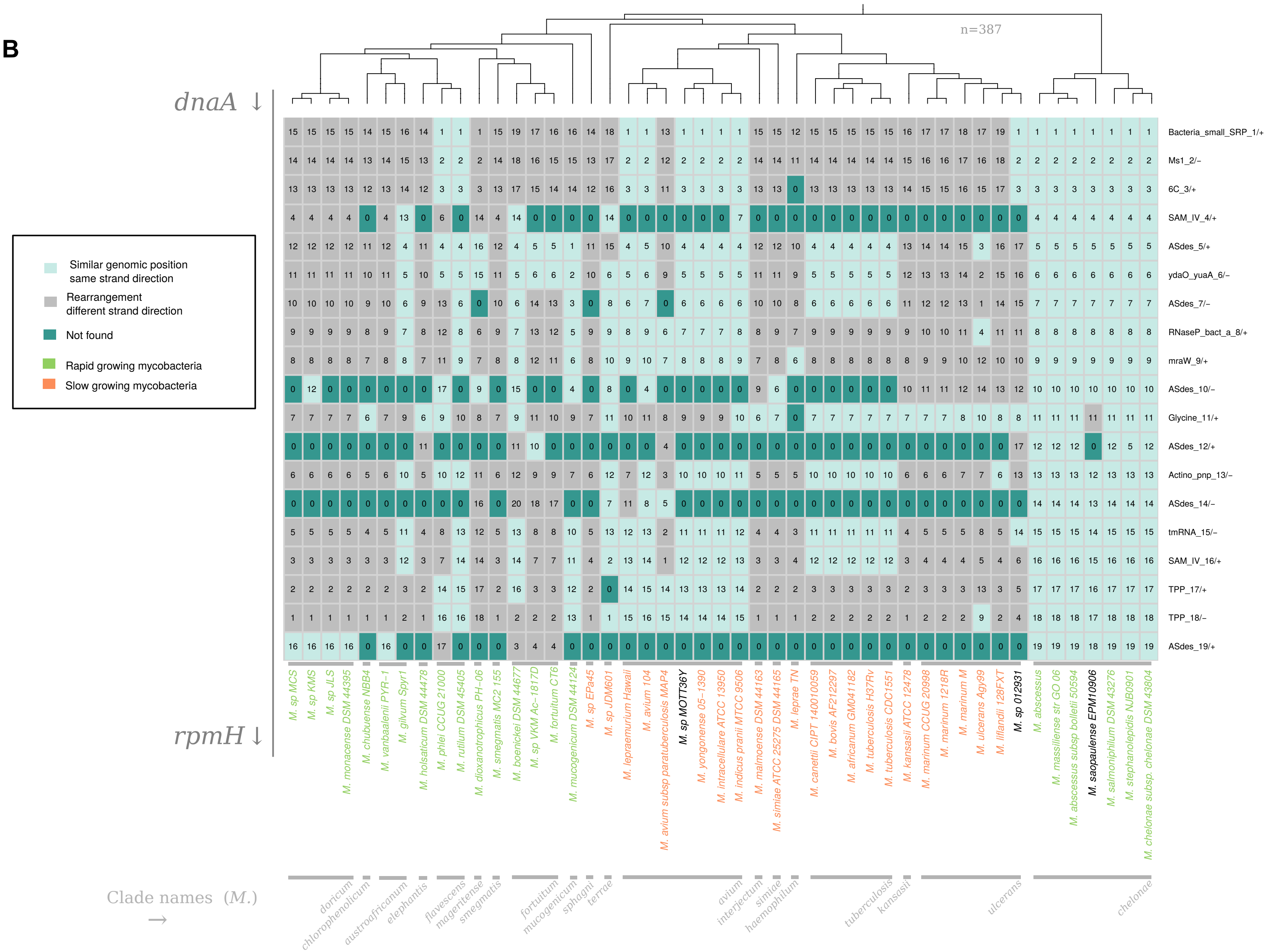
dnaA ↓
n=387
Similar genomic position
same strand direction
Rearrangement
different strand direction
Not found
Rapid growing mycobacteria
Slow growing mycobacteria
Bacteria_small_SRP_1/+
Ms1_2/–
6C_3/+
SAM_IV_4/+
ASdes_5/+
ydaO_yuaA_6/–
ASdes_7/–
RNaseP_bact_a_8/+
mraW_9/+
ASdes_10/–
Glycine_11/+
ASdes_12/+
Actino_pnp_13/–
ASdes_14/–
tmRNA_15/–
SAM_IV_16/+
TPP_17/+
TPP_18/–
ASdes_19/+
M. sp MCS
M. sp KMS
M. sp JLS
M. monacense DSM 44395
M. chubuense NBB4
M. vanbaalenii PYR-1
M. gilvum Spyr1
M. holsaticum DSM 44478
M. phlei CCUG 21000
M. rufilum DSM 45405
M. dioxanotrophicus PH-06
M. smegmatis MC2 155
M. boenickei DSM 44677
M. sp VKM Ac-1817D
M. fortuitum CT6
M. mucogenicum DSM 44124
M. sp EPa45
M. sp JDM601
M. lepraemurium Hawaii
M. avium 104
M. avium subsp paratuberculosis MAP4
M. sp MOTT36Y
M. yongonense 05-1390
M. intracellulare ATCC 13950
M. indicus pranii MTCC 9506
M. malmoense DSM 44163
M. simiae ATCC 25275 DSM 44165
M. leprae TN
M. canettii CIPT 140010059
M. bovis AF2122/97
M. africanum GM041182
M. tuberculosis H37Rv
M. tuberculosis CDC1551
M. kansasii ATCC 12478
M. marinum CCUG 20998
M. marinum 1218R
M. marinum M
M. ulcerans Agy99
M. liflandii 128FXT
M. sp 012931
M. abscessus
M. massiliense str GO 06
M. abscessus subsp bolletii 50594
M. saopaulense EPM10906
M. salmoniphilum DSM 43276
M. stephanolepidis NJB0901
M. chelonae subsp. chelonae DSM 43804
rpmH ↓
Clade names (M.) →
doricum
chlorophenolicum
austroafricanum
elephantis
flavescens
mageritense
smegmatis
fortuitum
mucogenicum
sphagni
terrae
avium
interjectum
simiae
haemophilum
tuberculosis
kansasii
ulcerans
chelonae

Figure S6 Distribution of *M. tuberculosis* candidate non-coding RNAs in mycobacteria

Homologs to ncRNAs identified in *M. tuberculosis* H37Rv by Wang *et al.* (2016)[40]. The ncRNA homologs were identified by BLASTn searches (see Methods), and plotted in a heat map for 244 mycobacteria and following the *M. tuberculosis* gene assignment. The "387 core gene" phylogenetic tree (see Fig 2) and clade names are shown to the left and the coloring scheme is the same as in Fig 3. The presence and number of ncRNAs are indicated according to the color legend. ncRNAs marked in red overlap with the Rfam predicted ncRNAs (see main text; Fig 6).

Fig S6

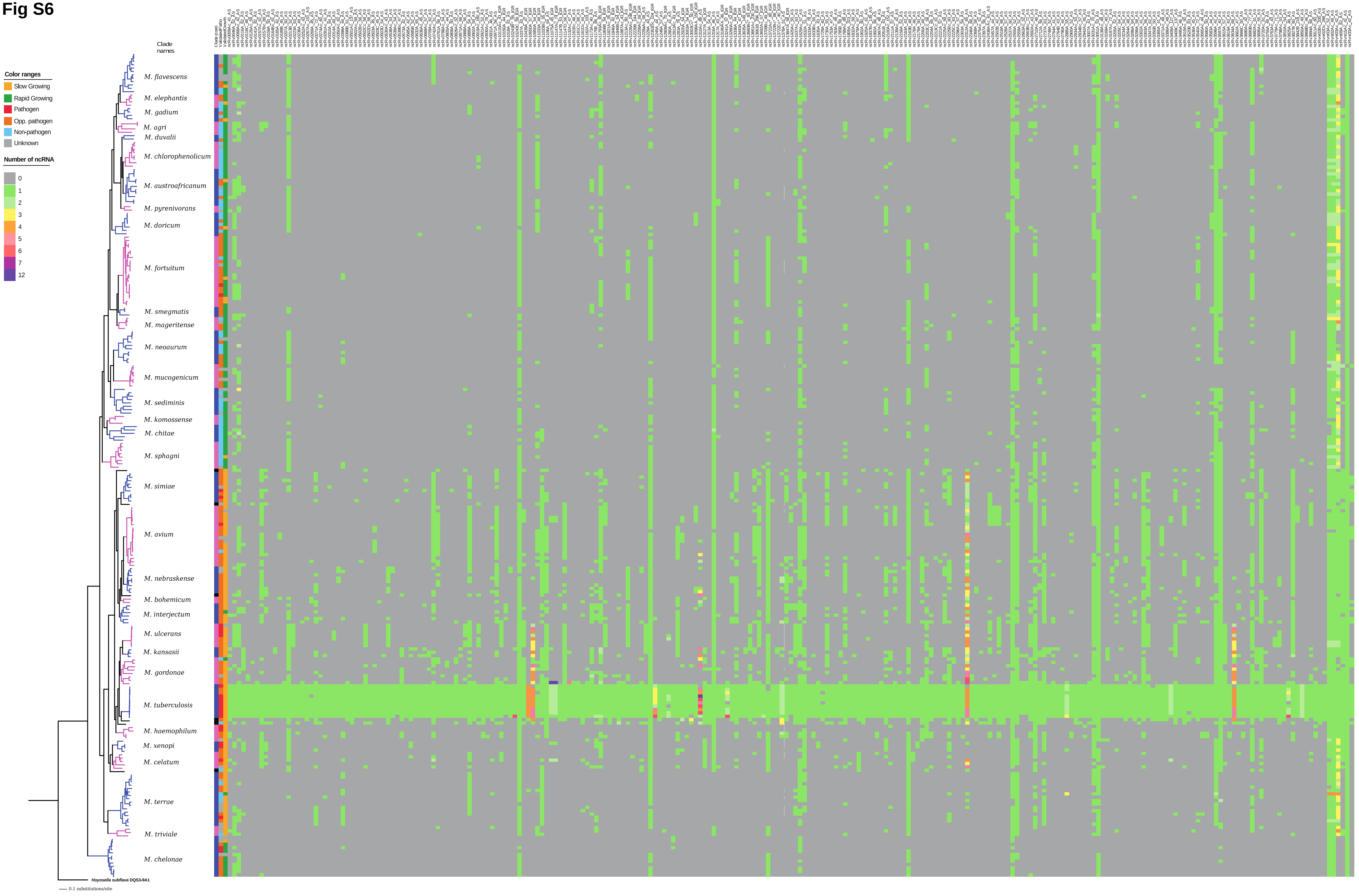
Clade names
Color ranges
Slow Growing
Rapid Growing
Pathogen
Opp. pathogen
Non-pathogen
Unknown
Number of ncRNA
0
1
2
3
4
5
6
7
12
M. flavescens
M. elephantis
M. gadium
M. agri
M. duvalii
M. chlorophenolicum
M. austroafricanum
M. pyrenivorans
M. doricum
M. fortuitum
M. smegmatis
M. mageritense
M. neoaurum
M. mucogenicum
M. sediminis
M. komossense
M. chitae
M. sphagni
M. simiae
M. avium
M. nebraskense
M. bohemicum
M. interjectum
M. ulcerans
M. kansasii
M. gordonae
M. tuberculosis
M. haemophilum
M. xenopi
M. celatum
M. terrae
M. triviale
M. chelonae
Hoyosella subflava DQS3-9A1
0.1 substitutions/site

Figure S7 Analysis of multiple predicted RNase P RNA genes in *M. austroafricanum* DSM 44191

(A) The ncRNA identification detected three putative genes for the RNase P RNA (RPR) subunit in *M. austroafricanum* DSM 44191. A BLASTp analysis identified three genes also in *Mycobacterium* sp. YCRL4 (not included in any of the other analyses) and the gene syntenies were conserved between the two species. RPR genes are indicated with red arrows and the neighboring genes with blue arrows. Brown lines vertically connecting the arrows indicate homologous regions between two species. *M. tuberculosis* H37Rv and *Escherichia coli* MG1655 are shown for comparison.

(B) ClustalX Multiple sequence alignment showing the three *M. austroafricanum* RPR genes, and those from *M. tuberculosis* H37Rv and *Escherichia coli* MG1655.

Fig S7

A

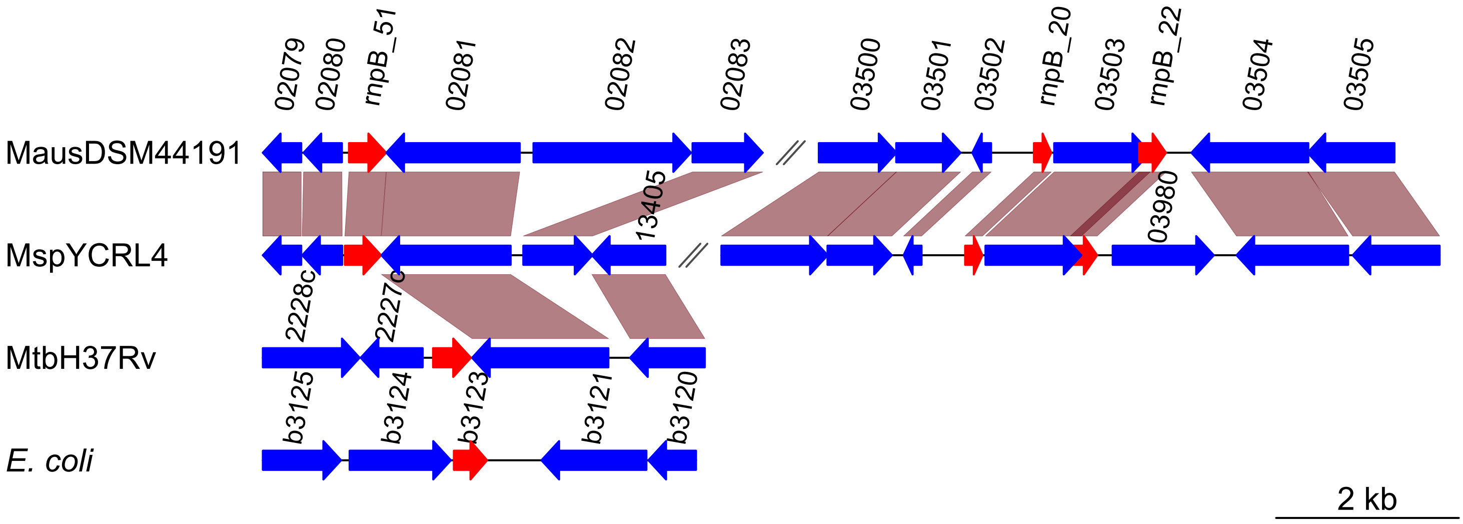

B

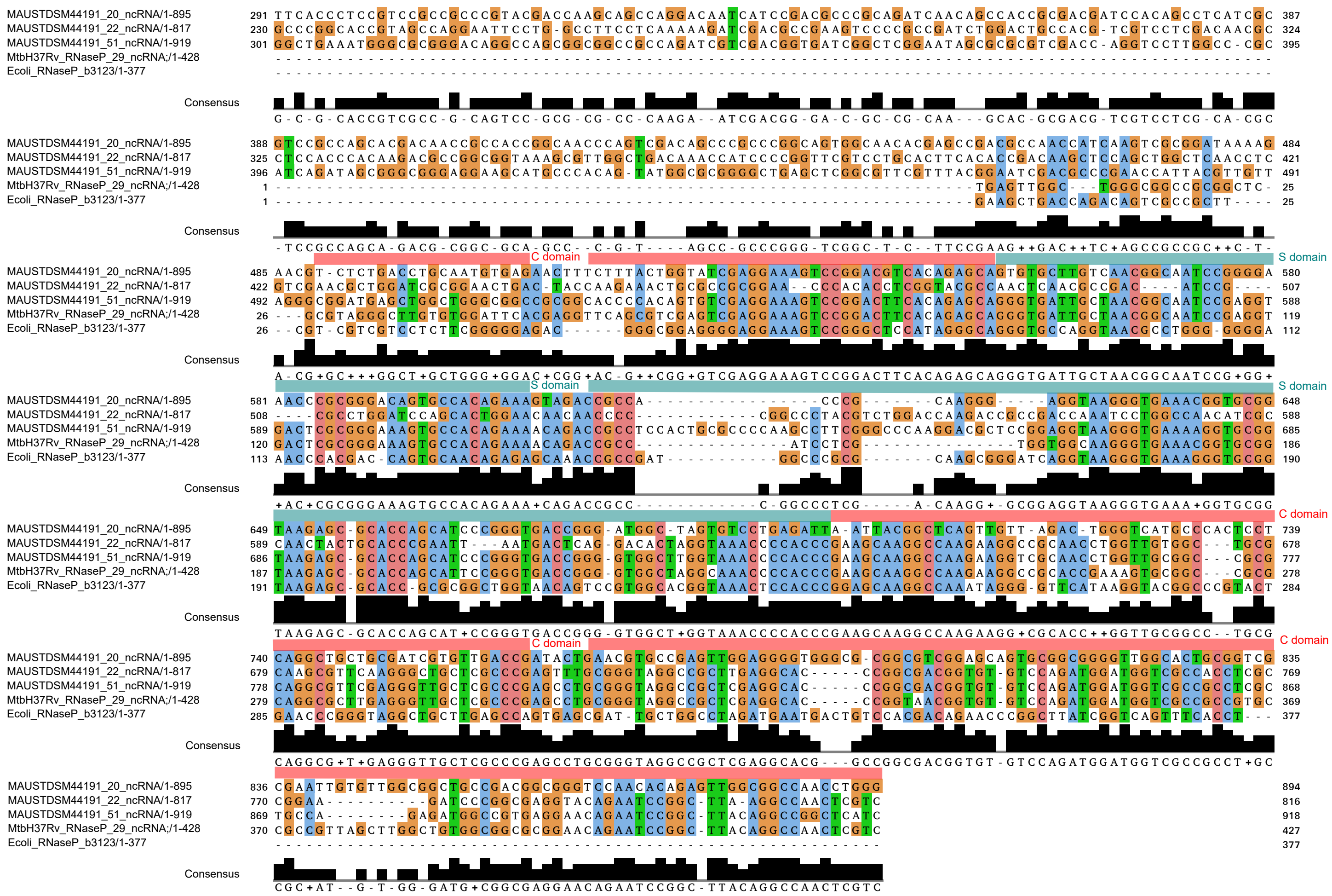

Regular: MAUSTDSM44191_51_ncRNA/1-420
Extra copy1:MAUSTDSM44191_20_ncRNA/1-196
Extra copy2:MAUSTDSM44191_22_ncRNA/1-303

Figure S8 Analysis of multiple predicted tmRNA genes in mycobacteria

Additional tmRNA genes were predicted in four mycobacteria as indicated. Panel (A) shows the nucleotide alignment of the region encoding for the proteolysis tag, along with the tmRNA gene from *M. tuberculosis* H37Rv. Panel (B) shows the amino acid sequence of the proteolysis tag sequence (A). (C) ClustalX multiple sequence alignment represents the regular and extra tmRNA gene sequences from the five mycobacteria as indicated.

Fig S8

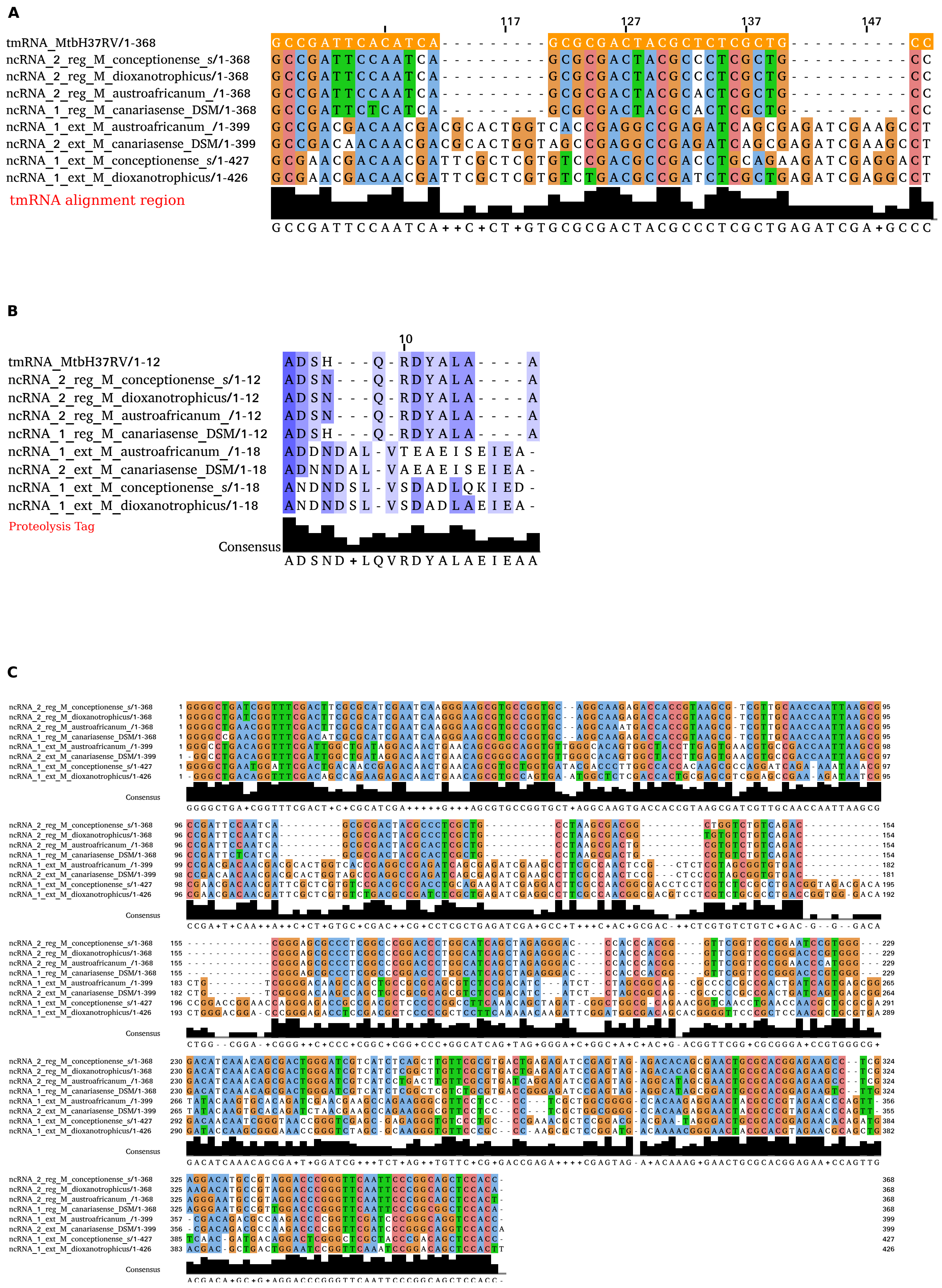
A
117
127
137
147
tmRNA_MtbH37RV/1-368
ncRNA_2_reg_M_conceptionense_s/1-368
ncRNA_2_reg_M_dioxanotrophicus/1-368
ncRNA_2_reg_M_austroafricanum_/1-368
ncRNA_1_reg_M_canariasense_DSM/1-368
ncRNA_1_ext_M_austroafricanum_/1-399
ncRNA_2_ext_M_canariasense_DSM/1-399
ncRNA_1_ext_M_conceptionense_s/1-427
ncRNA_1_ext_M_dioxanotrophicus/1-426
tmRNA alignment region
GCCGATTCCAATCA++C+CT+GTGCGCGACTACGCCCTCGCTGAGATCGA+GCCC
B
10
tmRNA_MtbH37RV/1-12
ncRNA_2_reg_M_conceptionense_s/1-12
ncRNA_2_reg_M_dioxanotrophicus/1-12
ncRNA_2_reg_M_austroafricanum_/1-12
ncRNA_1_reg_M_canariasense_DSM/1-12
ncRNA_1_ext_M_austroafricanum_/1-18
ncRNA_2_ext_M_canariasense_DSM/1-18
ncRNA_1_ext_M_conceptionense_s/1-18
ncRNA_1_ext_M_dioxanotrophicus/1-18
Proteolysis Tag
Consensus
ADSND+LQVRDYALAEIEAA
C
ncRNA_2_reg_M_conceptionense_s/1-368
ncRNA_2_reg_M_dioxanotrophicus/1-368
ncRNA_2_reg_M_austroafricanum_/1-368
ncRNA_1_reg_M_canariasense_DSM/1-368
ncRNA_1_ext_M_austroafricanum_/1-399
ncRNA_2_ext_M_canariasense_DSM/1-399
ncRNA_1_ext_M_conceptionense_s/1-427
ncRNA_1_ext_M_dioxanotrophicus/1-426
Consensus

Figure S9 Analysis of multiple predicted Ms1 RNA genes in *M. nebraskense* and *M. celatum*

(A) GenoPlotR gene synteny plot representing the regular Ms1 RNA gene and the homolog (both marked with red arrows) and adjacent or neighbouring genes (blue arrow). The brown lines vertically connecting the rows (arrows) indicate homologous regions between two species. (B) ClustalX, multiple sequence alignment of the regular (reg) and the extra (ext) Ms1 RNA gene.

Fig S9

A

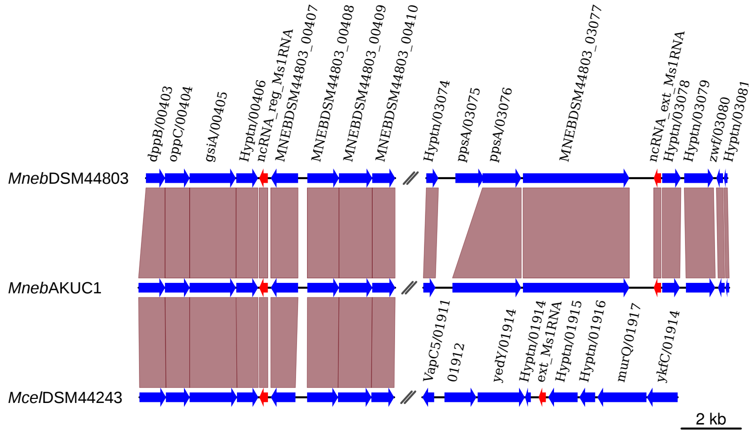
dppB/00403
oppC/00404
gsiA/00405
Hyptn/00406
ncRNA_reg_Ms1RNA
MNEBDSM44803_00407
MNEBDSM44803_00408
MNEBDSM44803_00409
MNEBDSM44803_00410
Hyptn/03074
ppsA/03075
ppsA/03076
MNEBDSM44803_03077
ncRNA_ext_Ms1RNA
Hyptn/03078
Hyptn/03079
zwf/03080
Hyptn/03081
MnebDSM44803
MnebAKUC1
MceIDSM44243
VapC5/01911
01912
yedY/01914
Hyptn/01914
ext_Ms1RNA
Hyptn/01915
Hyptn/01916
murQ/01917
ykfC/01914
2 kb

B

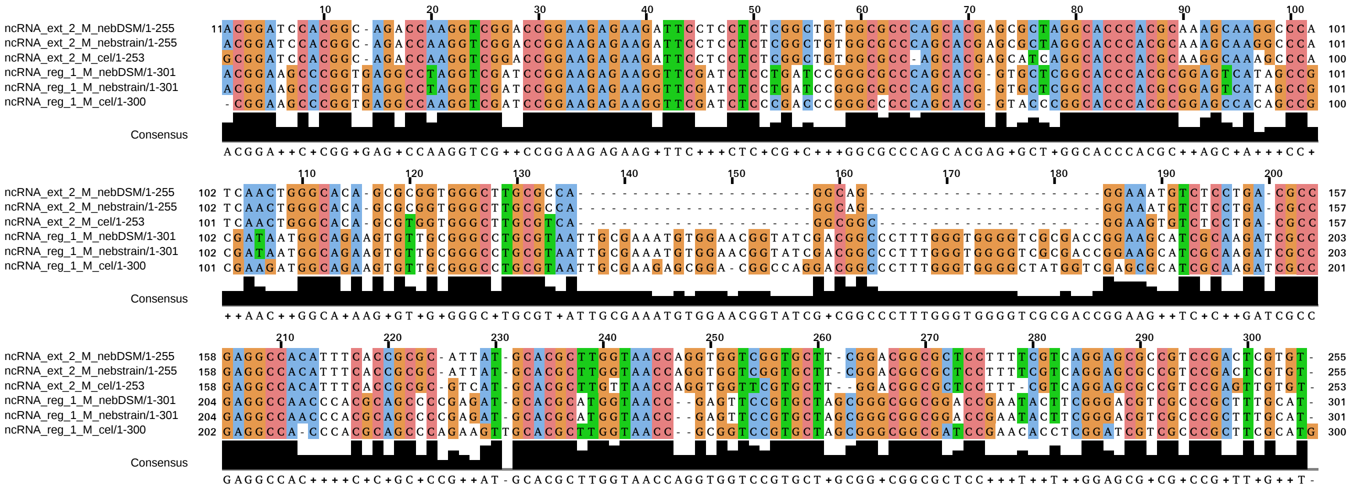
ncRNA_ext_2_M_nebDSM/1-255
ncRNA_ext_2_M_nebstrain/1-255
ncRNA_ext_2_M_cel/1-253
ncRNA_reg_1_M_nebDSM/1-301
ncRNA_reg_1_M_nebstrain/1-301
ncRNA_reg_1_M_cel/1-300
Consensus

Figure S10 Analysis of multiple predicted 6C RNA genes from different mycobacteria

Panels (A-D) represent the genoPlotR gene synteny comparing the homologous 6C RNA genes. The panels represent species having similar gene syntenies. The Ms1 RNA gene (red arrow) is frequently encoded close to the regular 6C RNA gene (reg_6C) and is indicated with a short red arrow. Extra 6C RNA genes (Extra _6C) are also marked with short red arrows. Adjacent or neighbouring genes are shown in blue and the brown lines vertically connecting the rows (arrows) indicate homologous regions between two species. Double slashes (//) indicate that the regions are not immediately adjacent to each other. Panel (E) shows a multiple sequence alignment of 6C RNA from different mycobacteria.

Fig S10

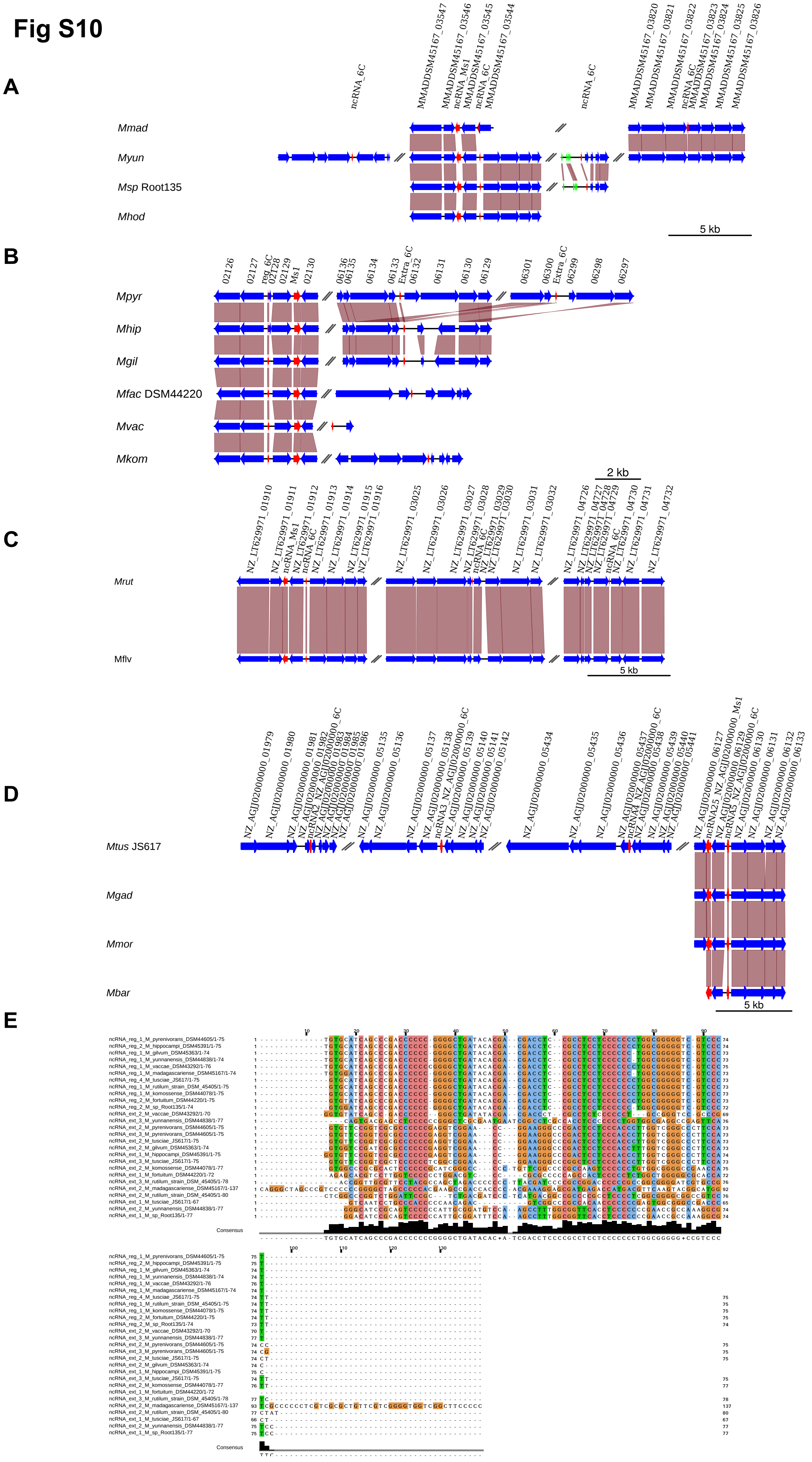

A
ncRNA_6C
MMADDSM45167_03547
MMADDSM45167_03546
ncRNA_Ms1
MMADDSM45167_03545
ncRNA_6C
MMADDSM45167_03544
MMADDSM45167_03820
MMADDSM45167_03821
MMADDSM45167_03822
MMADDSM45167_03823
MMADDSM45167_03824
MMADDSM45167_03825
MMADDSM45167_03826
Mmad
Myun
Msp Root135
Mhod
5 kb
B
02126
02127
reg_6C
02128
02129
Ms1
02130
06136
06135
06134
06133
Extra_6C
06132
06131
06130
06129
06301
06300
Extra_6C
06299
06298
06297
Mpyr
Mhip
Mgil
Mfac DSM44220
Mvac
Mkom
2 kb
C
NZ_LT629971_01910
NZ_LT629971_01911
ncRNA_Ms1
NZ_LT629971_01912
ncRNA_6C
NZ_LT629971_01913
NZ_LT629971_01914
NZ_LT629971_01915
NZ_LT629971_01916
NZ_LT629971_03025
NZ_LT629971_03026
NZ_LT629971_03027
NZ_LT629971_03028
ncRNA_6C
NZ_LT629971_03029
NZ_LT629971_03030
NZ_LT629971_03031
NZ_LT629971_03032
NZ_LT629971_04726
NZ_LT629971_04727
NZ_LT629971_04728
NZ_LT629971_04729
ncRNA_6C
NZ_LT629971_04730
NZ_LT629971_04731
NZ_LT629971_04732
Mrut
Mflv
5 kb
D
Mtus JS617
Mgad
Mmor
Mbar
5 kb
E
Consensus

Figure S11 Analysis of multiple predicted *ydaO_yuaA* in *M. kansasii*

(A) GenoPlotR gene synteny plot showing the *ydaO_yuaA* homolog (red arrows) and adjacent or neighbouring genes (blue arrows). The brown lines vertically connecting the rows (arrows) indicate homologous regions between two species. Double slashes (//) indicate that the regions are not immediately adjacent to each other. (B) ClustalX, multiple sequence alignment of regular (reg) and the extra (ext) *ydaO_yuaA*.

Fig S11

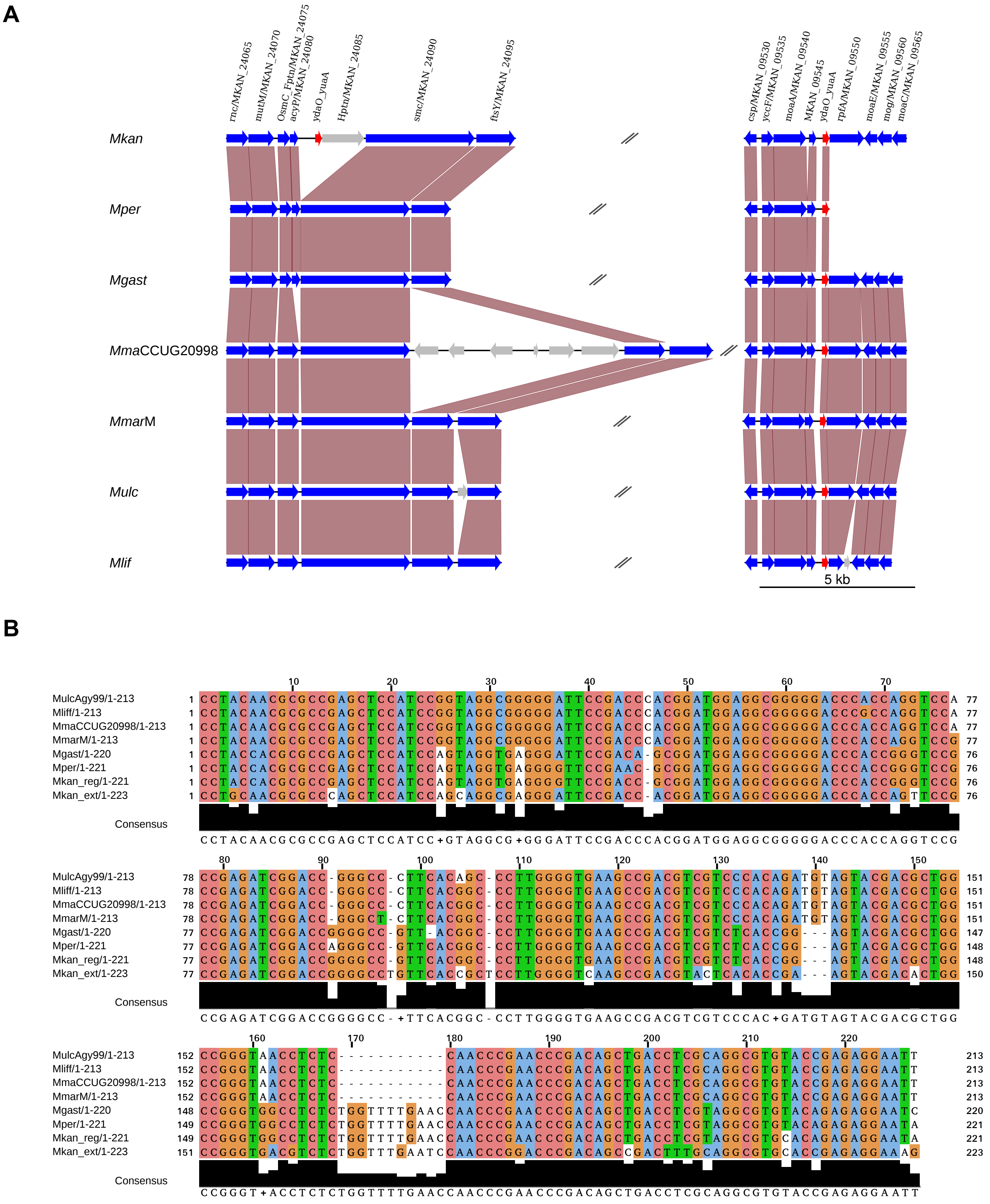
A
rnc/MKAN_24065
mutM/MKAN_24070
OsmC_Fptn/MKAN_24075
acyP/MKAN_24080
ydaO_yuaA
Hptn/MKAN_24085
smc/MKAN_24090
ftsY/MKAN_24095
csp/MKAN_09530
yccF/MKAN_09535
moaA/MKAN_09540
MKAN_09545
ydaO_yuaA
rpfA/MKAN_09550
moaE/MKAN_09555
mog/MKAN_09560
moaC/MKAN_09565
Mkan
Mper
Mgast
MmaCCUG20998
MmarM
Mulc
Mlif
5 kb
B
MulcAgy99/1-213
Mliff/1-213
MmaCCUG20998/1-213
MmarM/1-213
Mgast/1-220
Mper/1-221
Mkan_reg/1-221
Mkan_ext/1-223
Consensus

Figure S12 Genome alignment and Venn diagrams of *M. abscessus, M. chelonae, M. salmoniphilum, H. subflava, and S. rotundus*

(A) Whole genome alignment for *M. chelonae*, *M. salmoniphilum*, *M. abscessus* (strain ATCC19977), *H. subflava* and *S. rotundus* as indicated. Vertical lines (orange) between genomes correspond to homologous regions and diagonal lines (blue) correspond to genome rearrangements.

(B-D) Pairwise comparisons as indicated (excluding *M. salmoniphilum*). *M. abscessus* = *Mabs*, *M. chelonae* = *Mche*, *M. salmoniphilum* = *Msal*, *H. subflava* = *Hsub* and *S. rotundus* = *Srot*. The number of common and unique genes for the five species are indicated.

(E-G) is a four-species comparison for the indicated genomes.

Fig S12

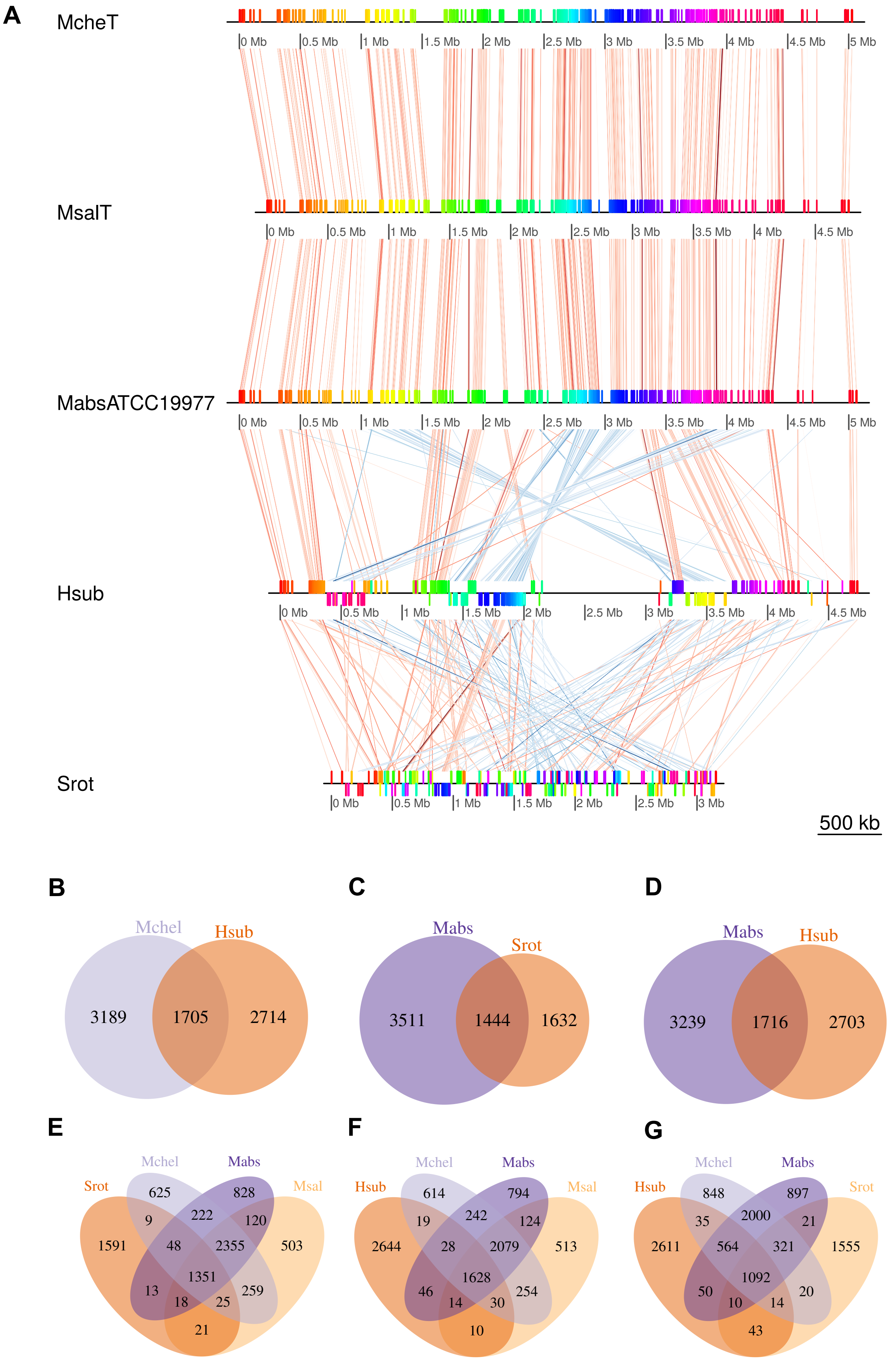
A
McheT
MsalT
MabsATCC19977
Hsub
Srot
500 kb
B
Mchel
Hsub
3189
1705
2714
C
Mabs
Srot
3511
1444
1632
D
Mabs
Hsub
3239
1716
2703
E
Srot
Mchel
Mabs
Msal
625
828
9
222
120
1591
48
2355
503
1351
13
259
18
25
21
F
Hsub
Mchel
Mabs
Msal
614
794
19
242
124
2644
28
2079
513
1628
46
254
14
30
10
G
Hsub
Mchel
Mabs
Srot
848
897
35
2000
21
2611
564
321
1555
1092
50
20
10
14
43

Figure S13 Comparison with previously published mycobacterial phylogenies

The "387 core gene" phylogenetic tree (Fig 2) collapsed to the clade level is shown in the middle. Green and orange background shading indicate RGM and SGM clades, respectively (exceptions to the division into RGM and SGM have been ignored in this tree; for details, see Fig 2). Alternating magenta and blue tree branches indicate alternating clades where triangles at the tips mark collapsed clades. Black branches with black dots at the tips indicate single-species clades. To the left is the "Matsumoto soft-core gene tree"[18] (orange) while the two trees to the right are based on 1941 core proteins (magenta)[16] and Average Nucleotide Identity, ANI (blue)[15,17]. Arrows indicate differences in comparison to the "387 core gene" tree (Fig 2). "From" indicates the clade in the "387 core gene" tree to which the indicated species belongs. "To" indicates the clades assigned for the marked species using the other phylogenetic trees as indicated. Colored (dotted) boxes represent unique clades (depending upon which phylogenetic tree is used). Black dotted box indicates an alternative clade grouping; clades inside the box can be regarded as individual clades or merged into a single, larger clade. Mycobacteria marked with colored dots (• and •) in front of the name represent single species clades in the respective tree.

Fig S13

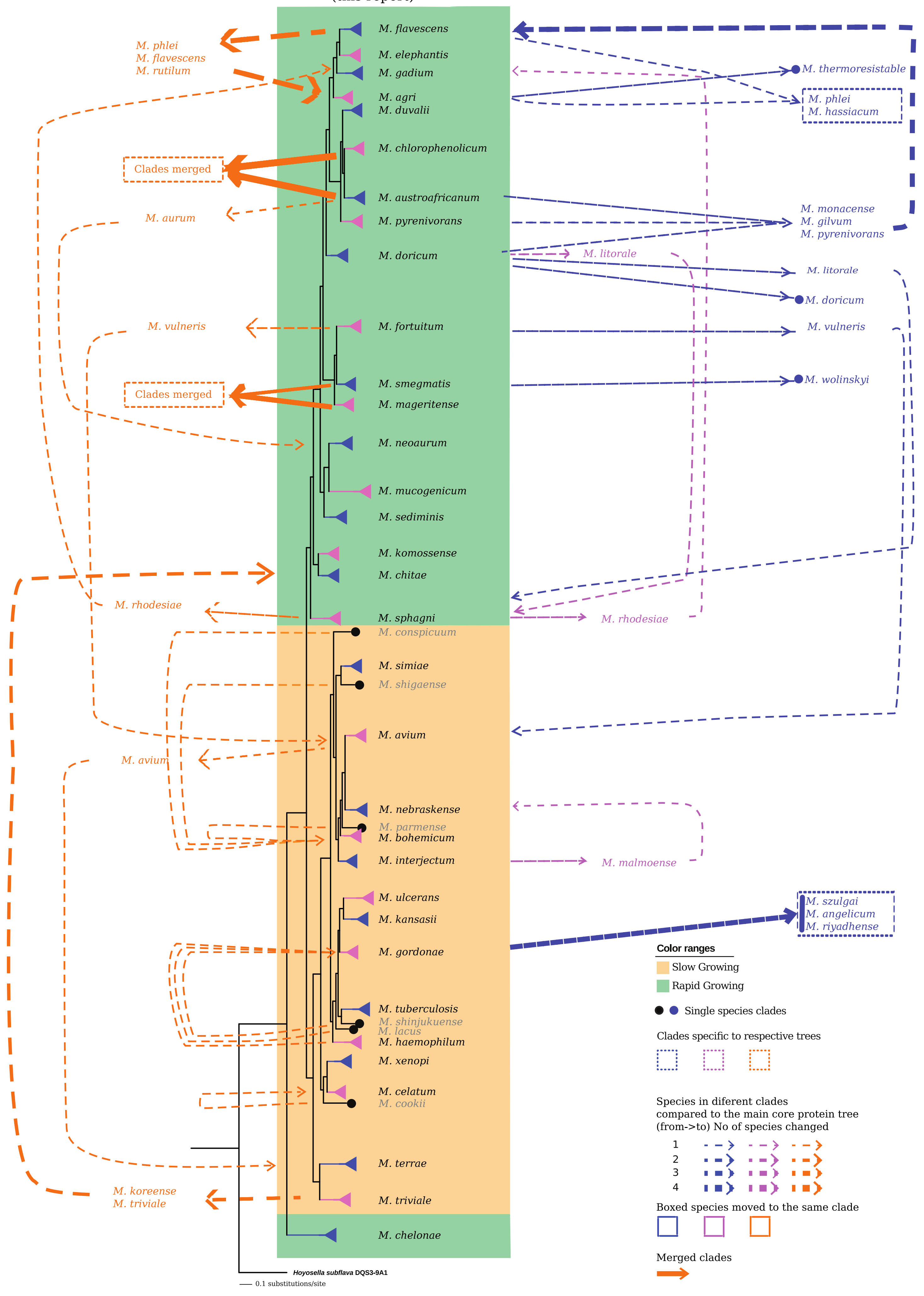

Matsumoto et al.
(1306 core genes)
Core tree
387 core proteins
(this report)
Gupta et al.
(1941core proteins)
Tortoli et al.
(ANI)
M. flavescens
M. elephantis
M. gadium
M. agri
M. duvalii
M. chlorophenolicum
M. austroafricanum
M. pyrenivorans
M. doricum
M. fortuitum
M. smegmatis
M. mageritense
M. neoaurum
M. mucogenicum
M. sediminis
M. komossense
M. chitae
M. sphagni
M. conspicuum
M. simiae
M. shigaense
M. avium
M. nebraskense
M. parmense
M. bohemicum
M. interjectum
M. ulcerans
M. kansasii
M. gordonae
M. tuberculosis
M. shinjukuense
M. lacus
M. haemophilum
M. xenopi
M. celatum
M. cookii
M. terrae
M. triviale
M. chelonae
Hoyosella subflava DQS3-9A1
0.1 substitutions/site
M. phlei
M. flavescens
M. rutilum
Clades merged
M. aurum
M. vulneris
Clades merged
M. rhodesiae
M. avium
M. koreense
M. triviale
M. litorale
M. rhodesiae
M. malmoense
M. thermoresistable
M. phlei
M. hassiacum
M. monacense
M. gilvum
M. pyrenivorans
M. litorale
M. doricum
M. vulneris
M. wolinskyi
M. szulgai
M. angelicum
M. riyadhense
Color ranges
Slow Growing
Rapid Growing
Single species clades
Clades specific to respective trees
Species in diferent clades
compared to the main core protein tree
(from->to) No of species changed
1
2
3
4
Boxed species moved to the same clade
Merged clades

Figure S14 Distribution of tRNA genes in different bacterial phyla

The number of tRNA genes for different bacteria were obtained from the genomic tRNA database and plotted in groups representing different phyla or classes. Box plots were used where the thick horizontal black line represents the median tRNA gene number. The upper and lower bounds of the boxes represent the second and third quartiles, and the whiskers represent the first and fourth quartiles. Outliers are represented as circles. The n-values represent the number of species in each group of bacteria. n = represents number of bacterial species in the respective phyla.

Fig S14

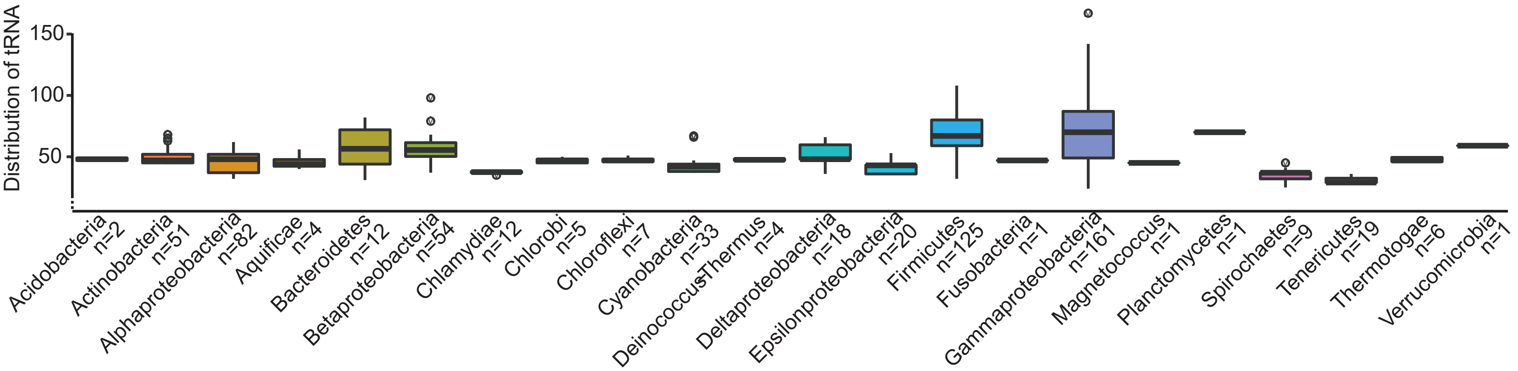
Distribution of tRNA
150
100
50
Acidobacteria n=2
Actinobacteria n=51
Alphaproteobacteria n=82
Aquificae n=4
Bacteroidetes n=12
Betaproteobacteria n=54
Chlamydiae n=12
Chlorobi n=5
Chloroflexi n=7
Cyanobacteria n=33
Deinococcus-Thermus n=4
Deltaproteobacteria n=18
Epsilonproteobacteria n=20
Firmicutes n=125
Fusobacteria n=1
Gammaproteobacteria n=161
Magnetococcus n=1
Planctomycetes n=1
Spirochaetes n=9
Tenericutes n=19
Thermotogae n=6
Verrucomicrobia n=1

Figure S15 Multiple sequence alignment of 6S RNA like homologs in *Streptomyces coelicolor* A3 2 (SCO) and Ms1 RNA homologs of selected *Corynebacterineae* species

Multiple sequence alignment was performed using clustalX (v2.1) and the program Jalview (v2.7) for visualization. Five species belonging to the *Corynebacterineae* suborder carry two Ms1 RNA gene copies [the regular Ms1 RNA gene is shown as (1) and the extra Ms1 RNA copy as (2)] *Mycobacterium celatum* DSM 44243 (*Mcel*), *Mycobacterium nebraskense* AKUC1 (*Mneb*AKUC1), *Mycobacterium nebraskense* DSM 44803 (*Mneb*DSM), *Rhodococcus jostii* (*Rjos*), *Smaragdicoccus niigatensis* DSM44881 NBRC103563 (*Snii*). The other species contain a single Ms1 RNA gene:

Mycobacterium leprae TN (*Mlep*TN), *Mycobacterium mucogenicum* DSM 44124 (*Mmuc*), *Mycobacterium smegmatis* MC2 155 (*Msmeg*), *Gordonia bronchialis* DSM43247 (*Gbro*), *Hoyosella subflava* DQS3-9A1 (*Hsub*), *Millisia brevis* NBRC105863 (*Mbre*), *Rhodococcus fascians* D188 (*Rfas*), *Rhodococcus sp.* S2-17 plasmid pRB98 (Rsp_S217_pRB98), *Segniliparus rotundus* DSM44985 (*Srot*), *Skermania piniformis* NBRC15059 (*Spin*), *Tsukamurella paurometabola* DSM20162 (*Tpau*) and *Williamsia sterculiae* (*Wste*).

Fig S15

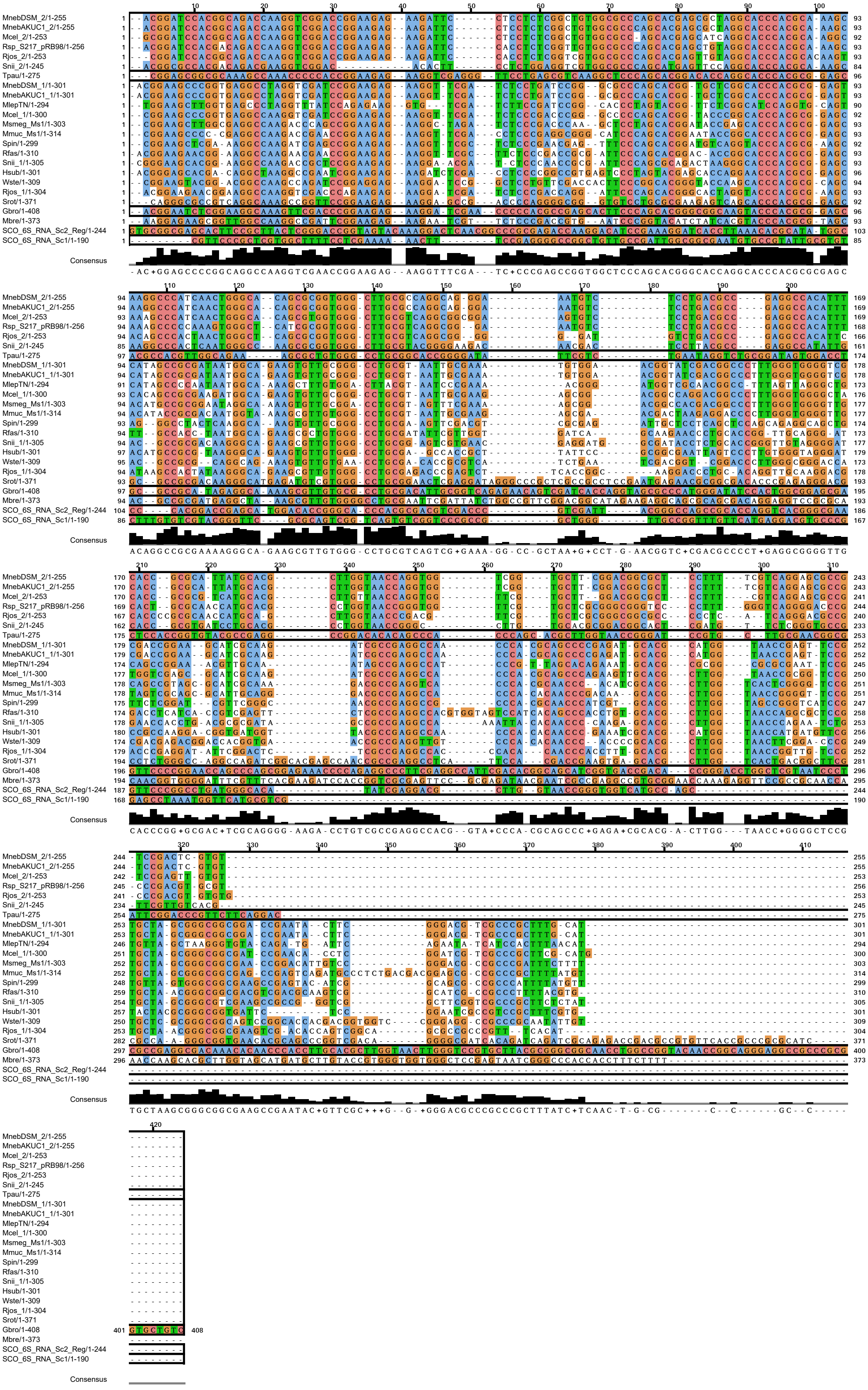
MnebDSM_2/1-255
MnebAKUC1_2/1-255
Mcel_2/1-253
Rsp_S217_pRB98/1-256
Rjos_2/1-253
Snii_2/1-245
Tpau/1-275
MnebDSM_1/1-301
MnebAKUC1_1/1-301
MlepTN/1-294
Mcel_1/1-300
Msmeg_Ms1/1-303
Mmuc_Ms1/1-314
Spin/1-299
Rfas/1-310
Snii_1/1-305
Hsub/1-301
Wste/1-309
Rjos_1/1-304
Srot/1-371
Gbro/1-408
Mbre/1-373
SCO_6S_RNA_Sc2_Reg/1-244
SCO_6S_RNA_Sc1/1-190
Consensus
